# A Revised Modular Approach to (–)‐*trans*‐Δ^8^‐THC and Derivatives Through Late‐Stage Suzuki–Miyaura Cross‐Coupling Reactions

**DOI:** 10.1002/ejoc.201900059

**Published:** 2019-03-18

**Authors:** Victor R. L. J. Bloemendal, Daan Sondag, Hidde Elferink, Thomas J. Boltje, Jan. C. M. van Hest, Floris P. J. T. Rutjes

**Affiliations:** ^1^ Institute for Molecules and Materials Heyendaalseweg 135 NL‐6525 AJ Nijmegen The Netherlands; ^2^ Eindhoven University of Technology P.O. Box 513 (STO 3.31) NL‐5600 MB Eindhoven The Netherlands

**Keywords:** Tetrahydrocannabinol, Suzuki–Miyaura coupling, Cannabis derivatives, Cascade reactions, Heterocycles

## Abstract

A revised modular approach to various synthetic (–)‐*trans*‐Δ^8^‐THC derivatives through late‐stage Suzuki–Miyaura cross‐coupling reactions is disclosed. Ten derivatives were synthesized allowing both sp^2^‐ and sp^3^‐hybridized cross‐coupling partners with minimal β‐hydride elimination. Importantly, we demonstrate that a *para*‐bromo‐substituted THC scaffold for Suzuki–Miyaura cross‐coupling reactions has been initially reported incorrectly in recent literature.

## Introduction

**Scheme 1:**
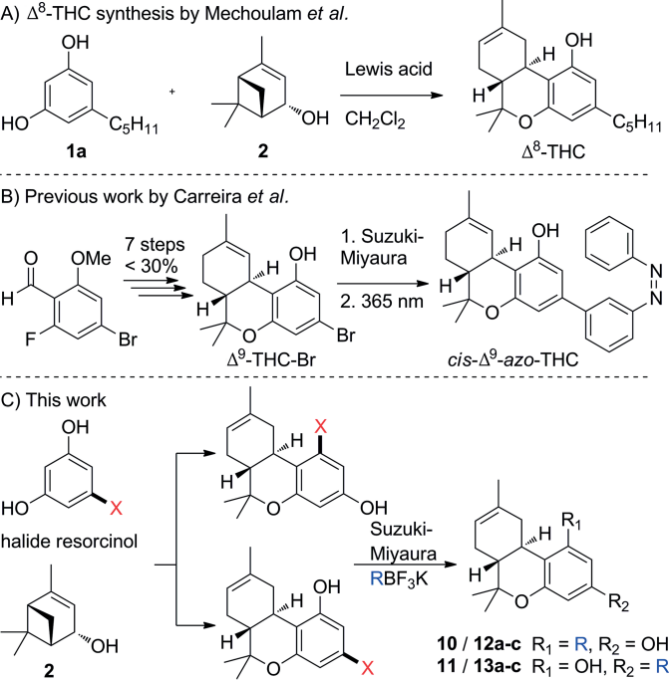
A) Synthesis of (–)‐*trans*‐Δ^8^‐THC using (–)‐verbenol (**2**) and olivetol (**1a**) by Mechoulam et al.;^9^ B) Synthesis of (–)‐*trans*‐Δ^9^‐THC‐Br using multistep synthesis by Carreira et al.;^10^ C) Our revised modular synthesis of (–)‐*trans*‐Δ^8^‐THC derivatives.

Medicinal applications of Cannabis sativa have drawn worldwide attention ever since the first introduction in Western medicine in 1839.^1^ Since then, over 500 constituents from this plant have been isolated and identified, among which 113 biologically active phytocannabinoids.^2^ The active constituents may be applied to treat neurodegenerative symptoms of Parkinson, Alzheimer, and MS,^2^ but are also used as analgesic for patients with specific forms of cancer.^3^ Tetrahydrocannabinols (THCs), in particular the predominant isomers (–)‐*trans*‐Δ^8^‐THC (thermodynamic product, Scheme 1A) and (–)‐*trans*‐Δ^9^‐THC (kinetic product), are the major (psycho‐)active compounds encountered in Cannabis sativa.^4^ THCs interact with the G‐protein‐coupled receptors CB_1_ and CB_2_, which are mainly expressed in the central nervous system (CNS) and its periphery.^5^ The pharmacological effects and selectivity exhibited by the natural substrates may be improved by synthetic THC derivatives.^6^ Hence, a multitude of synthetic CB_1_ agonists have already been prepared, some of which are in clinical trials.^6^, ^7^

The first isolation and partial synthesis of (–)‐*trans*‐Δ^9^‐THC (Δ^9^‐THC) in 1964 by Mechoulam et al.,^8^ followed by the stereoselective synthesis of both THC isomers three years later,^9^ initiated a growing interest in the preparation of new (synthetic) cannabinoids (Scheme 1A). In particular, the introduction of unnatural substituents on the resorcinol building block was shown to improve selectivity of THC analogues for CB_1_ or CB_2_. Despite various strategies that have been developed over the years,^1^ the synthesis of THC derivatives remains a significant challenge. Therefore, a generally applicable modular approach allowing late‐stage synthetic modification of cannabinoids would be very useful. As an example, an elegant method to synthesize challenging Δ^9^‐THC derivatives via late‐stage Suzuki–Miyaura cross‐coupling reactions was recently reported by Carreira et al. (Scheme 1B).^10^ Yet, the preparation of the Δ^9^‐THC‐Br precursor required a multistep sequence and did not provide access to the corresponding (–)‐*trans*‐Δ^8^‐THC (Δ^8^‐THC) derivatives.^6^

Herein we report a revised one‐step synthetic approach to Δ^8^‐THC, Δ^8^‐propyl‐THC and halogenated Δ^8^‐THC scaffolds, which have been used in SAR studies.^6^ We also demonstrate that recent reports concerning the synthesis of para‐substituted THC derivatives are incorrect,^11^ and by studying the regioselectivity of various resorcinol derivatives with (–)‐verbenol (**2**) we deliver proof of the correct assignment of the two possible regioisomers. Finally, both regioisomeric scaffolds were functionalized through late‐stage Suzuki–Miyaura cross‐coupling reactions with sp^2^‐ and sp^3^‐hybridized organoboron reagents (Scheme 1C).

## Results and Discussion

**Scheme 2:**
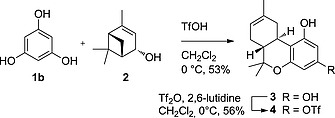
Synthesis of Δ^8^‐THC‐triflate (**4**) using phloroglucinol (**1b**) and subsequent regioselective triflation.

Inspired by the seminal work of Mechoulam et al. we investigated whether the electrophilic aromatic substitution of commercially available olivetol (**1a**) with (–)‐verbenol (**2**), directly followed by cyclization to afford Δ^8^‐THC could also be effected with Brønsted acids (see: Experimental Section). Reaction under the influence of TfOH in CH_2_Cl_2_ at 0 °C provided the thermodynamic isomer Δ^8^‐THC in 33 % isolated yield as the sole product. Unlike weaker Brønsted acids, TfOH was successfully used for both Friedel‐Crafts alkylation and subsequent cyclization at room temperature. We also envisioned that this transformation could be used to create a Δ^8^‐THC scaffold for late‐stage derivatization through Pd‐catalyzed cross‐coupling reactions. Thus, initially using readily available phloroglucinol (**1b**), Δ^8^‐THC‐hydroxy analogue **3** was prepared using TfOH in 53 % yield (Scheme 2). Selective triflation with Tf_2_O at 0 °C of the least hindered *para*‐hydroxy substituent resulted in Δ8‐THC‐triflate **4** in 56 % yield.

Unfortunately, all attempts of triflate **4** to undergo sp^2^‐sp^3^ Suzuki–Miyaura coupling utilizing various ligands, solvents and different organoboron reagents failed to give the desired products (see: Supporting Information I). Presumably, oxidative addition onto the electron‐rich aromatic system did not occur, since in most cases triflate **4** was recovered.^12^ During the preparation of this manuscript, Studer et al. reported the sp^2^‐sp^2^ Suzuki–Miyaura cross coupling with triflate **4** to obtain aryl‐substituted THC derivatives,^13^ but were unable to prepare biologically more relevant sp^3^‐substituted THC derivatives^14^ through direct cross‐coupling reactions.

**Scheme 3:**
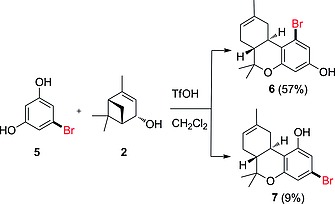
Reaction of 5‐bromoresorcinol (**5**) with (–)‐verbenol (**2**) to give regioisomers **6** and **7**.

Inversely, existing syntheses of bromo‐substituted THCs^11^, ^15^ by alkylating 5‐bromoresorcinol **5** with terpenoid systems such as verbenol (**2**) and *para*‐mentha‐2,8‐dienol, inspired us to incorporate different synthetic handles in the Δ^8^‐THC derivatives. Hence, halide‐substituted THC scaffolds were prepared through TfOH‐catalyzed condensation of resorcinol **5** (Scheme 3).

**Figure 1:**
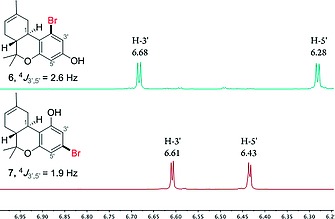
The ^1^H‐NMR chemical shift and ^4^ *J*_3′‐5′_‐coupling constants of the aromatic protons of regioisomers **6** and **7**.

The electrophilic aromatic substitution/cyclization protocol of **5** with (–)‐verbenol (**2**) surprisingly provided different results than recently published by Studer et al.[11a] and Dethe et al.[11b] (Scheme 3). In our hands, a mixture of regioisomers **6** and **7** was obtained, with the *ortho*‐substituted regioisomer **6** being the main product, meaning that electrophilic aromatic substitution of **5** did not only take place on the “activated” C2‐position but also on the equivalent C4‐ and C6‐positions.^16^ The Dethe and Studer groups reported formation of the *para*‐isomer **7** as the sole product, however, the structure was initially incorrectly assigned. Our characterizations are in line with the *para*‐bromo‐substituted Δ^9^‐THC derivatives by Carreira et al.,^10^ describing similar NMR shifts and coupling constants. The discrepancy in the assignment of the regioisomers was clarified using a variety of NMR experiments (see: Supporting Information II). Careful analysis of the ^1^H‐NMR spectrum showed clear proof of the difference between regioisomers **6** and **7**, indicated by a 0.7 Hz difference in ^4^ *J*_3′,5′_ coupling constant between the two aromatic protons and their distinguishable chemical shifts (Figure 1). This was further confirmed by HMBC NMR analysis showing a correlation between proton H‐1 and C‐2′.

Since the undesired regioisomer was formed predominantly, we studied the intrinsic regioselectivity of the electrophilic aromatic substitution hoping that by changing the halide of the resorcinol system the ratio could be positively influenced. Starting from 5‐chloro‐ and 5‐iodoresorcinol (**8** and **9**, respectively) four halide‐substituted THC analogues **16**/**17** and** 18**/**19** were prepared. Despite the difference in size of the halides, no clear trend in regioselectivity was observed, since in all cases *ortho*‐substitution was preferred over *para*‐substitution. This preference has also been observed in literature,^16^, ^17^ and is most likely due to the deactivating effect exerted by the halide on the aromatic ring. Selective *para*‐substitution was only observed in case of the alkyl‐substituted THC regioisomers **13a** and **13b**. This is underlined by Baek et al.,^18^ who already showed in 1992 that electrophilic aromatic substitution of alkyl resorcinols preferentially takes place at the C2‐position. For the halide‐substituted THC analogues the highest amount of *para‐*substitution and total yield were obtained starting from 5‐bromoresorcinol (**5**, Table 1, entry 2). These bromo‐substituted synthons for Suzuki–Miyaura cross‐coupling reactions were used to derivatize the pharmacologically relevant C3′‐ and C5′‐positions of Δ^8^‐THC.^19^

**Table 1:**
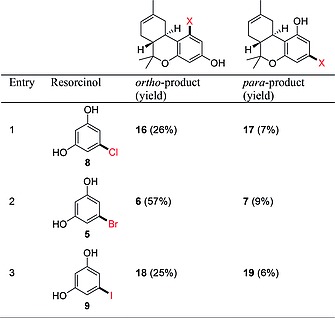
*ortho‐* and *para‐*halide substituted THCs obtained from resorcinols **5**, **8** and **9**

**Scheme 4:**
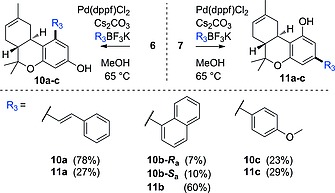
The Suzuki–Miyaura cross‐coupling of isomers **6** and **7** to give Δ^8^‐THC derivatives using sp^2^‐hybridized organotrifluoroborate substrates.

To investigate the reactivity of bromides **6** and **7**, various Pd‐catalyzed cross‐coupling reactions were evaluated. Classical Heck, Kumada, Stille, and Negishi reactions were investigated, but all led to degradation of the THC scaffold, were low yielding and/or hard to reproduce. The Suzuki–Miyaura cross‐couplings of **6** and **7** were successful and provided six different Δ^8^‐THC derivatives (Scheme 4). Use of Pd(dppf)Cl_2_ as the catalyst in combination with Cs_2_CO_3_, MeOH and potassium trifluoroborates (BF_3_K salts)^10^ worked best in our hands and afforded the products **10a–c** and **11a–c** in yields ranging from 17 up to 78 %. NMR data of the *ortho*‐substituted derivatives **10a–c** were in agreement with those obtained in earlier studies,^13^ although they were previously reported to be *para*‐substituted (see: Supporting Information III). Notably, **10b** was formed as an inseparable mixture of atropisomers (*R*_a_, *S*_a_), but could be analyzed using advanced NMR techniques (see: Supporting Information IV).

**Scheme 5:**
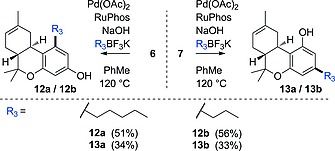
The Suzuki–Miyaura cross‐coupling of isomers **6** and **7** to give Δ^8^‐THC (derivatives) using sp^3^‐hybridized organotrifluoroborate substrates.

To extend this method to a modular approach, we studied conditions that would allow the synthesis of more challenging substrates involving sp^2^‐sp^3^ cross‐coupling. It was found that Pd(OAc)_2_ combined with RuPhos and NaOH facilitated coupling with sp^3^‐hybridized reagents with minimal β‐hydride elimination.^20^ The BF_3_K salts, used as substrates for cross‐coupling reactions, were prepared in a straightforward manner from the corresponding boronic acids under non‐etching conditions.^21^ Elaborating on the essential difference of regioisomers **6** and **7**, we converted **7** into naturally occurring Δ^8^‐THC (**13a**) and Δ^8^‐propyl‐THC (**13b**) by successful Suzuki–Miyaura cross‐coupling (Scheme 5). The spectroscopic data of **13a** and **13b** were in agreement with previously conducted experiments (see: Experimental Section). The versatility of this new modular route towards Δ^8^‐THC was extended to the preparation of THC derivatives **12a**–**b**.

## Conclusions

In conclusion, we developed a synthetically versatile experimental procedure to synthesize Δ^8^‐THC and a range of derivatives. Six unique halide‐substituted THC analogues were prepared through an electrophilic aromatic substitution/cyclization protocol of three different halide resorcinols with verbenol, which are scaffolds for Suzuki–Miyaura cross‐coupling reactions. Regioselectivity of the Friedel‐Crafts alkylations was evaluated and shown to be primarily *ortho*‐directing, most likely due to electronic effects. The use of bromo‐substituted Δ^8^‐THC in recent literature was wrongly reported to provide *para*‐substituted products and is rectified. Our revised modular approach proved to be suitable for sp^2^‐ and sp^3^‐hybridized substrates and led to the synthesis of ten different pharmacologically relevant Δ^8^‐THC derivatives. We envision that this modular procedure can be extended to Δ^9^‐THC derivatives using double bond isomerization^22^ or starting from *para*‐mentha‐2,8‐dien‐1‐ol, which is currently being studied in our laboratories.

## Experimental Section

**Supporting Information** (see footnote on the first page of this article): copies of 1D and 2D NMR spectra and extensive NMR studies are provided in Supporting Information.

**1. General information**: NMR spectra were recorded on a Bruker Avance III 400 MHz or a Bruker 500 MHz spectrometer and the compounds were assigned using ^1^H NMR, ^13^C NMR, ^11^B NMR, ^19^F NMR, COSY, HSQCED and HMBC spectra. Chemical shifts were reported in parts per million (ppm.) relative to reference (CDCl_3_: ^1^H: 7.26 ppm. and ^**1**3^C 77.16 ppm; CD_3_OD: ^1^H: 3.31 ppm. and ^**1**3^C 49.00 ppm; (CD_3_)_2_SO: ^1^H: 2.50 ppm. and ^**1**3^C 39.52 ppm.) NMR data are presented in the following way: chemical shift, multiplicity (s = singlet, bs = broad singlet, d = doublet, *t* = triplet, dd = doublet of doublets, ddd = doublet of doublet of doublets, dtd = doublet of triplet of doublets h = heptet, m = multiplet and/or multiple resonances) and coupling constants *J* in Hz. Reactions were monitored using TLC F_254_ (Merck KGaA) using UV absorption detection (254 nm) and by spraying them with cerium ammonium molybdate stain (Hannesian's stain) followed by charring at ca 300 °C. Mass spectra were recorded on a JEOL AccuTOF CS JMS‐T100CS (ESI) mass spectrometer. Melting points (m.p.) were determined using a Büchi Melting Point B‐545. Automatic flash column chromatography was executed on a Biotage Isolera Spektra One using SNAP or Silicycle cartridges (Biotage, 30–100 μm, 60Å) 4–50 g. Reactions under protective atmosphere were performed under positive Ar./N_2_ flow in flame‐dried flasks. Atom‐numbering of the THC compounds is derived from an earlier reported NMR assignment in literature.^19^

**2. General procedures**

**General procedure I** for potassium trifluoroborate salt synthesis from boronic acid (**22–25**)**:** 21 Boronic acid (1 equiv.) was dissolved in acetonitrile (0.1M), KF (4 equiv.) in water (1M) was added at r.t. and the reaction was left stirring for 5 min. 2,3‐Dihydroxysuccinic acid (2.05 equiv.) dissolved in THF (0.3M) (heat was required) was added dropwise to the vigorously stirred biphasic mixture and a white precipitate formed immediately. The reaction was diluted with acetonitrile and filtered. The flask and filter were rinsed with acetonitrile and the filtrate was concentrated in vacuo. The residue was dried under high vacuum affording the trifluoroborate salt as pure product (**22–25**).

**General procedure II** for sp^2^‐sp^2^ Suzuki Miyaura coupling (**10a**–**c, 11a**–**c**):^10^ Cs_2_CO_3_ (3 equiv.), PdCl_2_(dppf) (5 mol‐%) and the trifluoroborate salt (1.6 equiv.) were added in a flask which was evacuated and backfilled thrice with Ar. Bromo‐(–)‐*trans*‐Δ^8^‐tetrahydrocannabinol (**6**)/(**7**) (1 equiv.) was added in dry MeOH (0.1M) and the reaction was stirred at 65 °C. After 16 h the mixture was cooled to r.t. and diluted with Et_2_O. The mixture was filtered through Celite, dried with MgSO_4_, concentrated in vacuo and purified through silica gel column chromatography or preparative HPLC to afford the product (**10a**–**c, 11a**–**c**).

**General procedure III** for sp^2^‐sp^3^ Suzuki Miyaura coupling (**12a**–**12b, 13a**–**13b**):^20^ Bromo‐(–)‐*trans*‐Δ^8^‐tetrahydrocannabinol (**6**)/(**7**) (1 equiv.) was dissolved in toluene (0.2M) and Pd(OAc)_2_ (10 mol‐%), RuPhos (20 mol‐%), alkyl trifluoroborate salt (1.5 equiv.) and aqueous sodium hydroxide (3M, 3 equiv.) were added. The reaction mixture was stirred at 120 °C and followed with TLC until full conversion (± 64 h) after which it was diluted with aqueous hydrochloric acid (1M) and DCM. The mixture was extracted with DCM and the combined organic layers were filtered through Celite, dried with MgSO_4_, concentrated in vacuo and purified through silica gel column chromatography or preparative HPLC to afford the product (**12a**, **12b, 13a**, **13b**).

**3. Experimental details and analysis**

**5‐Propylbenzene‐1,3‐diol** (**1c**):^23^ 1‐Bromo‐3,5‐dimethoxybenzene (400 mg, 1.84 mmol) was dissolved in dry toluene. *n*‐Propylboronic acid (**21**) (243 mg, 2.76 mmol), PdCl_2_(dppf) (5 mol‐%) and potassium phosphate (1.17 g, 5.53 mmol) were added and the flask was evacuated and backfilled with argon thrice. The reaction was stirred at 110 °C for 16 h. The mixture was cooled to r.t. and diluted with Et_2_O after which it was filtered through Celite, dried with MgSO_4_ and concentrated in vacuo, the crude 1,3‐dimethoxy‐5‐propylbenzene was directly used in the next step. The product was dissolved in dry DCM (20 mL) and kept under protective atmosphere. The solution was cooled to 0 °C and boron tribromide (455 µL, 4.79 mmol) was carefully added dropwise. The reaction was left stirring for 16 h and warmed‐up to r.t. The reaction was cooled to 0 °C before saturated aqueous NaHCO_3_ (15 mL) was added. After no more gas evolution was observed NaOH (3M, 5 mL) was added. The mixture was extracted with DCM (2 × 50 mL) and EtOAc (2 × 50 mL) and the resulting aqueous phase was acidified with HCl (1M) until pH 2. The aqueous layer was washed again with DCM (2 × 50 mL) and EtOAc (2 × 50 mL). The combined organic layers were dried with MgSO_4_, concentrated in vacuo and purified by silica gel column chromatography (0→30 % EtOAc in *n*‐heptane) to afford **14** (168 mg, 60 % over two steps) as a green oil. TLC (EtOAc/*n*‐heptane, 3:7 v/v): *R*_f_ = 0.28. ^1^H‐NMR (400 MHz, CDCl_3_) δ 6.25 (d, *J* = 2.2 Hz, 2H), 6.18 (t, *J* = 2.3 Hz, 1H), 4.93 (s, 2H), 2.50–2.41 (m, 2H), 1.66–1.54 (m, 2H), 0.92 (t, *J* = 7.3 Hz, 3H). ^13^C NMR (100 MHz, CDCl_3_) δ 156.66, 146.06, 108.28, 100.34, 38.02, 24.26, 13.93. HRMS (*m/z*): [M + H]^+^ calcd. for C_9_H_12_O_2_: 152.08373, found 152.08270.

**4′‐Hydroxyl‐(–)‐*trans*‐Δ^8^‐tetrahydrocannabinol** (**3**): Benzene‐1,3,5‐triol (**1b**, 9.94 g, 78.8 mmol) was dissolved in dry Et_2_O (200 mL) and stirred vigorously. (*S*)‐*cis*‐verbenol (4.00 g, 26.3 mmol) was added and the reaction was stirred at r.t. Trifluoromethanesulfonic acid (581 µL, 6.60 mmol) was added dropwise at –10 °C and the reaction was left stirring for 4 h. To stop the reaction saturated aqueous NH_4_Cl (100 mL) was added and the mixture was extracted with Et_2_O (2 × 100 mL). The combined organic layers were dried with MgSO_4_, concentrated in vacuo and purified by silica gel column chromatography (0→50 % EtOAc in *n*‐heptane) to give **3** (3.61 g, 53 %) as a yellow solidified oil. TLC (EtOAc/*n*‐heptane, 1:1 v/v): *R*_f_ = 0.60. ^1^H NMR (400 MHz, CDCl_3_) δ 5.94–5.93 (m, 1H), 5.86 (d, *J* = 2.4 Hz, 1H), 5.41 (d, *J* = 4.8 Hz, 1H), 5.17 (bs, 2H), 3.14 (dd, *J* = 15.3, 4.0 Hz, 1H), 2.64 (td, *J* = 10.9, 4.7 Hz, 1H), 2.18–2.09 (m, 1H), 1.86–1.74 (m, 3H), 1.69 (s, 3H), 1.36 (s, 3H), 1.09 (s, 3H). ^13^C NMR (100 MHz, CDCl_3_) δ 156.04, 155.82, 154.98, 134.85, 119.41, 106.45, 97.30, 96.05, 77.36, 45.01, 36.33, 31.45, 27.98, 27.60, 23.62, 18.62. HRMS (*m/z*): [M + H]^+^ calcd. for C_16_H_20_O_3_: 261.14907, found 261.14737.

**4′‐Triflate‐(–)‐*trans*‐Δ^8^‐tetrahydrocannabinol** (**4**): 4′‐Hydroxyl‐(–)‐*trans*‐Δ^8^‐tetrahydrocannabinol (**3**, 100 mg, 384 µmol) was dissolved in dry DCM (4 mL) and stirred at 0 °C before 2,6‐dimethylpyridine (36 µL, 311 µmol) was added. Trifluoromethanesulfonic anhydride (52 µL, 311 µmol) was added over a course of 10 min. After 14 h the reaction was diluted with DCM (10 mL) and washed with water (4 mL), HCl (1M, 4 mL), saturated aqueous NaHCO_3_ (3 mL) and brine (3 mL). The organic layer was dried with MgSO_4_, concentrated in vacuo and purified by silica gel column chromatography (0→25 % EtOAc in *n*‐heptane) to afford **4** (58.9 mg, 56 %, based on recovery of SM) as a yellow oil. TLC (EtOAc/*n*‐heptane, 1:1 v/v): *R*_f_ = 0.79. ^1^H NMR (400 MHz, CDCl_3_) δ 6.37 (d, *J* = 2.5 Hz, 1H), 6.23 (d, *J* = 2.5 Hz, 1H), 5.47–5.41 (m, 1H), 5.21 (s, 1H), 3.20–3.10 (m, 1H), 2.70 (td, *J* = 11.0, 4.8 Hz, 1H), 2.20–2.10 (m, 1H), 1.89–1.76 (m, 3H), 1.71 (s, 3H), 1.39 (s, 3H), 1.10 (s, 3H). ^13^C NMR (100 MHz, CDCl_3_) δ 156.15, 155.84, 148.26, 134.54, 119.43, 113.81, 103.63, 100.81, 78.02, 44.64, 35.68, 31.67, 27.89, 27.49, 23.56, 18.64. ^19^F NMR (377 MHz, CDCl_3_) δ –72.93. HRMS (*m/z*): [M + H]^+^ calcd. for C_17_H_19_F_3_O_5_S: 393.09835, found 393.10073.

**5‐Bromobenzene‐1,3‐diol** (**5**):^24^ 1‐Bromo‐3,5‐dimethoxybenzene (5.00 g, 23.0 mmol) was dissolved in dry DCM (100 mL) and kept under protective atmosphere. The solution was cooled to 0 °C and boron tribromide (7.62 mL, 80.62 mmol) was added carefully dropwise. The reaction was left stirring for 16 h and warmed to r.t. The reaction was cooled to 0 °C before saturated aqueous NaHCO_3_ (70 mL) was added. After no more gas evolution was observed NaOH (1M, 5 mL) was added. The mixture was extracted with DCM (2 × 100 mL) and EtOAc (2 × 100 mL) and the resulting aqueous phase was acidified with HCl (1M) until pH 2. The aqueous layer was extracted again with DCM (3 × 100 mL) and EtOAc (3 × 100 mL). The combined organic layers were dried with MgSO_4_, concentrated in vacuo and purified through silica gel column chromatography (0→30 % EtOAc in *n*‐heptane) to afford **5** (4.35 g, 100 %) as a brown solid. TLC (EtOAc/*n*‐heptane, 3:7 v/v): *R*_f_ = 0.20. ^1^H‐NMR (400 MHz, CDCl_3_) δ 6.59 (d, *J* = 2.2 Hz, 2H), 6.28 (t, *J* = 2.2 Hz, 1H), 5.15 (s, 2H). ^13^C NMR (100 MHz, CDCl_3_) δ 157.76, 122.97, 111.56, 102.21. m.p. 86.9 °C.

**2′‐Bromo‐(–)‐*trans*‐Δ^8^‐tetrahydrocannabinol** (**6**): 5‐Bromobenzene‐1,3‐diol (**5**, 1.35 g, 7.14 mmol) was dissolved in dry DCM (100 mL) and stirred vigorously. (*S*)‐*cis*‐verbenol (1.09 g, 7.14 mmol) was added and the reaction was stirred at r.t. Trifluoromethanesulfonic acid (284 µL, 3.21 mmol) was added dropwise at 0 °C and the reaction was left stirring for 20 h. To stop the reaction saturated aqueous NaHCO_3_ (50 mL) was added and the mixture was extracted with DCM (2 × 100 mL). The combined organic layers were dried with MgSO_4_, concentrated in vacuo and purified through silica gel column chromatography (0→4 % EtOAc in *n*‐heptane) to afford **6** (1.32 g, 57 %) as a yellow oil and **7** as a minor product (199 mg, 9 %). TLC (EtOAc/*n*‐heptane, 1:9 v/v): *R*_f_ = 0.23. ^1^H NMR (400 MHz, CDCl_3_) δ 6.68 (d, *J* = 2.6 Hz, 1H), 6.28 (d, *J* = 2.6 Hz, 1H), 5.46–5.41 (m, 1H), 5.05 (s, 1H), 3.41 (dd, *J* = 16.4, 3.4 Hz, 1H), 2.64 (td, *J* = 10.5, 4.3 Hz, 1H), 2.18–2.11 (m, 1H), 1.86 (m, 3H), 1.71 (s, 3H), 1.37 (s, 3H), 1.07 (s, 3H). ^13^C NMR (100 MHz, CDCl_3_) δ 155.81, 155.01, 134.84, 123.71, 119.64, 118.78, 113.64, 104.40, 77.51, 46.55, 36.80, 35.15, 28.41, 27.41, 23.56, 18.29. HRMS (*m/z*): [M + H]^+^ calcd. for C_16_H_19_BrO_2_: 323.06467, found 323.06511. **4′‐Bromo‐(–)‐*trans*‐Δ^8^‐tetrahydrocannabinol** (**7**): TLC (EtOAc/*n*‐heptane, 1:9 v/v): *R*_f_ = 0.35. ^1^H NMR (400 MHz, CDCl_3_) δ 6.61 (d, *J* = 1.9 Hz, 1H), 6.43 (d, *J* = 1.9 Hz, 1H), 5.46–5.40 (m, 1H), 5.24 (s, 1H), 3.16 (dd, *J* = 15.7, 4.4 Hz, 1H), 2.66 (td, *J* = 11.1, 4.8 Hz, 1H), 2.19–2.09 (m, 1H), 1.84–1.74 (m, 3H), 1.70 (s, 3H), 1.38 (s, 3H), 1.09 (s, 3H). ^13^C NMR (100 MHz, CDCl_3_) δ 155.94, 155.79, 134.67, 119.77, 119.43, 113.82, 112.74, 110.84, 77.62, 44.81, 35.81, 31.70, 27.93, 27.53, 23.58, 18.58. HRMS (*m/z*): [M + H]^+^ calcd. for C_16_H_19_BrO_2_: 323.06467, found 323.06620.

**5‐Chlorobenzene1,3‐diol** (**8**): 1‐Chloro‐3,5‐dimethoxybenzene (1.01 g, 5.85 mmol) was dissolved in ACN (12 mL) and kept under protective atmosphere. Iodotrimethylsilane (4.78 mL, 35.11 mmol) was added and the solution was heated to 70 °C. The reaction was left stirring overnight at reflux. The mixture was cooled to r.t. and concentrated in vacuo. The residue was dissolved in 1M aqueous HCl (10 mL) and DCM (15 mL), after which the aqueous layer was extracted with DCM (2 × 15 mL). The combined organic layers were dried with Na_2_SO_4_, concentrated in vacuo and purified using silica gel column chromatography (0→20 % EtOAc in *n*‐heptane) to afford **8** (222 mg, 26 %) as a yellow solidified oil. TLC (EtOAc/*n*‐heptane, 1:4 v/v): *R*_f_ = 0.20 ^1^H‐NMR (500 MHz, CDCl_3_) δ 6.44 (d, *J* = 2.2 Hz, 2H), 6.24 (s, 1H), 5.01 (s, 2H); ^13^C NMR (126 MHz, CDCl_3_) δ 157.51, 135.23, 108.46, 101.52. m.p. 58.8 °C.

**5‐Iodobenzene‐1,3‐diol** (**9**): 3,5‐Dimethoxyiodobenzene (**15**, 0.867 g, 3.28 mmol) was dissolved in ACN (7 mL) and kept under protective atmosphere. Iodotrimethylsilane (2.80 mL, 19.7 mmol) was added and the solution was heated to 70 °C. The reaction was left stirring overnight at reflux. After cooling to r.t. the reaction mixture was concentrated in vacuo and the residue was dissolved in 1 M aqueous HCl (10 mL) and DCM (15 mL). The aqueous layer was extracted with DCM (2 × 15 mL), the combined organic layers were dried with Na_2_SO_4_, concentrated in vacuo and purified with silica gel column chromatography (0→20 % EtOAc in *n*‐heptane) to afford **9** (198 mg, 26 %, based on recovery of SM) as a brown solid. TLC (EtOAc/*n*‐heptane, 1:1 v/v): *R*_f_ = 0.50; ^1^H NMR (400 MHz, CDCl_3_) δ 6.79 (d, *J* = 2.2 Hz, 2H), 6.31 (t, *J* = 2.2 Hz, 1H), 5.22 (s, 2H). ^13^C NMR (100 MHz, CDCl_3_) δ 157.42, 117.59, 103.01, 93.80. m.p. 74.1 °C.

**2′‐*S*tyrene(–)‐*trans*‐Δ^8^‐Tetrahydrocannabinol** (**10a**): Synthesized according to general procedure II from 2′‐bromo‐(–)‐*trans*‐Δ^8^‐tetrahydrocannabinol (**6**, 88.3 mg, 258 µmol) and potassium (E)‐styryl trifluoroborate (86.6 mg, 412 µmol) which afforded (**10a**, 69.2 mg, 78 %) as a colorless oil. TLC (EtOAc/*n*‐heptane, 1:9 v/v): *R*_f_ = 0.11. ^1^H NMR (500 MHz, CDCl_3_) δ 7.51–7.47 (m, 2H), 7.37 (t, *J* = 7.7 Hz, 2H), 7.29–7.26 (m, 1H), 7.19 (d, *J* = 16.0 Hz, 1H), 6.93 (d, *J* = 16.0 Hz, 1H), 6.66 (d, *J* = 2.6 Hz, 1H), 6.28 (d, *J* = 2.6 Hz, 1H), 5.45 (m, 1H), 4.79 (bs, 1H), 2.83 (td, *J* = 10.8, 4.5 Hz, 1H), 2.71–2.64 (m, 1H), 2.23–2.15 (m, 1H), 1.89–1.80 (m, 2H), 1.62 (s, 3H), 1.40 (s, 3H), 1.14 (s, 3H). ^13^C NMR (126 MHz, CDCl_3_) δ 155.14, 154.84, 138.54, 137.62, 134.88, 128.97, 128.90, 128.24, 127.77, 126.65, 119.99, 117.32, 106.33, 103.98, 76.73, 46.01, 39.69, 33.10, 28.42, 27.59, 23.74, 18.38. HRMS (*m/z*): [M + H]^+^ calcd. for C_24_H_26_O_2_: 347.20110, found 347.20075.

**2′‐Naphthalene(–)‐*trans*‐Δ^8^‐tetrahydrocannabinol** (**10b‐S*_a_*** and **10b‐R*_a_***): Synthesized according to general procedure II from 2′‐bromo‐(–)‐*trans*‐Δ^8^‐tetrahydrocannabinol (**6**) (150 mg, 464 µmol) and potassium (1‐naphthalene) trifluoroborate (**24**, 174 mg, 743 µmol) and purified using preparative HPLC to afford (**10b**, 28.5 mg, 17 %) as a colorless oil. The product was obtained as an inseparable mixture of two atropisomers **10b‐R_a_** and **10b‐S_a_** in ratios of 0.64:1.00, respectively. TLC (EtOAc/*n*‐heptane, 1:9 v/v): *R*_f_ = 0.15. **10b‐*R*_a_:** ^1^H NMR (500 MHz, [D_6_]DMSO) δ 7.94 (d, *J* = 8.2 Hz, 1H), 7.81 (d, *J* = 7.9 Hz, 1H), 7.56 (dd, i = 6.8, 1.6 Hz, 1H), 7.56–7.52 (m, 1H), 7.45–7.43 (m, 1H), 7.41–7.39 (m, 1H), 7.32 (dd, *J* = 7.1, 1.2 Hz, 1H), 6.23 (d, *J* = 2.6 Hz, 1H), 6.16 (d, *J* = 2.6 Hz, 1H), 5.18 (d, *J* = 2.7 Hz, 1H), 2.69 (dt, *J* = 11.2, 5.6 Hz, 1H), 2.04 (m, 1H), 1.77–1.67 (m, 1H), 1.58 (dd, *J* = 11.6, 4.4 Hz, 1H), 1.33 (s, 3H), 1.19 (s, 3H), 1.17 (s, 1H), 0.93 (d, *J* = 12.6 Hz, 1H), 0.92–0.86 (bs, 3H). ^13^C NMR (126 MHz, [D_6_]DMSO) δ 156.09, 155.36, 140.94, 140.22, 133.93, 133.30, 132.06, 128.49, 127.63, 126.62, 126.36, 126.29, 125.92, 125.85, 119.94, 115.78, 111.97, 103.51, 76.41, 45.15, 36.65, 32.61, 27.75, 27.56, 23.14, 18.86. **10b‐*S*_a_**: ^1^H NMR (500 MHz, [D_6_]DMSO) δ 8.00–7.98 (m, 2H), 7.60 (dd, *J* = 8.3, 7.0 Hz, 1H), 7.51 (d, *J* = 1.5 Hz, 1H), 7.45 (m, 2H), 7.41–7.39 (m, 1H), 6.27 (d, *J* = 2.6 Hz, 1H), 6.25 (d, *J* = 2.6 Hz, 1H), 5.08 (d, *J* = 4.3 Hz, 1H), 2.16 (td, *J* = 10.7, 4.9 Hz, 1H), 1.97–1.93 (m, 1H), 1.55–1.53 (m, 2H), 1.32 (s, 3H), 1.31–1.29 (m, 1H), 1.18 (s, 3H), 1.06 (s, 3H), 0.97–0.90 (m, 1H). ^13^C NMR (126 MHz, [D_6_]DMSO) δ 156.85, 154.69, 141.46, 141.14, 133.30, 133.26, 130.55, 128.83, 127.89, 126.93, 126.67, 126.45, 126.09, 125.41, 119.68, 115.84, 111.66, 103.65, 76.35, 45.01, 37.58, 33.44, 27.75, 27.56, 23.31, 18.90. HRMS (*m/z*): [M + H]^+^ calcd. for C_26_H_26_O_2_: 371.20110, found 371.20214.

**2′‐(4‐Methoxybenzene)‐(–)‐*trans*‐Δ^8^‐tetrahydrocannabinol** (**10c**): Synthesized according to general procedure I from 2′‐bromo‐(–)‐*trans*‐Δ^8^‐tetrahydrocannabinol (**6**, 98.0 mg, 68 µmol) and potassium (4‐methoxyphenyl) trifluoroborate (**25**, 104 mg, 485 µmol) to afford **10c** (24.0 mg, 23 %) as a colorless oil. **TLC** (EtOAc/*n*‐heptane, 1:9 v/v): *R*_f_ = 0.07. ^1^H NMR (400 MHz, CDCl_3_) δ 7.25 (d, *J* = 8.7 Hz, 2H), 6.92 (d, *J* = 8.7 Hz, 2H), 6.29 (d, *J* = 0.6 Hz), 5.28 (m, 1H), 4.72 (s, 1H), 3.85 (s, 3H), 2.86 (td, *J* = 10.9, 4.8 Hz, 1H), 2.13–2.06 (m, 1H), 1.76–1.69 (m, 2H), 1.58–1.52 (m, 1H), 1.45–1.40 (m, 1H), 1.38 (s, 3H), 1.35–1.33 (m, 3H), 1.22 (s, 3H). ^13^C NMR (126 MHz, CDCl_3_) δ 158.70, 155.14, 154.35, 143.56, 135.50, 134.66, 129.10, 118.93, 116.34, 113.89, 110.62, 102.94, 76.41, 55.37, 45.11, 36.70, 32.68, 27.99, 27.50, 23.29, 18.34. HRMS (*m/z*): [M + H]^+^ calcd. for C_23_H_26_O_3_: 351.19602, found 351.19571.

**4′‐*S*tyrene‐(–)‐*trans*‐Δ^8^‐tetrahydrocannabinol** (**11a**): Synthesized according to general procedure II from 4′‐bromo‐(–)‐*trans*‐Δ^8^‐tetrahydrocannabinol (**7**, 20.0 mg, 62 µmol) and potassium (*E*)‐styryl trifluoroborate (21.0 mg, 99 µmol) to afford **11a** (4.8 mg, 27 %) as a colorless oil. TLC (EtOAc/*n*‐heptane, 1:9 v/v): *R*_f_ = 0.27. ^1^H NMR (500 MHz, CDCl_3_) δ 7.49–7.45 (m, 2H), 7.34 (t, *J* = 7.7 Hz, 2H), 7.24 (m 1H), 7.02 (d, *J* = 16.3 Hz, 1H), 6.92 (d, *J* = 16.3 Hz, 1H), 6.62 (d, *J* = 1.6 Hz, 1H), 6.44 (d, *J* = 1.7 Hz, 1H3′), 5.45–5.43 (m, 1H), 4.81 (s, 1H), 3.21 (dd, *J* = 15.9, 4.5 Hz, 1H), 2.74 (td, *J* = 10.8, 4.7 Hz, 1H), 2.20–2.12 (m, 1H), 1.92–1.78 (m, 3H), 1.72 (s, 3H), 1.40 (s, 3H), 1.13 (s, 3H). ^13^C NMR (126 MHz, CDCl_3_) δ 155.48, 155.31, 137.44, 137.04, 134.81, 128.80, 128.77, 128.21, 127.71, 126.64, 119.51, 113.30, 108.77, 105.63, 77.06, 44.99, 36.08, 31.98, 28.03, 27.71, 23.65, 18.68. HRMS (*m/z*): [M + H]^+^ calcd. for C_24_H_26_O_2_: 347.20110, found 347.20105.

**4′‐Naphthalene‐(–)‐*trans*‐Δ^8^‐tetrahydrocannabinol** (**11b**): Synthesized according to general procedure II from 4′‐bromo‐(–)‐*trans*‐Δ^8^‐tetrahydrocannabinol (**7**, 25.0 mg, 77 µmol) and potassium (1‐naphthalene) trifluoroborate (**24**, 29.0 mg, 120 µmol) to afford **11b** (17.1 mg, 60 %) as a colorless oil. TLC (EtOAc/*n*‐heptane, 1:9 v/v): *R*_f_ = 0.27. ^1^H NMR (500 MHz, CDCl_3_) δ 8.04 (d, *J* = 8.4 Hz, 1H), 7.87 (d, *J* = 8.0 Hz, 1H), 7.82 (d, *J* = 8.1 Hz, 1H), 7.51–7.44 (m, 2H), 7.44–7.39 (m, 2H), 6.59 (d, *J* = 1.7 Hz, 1H), 6.41 (d, *J* = 1.7 Hz, 1H), 5.49–5.47 (m, 1H), 4.98 (s, 1H), 3.30 (dd, *J* = 17.2, 4.7 Hz, 1H), 2.83 (td, *J* = 10.8, 4.8 Hz, 1H), 2.24–2.16 (m, 1H), 2.01–1.84 (m, 3H), 1.74 (s, 3H), 1.42 (s, 3H), 1.19 (s, 3H). ^13^C NMR (126 MHz, CDCl_3_) δ 155.15, 154.85, 140.27, 139.85, 134.92, 133.90, 131.60, 128.29, 127.65, 126.69, 126.42, 126.01, 125.83, 125.44, 119.52, 112.43, 112.33, 109.44, 77.08, 45.10, 36.13, 31.95, 28.10, 27.75, 23.67, 18.76. HRMS (*m/z*): [M + H]^+^ calcd. for C_26_H_26_O_2_: 371.20110, found 371.20176.

**4′‐(4‐Methoxybenzene)‐(–)‐*trans*‐Δ^8^‐tetrahydrocannabinol** (**11c**): Synthesized according to general procedure II from 4′‐bromo‐(–)‐*trans*‐Δ^8^‐tetrahydrocannabinol (**7**, 100 mg, 309 µmol) and potassium (4‐methoxyphenyl) trifluoroborate (**25**, 106 mg, 495 µmol) to afford **11c** (31.0 mg, 29 %) as a colorless oil. TLC (EtOAc/*n*‐heptane, 1:9 v/v): *R*_f_ = 0.17. ^1^H NMR (500 MHz, CDCl_3_) 7.47 (d, *J* = 8.8 Hz, 2H), 6.93 (d, *J* = 8.8 Hz, 2H), 6.65 (d, *J* = 1.7 Hz, 1H), 6.48 (d, *J* = 1.8 Hz, 1H), 5.45 (d, *J* = 5.4 Hz, 1H), 4.87 (s, 1H), 3.83 (s, 3H), 3.23 (dd, *J* = 16.4, 4.5 Hz, 1H), 2.76 (td, *J* = 10.8, 4.6 Hz, 1H), 2.21–2.09 (m, 1H), 1.89–1.80 (m, 3H), 1.72 (s, 3H), 1.41 (s, 3H), 1.14 (s, 3H). ^13^C NMR (126 MHz, CDCl_3_) δ 158.89, 155.55, 155.39, 143.91, 135.73, 134.85, 127.91, 119.51, 114.20, 112.61, 108.76, 105.89, 77.06, 55.46, 45.03, 36.12, 31.81, 28.05, 27.73, 23.65, 18.72. HRMS (*m/z*): [M + H]^+^ calcd. for C_23_H_26_O_3_: 351.19602, found 351.19740.

***Ortho*‐*n*‐pentyl‐(–)‐*trans*‐Δ^8^‐tetrahydrocannabinol** (**12a**): Synthesized according to general procedure III from 2′‐bromo‐(–)‐*trans*‐Δ^8^‐tetrahydrocannabinol (**6**, 77.9 mg, 241 µmol) and potassium *n*‐pentylboron trifluoride (**23**, 64.4 mg, 362 µmol). Silica gel column chromatography (0→8 % EtOAc/*n*‐heptane) afforded **12a** (38.8 mg, 51 %) as an inseparable mixture with **6**. TLC (EtOAc/*n*‐heptane, 1:9 v/v): *R*_f_ = 0.23. ^1^H NMR (500 MHz, CDCl_3_) δ 6.29 (d, *J* = 2.7 Hz, 1H), 6.16 (d, *J* = 2.6 Hz, 1H), 5.47–5.44 (m, 1H), 4.88 (s, 1H), 2.71–2.62 (m, 1H), 2.60–2.55 (m, 3H), 2.21–2.10 (m, 1H), 1.87–1.81 (m, 3H), 1.70 (s, 3H), 1.67–1.57 (m, 2H), 1.36 (s, 3H), 1.35–1.23 (m, 4H), 1.06 (s, 3H), 0.94–0.86 (m, 3H). ^13^C NMR (126 MHz, CDCl_3_) δ 154.94, 154.55, 143.99, 134.64, 120.13, 117.21, 109.24, 102.19, 76.35, 46.63, 38.86, 33.53, 33.47, 32.07, 31.07, 28.49, 27.59, 23.61, 22.67, 18.25, 14.22. HRMS (*m/z*): [M + H]^+^ calcd. for C_21_H_30_O_2_: 315.23240, found 315.23200.

**2′‐*Ortho*‐*n*‐propyl‐(–)‐*trans*‐Δ^8^‐tetrahydrocannabinol** (**12b**): Synthesized according to general procedure III from 2′‐bromo‐(–)‐*trans*‐Δ^8^‐tetrahydrocannabinol (**6**, 130 mg, 402 µmol) and potassium *n*‐propylboron trifluoride (**22**, 90.5 mg, 603 µmol). Silica gel column chromatography (0→8 % EtOAc/*n*‐heptane) afforded **12b** (63.9 mg, 56 %) as an inseparable mixture with **6**. TLC (Toluene): *R*_f_ = 0.05. ^1^H NMR (500 MHz, CDCl_3_) δ 6.29 (d, *J* = 2.8Hz, 1H), 6.16 (d, *J* = 2.7 Hz, 1H), 5.48–5.43 (m, 1H), 4.97–4.89 (m, 1H), 2.71–2.62 (m, 1H), 2.60 (m, 1H), 2.56 (t, *J* = 7.9 Hz, 2H), 2.18–2.10 (m, 1H), 1.88–1.79 (m, 3H), 1.70 (s, 3H), 1.65–1.59 (m, 2H), 1.36 (s, 3H), 1.06 (s, 3H), 0.96 (t, *J* = 7.3 Hz, 3H). ^13^C NMR (126 MHz, CDCl_3_) δ 154.94, 154.56, 143.78, 134.65, 120.15, 117.28, 109.22, 102.24, 76.35, 46.65, 38.88, 35.62, 33.48, 28.50, 27.59, 24.54, 23.64, 18.25, 14.29. HRMS (*m/z*): [M + H]^+^ calcd. for C_19_H_26_O_2_: 287.20110, found 287.20130.

**(–)‐*trans*‐Δ^8^‐tetrahydrocannabinol** (**13a**):^9^ 5‐Pentylbenzene‐1,3‐diol (**1a**, 1.18 g, 6.60 mmol) and (*S*)‐*cis*‐verbenol (1.00 g, 6.60 mmol) were stirred at r.t. in dry DCM (70 mL). Trifluoromethanesulfonic acid (145 µL, 1.64 mmol) was added dropwise at 0 °C and the reaction was left stirring for 2 h. To stop the reaction saturated aqueous NaHCO_3_ (70 mL) was added and the mixture was extracted with DCM (2 × 70 mL). The combined organic layers were dried with MgSO_4_, concentrated in vacuo and purified through silica gel column chromatography (0→4 % EtOAc/*n*‐heptane) to afford **1** (691 mg, 33 %) as a yellow oil. TLC (EtOAc/*n*‐heptane, 1:9 v/v): *R*_f_ = 0.38. ^1^H NMR (400 MHz, CDCl_3_) δ 6.29 (d, *J* = 1.5 Hz, 1H), 6.11 (d, *J* = 1.5 Hz, 1H), 5.44 (d, *J* = 4.8 Hz, 1H), 4.93 (s, 1H) 3.22 (dd, *J* = 15.8, 4.2 Hz, 1H), 2.71 (td, *J* = 10.8, 4.6 Hz, 1H), 2.44 (td, *J* = 7.4, 2.0 Hz, 2H), 2.20–2.11 (m, 1H), 1.91–1.76 (m, 3H), 1.71 (s, 3H), 1.62–1.53 (m, 2H), 1.39 (s, 3H), 1.34–1.26 (m, 4H), 1.12 (s, 3H), 0.91–0.87 (m, 3H). ^13^C NMR (100 MHz, CDCl_3_) δ 154.93, 154.92, 142.83, 134.89, 119.45, 110.70, 110.20, 107.83, 76.86, 45.05, 36.17, 35.59, 31.74, 31.72, 30.74, 28.04, 27.70, 23.63, 22.69, 18.63, 14.16. HRMS (*m/z*): [M + H]^+^ calcd. for C_21_H_30_O_2_: 315.23240, found 315.23343.

**(–)‐*trans*‐Δ^8^‐tetrahydrocannabinol** (**13a**): Synthesized according to general procedure III from 4′‐bromo‐(–)‐*trans*‐Δ^8^‐tetrahydrocannabinol (**7**, 52.1 mg, 161 µmol) and potassium *n*‐pentylboron trifluoride (**23**, 43.0 mg, 242 µmol) to afford **13a** (17.4 mg, 34 %) as a yellow oil. Spectral data were in agreement with previously synthesized **1** and hence no further purification was executed.

**4′‐Propyl‐(–)‐*trans*‐Δ^8^‐tetrahydrocannabinol** (**13b**): 5‐Propylbenzene‐1,3‐diol (**1c**, 150 mg, 986 µmol) and (*S*)‐*cis*‐verbenol (150 g, 986 µmol) were stirred at r.t. in dry DCM (20 mL). Trifluoromethanesulfonic acid (26.2 µL, 296 µmol) was added dropwise at 0 °C and the reaction was left stirring for 3 h. To stop the reaction saturated aqueous NaHCO_3_ (20 mL) was added and the mixture was extracted with DCM (2 × 40 mL). The combined organic layers were dried with MgSO_4_, concentrated in vacuo and purified through silica gel column chromatography (0→4 % EtOAc/*n*‐heptane) to afford **2** (55.9 mg, 20 %) as a yellow oil. TLC (EtOAc/*n*‐heptane, 1:9 v/v): *R*_f_ = 0.29. ^1^H NMR (400 MHz, CDCl_3_) δ 6.28 (d, *J* = 1.7 Hz, 1H), 6.10 (d, *J* = 1.6 Hz, 1H), 5.45–5.41 (m, 1H), 4.82 (s, 1H), 3.25–3.15 (m, 1H), 2.71 (td, *J* = 10.8, 4.6 Hz, 1H), 2.42 (td, *J* = 7.4, 2.4 Hz, 2H), 2.19–2.10 (m, 1H), 1.91–1.77 (m, 3H), 1.71 (s, 3H), 1.59 (t, *J* = 7.4 Hz, 2H), 1.38 (s, 3H), 1.11 (s, 3H), 0.92 (t, *J* = 7.3 Hz, 3H). ^13^C NMR (100 MHz, CDCl_3_) δ 154.94, 154.89, 142.58, 134.89, 119.46, 110.72, 110.31, 107.85, 76.83, 45.04, 37.71, 36.17, 31.73, 28.04, 27.71, 24.12, 23.63, 18.64, 14.07. HRMS (*m/z*): [M + H]^+^ calcd. for C_19_H_26_O_2_: 287.20110, found 287.20004.

**Propyl‐(–)‐*trans*‐Δ^8^‐tetrahydrocannabinol** (**13b**): Synthesized according to general procedure III from 4′‐bromo‐(–)‐*trans*‐Δ^8^‐tetrahydrocannabinol (**7**, 38.2 mg, 118 µmol) and potassium *n*‐propylboron trifluoride (**22**, 26.6 mg, 177 µmol) to afford **13b** (11.3 mg, 33 %) as a yellow oil. Spectral data were in agreement with previously synthesized **2** and hence no further purification was executed.

**3,5‐Dimethoxyiodobenzene** (**15**): 1‐Bromo‐3,5‐dimethoxybenzene (1.09 g, 5.00 mmol) was dissolved in THF (2.5 mL) and kept under protective atmosphere. Magnesium turnings (133 mg, 5.50 mmol) were added and the mixture was stirred vigorously. One drop of 1,2‐dibromoethane (±45 mg, 250 µmol) was added, and a reflux condenser was placed on top of the flask. The reaction was then heated to reflux temperature and allowed to stir for 2 h. After this time, the reaction mixture was cooled on ice, and iodine (845 mg, 3.33 mmol) in THF (2.5 mL) was added. The reaction was allowed to stir for 2 h at 0 °C. After this time, 1M aqueous HCl (10 mL) was added slowly and the mixture was extracted with Et_2_O (3 × 10 mL). The combined organic layers were washed with 1M aqueous Na_2_S_2_O_3_ (3 × 10 mL), concentrated in vacuo and purified through silica gel column chromatography (0→10 % EtOAc in *n*‐heptane) to afford **15** (1.00 g, 76 %) as a brown solidified oil. TLC (EtOAc/*n*‐heptane, 1:9 v/v): *R*_f_ = 0.45. ^1^H‐NMR (500 MHz, CDCl_3_) δ 6.86 (d, *J* = 2.2 Hz, 2H), 6.40 (t, *J* = 2.2 Hz, 1H), 3.76 (s, 6H). ^13^C NMR (100 MHz, CDCl_3_) δ 161.07, 115.81, 100.67, 94.05, 55.49. m.p. 72.4 °C.

**Chloro(–)‐*trans*‐Δ^8^‐tetrahydrocannabinol** (**16** and **17**): 5‐Chlorobenzene1,3‐diol (**8**, 107 mg, 740 µmol) was dissolved in dry DCM (5 mL) and stirred vigorously. (*S*)‐*cis*‐verbenol (113 mg, 740 µmol) was added and the reaction was stirred at r.t. Trifluoromethanesulfonic acid (29 µL, 333 µmol) was added dropwise at 0 °C and the reaction was left stirring for 20 h. To stop the reaction saturated aqueous NaHCO_3_ (50 mL) was added and the mixture was extracted with DCM (2 × 100 mL). The combined organic layers were dried with MgSO_4_, concentrated in vacuo and purified through silica gel column chromatography (0→4 % EtOAc/*n*‐heptane) to afford **16** (53.3 mg, 26 %) as a yellow oil and **17** as a minor product (13.7 mg, 7 %). **2′‐Chloro(–)‐*trans*‐Δ^8^‐tetrahydrocannabinol** (**16**): TLC (EtOAc/*n*‐heptane, 1:5 v/v): *R*_f_ = 0.40 ^1^H NMR (500 MHz, CDCl_3_) δ 6.40 (d, *J* = 2.6 Hz, 1H), 6.17 (d, *J* = 2.6 Hz, 1H), 5.39 (s, 1H), 5.36 (d, *J* = 4.1 Hz, 1H), 3.22 (dd, *J* = 16.4, 4.4 Hz, 1H), 2.61 (td, *J* = 10.8, 4.5 Hz, 1H), 2.12–2.02 (m, 1H), 1.77–1.73 (m, 2H), 1.71–1.66 (m, 1H), 1.63 (s, 3H), 1.30 (s, 3H), 0.99 (s, 3H); ^13^C NMR (126 MHz, CDCl_3_) δ 155.73, 154.79, 134.68, 134.59, 119.43, 116.99, 110.19, 103.59, 77.39, 45.97, 36.32, 33.57, 28.15, 27.33, 23.44, 18.24; HRMS (*m/z*): [M + H]^+^ calcd. for C_16_H_19_ClO_2_: 279.11518, found 279.11664. **4′‐Chloro(–)‐*trans*‐Δ^8^‐tetrahydrocannabinol** (**17**): TLC (EtOAc/*n*‐heptane, 1:5 v/v): *R*_f_ = 0.47. ^1^H NMR (500 MHz, CDCl_3_) δ 6.45 (d, *J* = 2.1 Hz, 1H), 6.29 (d, *J* = 2.0 Hz, 1H), 5.43 (d, *J* = 3.7 Hz, 1H), 5.03 (s, 1H), 3.15 (dd, *J* = 15.7, 4.8 Hz, 1H), 2.67 (td, *J* = 11.0, 4.8 Hz, 1H), 2.16–2.10 (m, 1H), 1.85–1.76 (m, 3H), 1.70 (s, 3H), 1.37 (s, 3H), 1.09 (s, 3H); ^13^C NMR (126 MHz, CDCl_3_) δ 155.72, 155.47, 134.53, 132.14, 119.31, 112.02, 110.79, 107.80, 77.35, 44.68, 35.77, 31.50, 27.80, 27.42, 23.45, 18.45; HRMS (*m/z*): [M + Na]^+^ calcd. for C_16_H_19_O_2_Cl: 278.10736, found 278.10653.

**Iodo(–)‐*trans*‐Δ^8^‐tetrahydrocannabinol** (**18** and **19**): 5‐Iodobenzene‐1,3‐diol (**9**, 110 mg, 466 µmol) was dissolved in dry DCM (5 mL) and stirred vigorously. (*S*)‐*cis*‐verbenol (71.0 mg, 466 µmol) was added and the reaction was stirred at r.t. Trifluoromethanesulfonic acid (18.6 µL, 210 µmol) was added dropwise at 0 °C and the reaction was left stirring for 20 h. To stop the reaction saturated aqueous NaHCO_3_ (50 mL) was added and the mixture was extracted with DCM (2 × 100 mL). The combined organic layers were dried with MgSO_4_, concentrated in vacuo and purified through silica gel column chromatography (0→4 % EtOAc/*n*‐heptane) to afford **18** (43.7 mg, 25 %) as a yellow oil and **19** as a minor product (11.1 mg, 6 %). **2′‐Iodo(–)‐*trans*‐Δ^8^‐tetrahydrocannabinol** (**18**): TLC (EtOAc/*n*‐heptane, 1:1 v/v): *R*_f_ = 0.35. ^1^H NMR (500 MHz, CDCl_3_) δ 7.00 (d, *J* = 2.6 Hz, 1H), 6.31 (d, *J* = 2.6 Hz, 1H), 5.44 (d, *J* = 3.7 Hz, 1H), 5.25 (s, 1H), 3.48 (dd, *J* = 17.2, 3.6 Hz, 1H), 2.52 (td, *J* = 10.7, 4.3 Hz, 1H), 2.19–2.13 (m, 1H), 1.93–1.82 (m, 2H), 1.72 (s, 3H), 1.69–1.66 (m, 1H), 1.36 (s, 3H), 1.05 (s, 3H); ^13^C NMR (126 MHz, CDCl_3_) δ 155.09, 154.57, 134.64, 121.41, 120.46, 119.70, 105.30, 96.81, 77.28, 46.96, 37.45, 37.19, 28.40, 27.22, 23.41, 18.09. HRMS (*m/z*): [M + H]^+^ calcd. for C_16_H_19_IO_2_: 371.05080, found 371.05235. **4′‐Iodo(–)‐*trans*‐Δ^8^‐tetrahydrocannabinol** (**19**): TLC (EtOAc/*n*‐heptane, 1:1 v/v): *R*_f_ = 0.44. ^1^H NMR (500 MHz, CDCl_3_) δ 6.80 (d, *J* = 1.7 Hz, 1H), 6.62 (d, *J* = 1.7 Hz, 1H), 5.42 (d, *J* = 4.0 Hz, 1H), 4.93 (s, 1H), 3.15 (dd, *J* = 15.3, 4.4 Hz, 1H), 2.66 (td, *J* = 11.0, 4.7 Hz, 1H), 2.16–2.10 (m, 1H), 1.83–1.73 (m, 3H), 1.69 (s, 3H), 1.36 (s, 3H), 1.08 (s, 3H); ^13^C NMR (126 MHz, CDCl_3_) δ 155.80, 155.53, 134.53, 119.80, 119.30, 116.35, 113.37, 90.32, 77.31, 44.65, 35.66, 31.61, 27.80, 27.43, 23.45, 18.47; HRMS (*m/z*): [M + Na]^+^ calcd. for C_16_H_19_O_2_I: 370.04297, found 370.04265.

***n*‐Propylboronic acid** (**20**): 1‐Bromopropane (2.15 mL, 23.6 mmol) and dry THF (12 mL) were combined and cooled to 0 °C. Magnesium turnings (631 mg, 25.9 mmol) and one drop of 1,2‐dibromoethane were added. After 15 min the cooling bath was removed and the reaction was refluxed for 2 h at 75 °C after which it was cooled to r.t. Trimethylborate (2.89 mL, 25.9 mmol) was dissolved in Et_2_O (100 mL), stirred vigorously and cooled to –78 °C. Freshly prepared propylmagnesium bromide was added dropwise to the mixture. The reaction was left stirring for 2 h at –78 °C after it was warmed up to r.t. 10 % aqueous HCl (80 mL) was added slowly and the biphasic reaction mixture was left stirring for 15 min. The layers were separated and the aqueous layer was washed with Et_2_O (2 × 80 mL). The combined organic layers were dried with MgSO_4_, concentrated in vacuo and the crude product was recrystallized by dissolving in hot water (20 mL) and cooling to 0 °C. The product was isolated by filtration and the flask and filter were rinsed with *n*‐heptane (4 mL). The filtered solid was dried under high vacuum to afford **20** (539 mg, 26 % over two steps) as white crystals. ^1^H NMR (400 MHz, (CD_3_)_2_SO) δ 7.33 (s, 2H), 1.39–1.28 (m, 3H), 0.85 (t, *J* = 7.3 Hz, 2H), 0.57 (t, *J* = 7.7 Hz, 3H). ^13^C NMR (100 MHz, CDCl_3_) δ 17.54, 17.09 (CH_2_ next to B not visible; quadrupolar relaxation). ^11^B NMR (128 MHz, CDCl_3_) δ 32.18 (s). m.p. 101.1 °C.

***n*‐Pentylboronic acid** (**21**): Trimethylborate (1.1 mL, 10.0 mmol) was dissolved in Et_2_O (60 mL), stirred vigorously and cooled to –78 °C. Pentylmagnesium bromide (7.69 mL, 10.0 mmol, 1.3M in THF) was added dropwise. The reaction was left stirring for 2 h at –78 °C after it was warmed up to r.t. 10 % aqueous HCl (40 mL) was added slowly and the biphasic reaction mixture was left stirring for 15 min. The layers were separated and the aqueous layer was washed with Et_2_O (2 × 40 mL). The combined organic layers were dried with MgSO_4_, concentrated in vacuo and the crude product was recrystallized by dissolving in hot water (10 mL) and cooling to 0 °C. The product was isolated by filtration and the flask and filter were rinsed with *n*‐heptane (2 mL). The filtered solid was dried under high vacuum to afford **21** (774 mg, 67 %) as white crystals. ^1^H NMR (400 MHz, CDCl_3_) δ 1.49–1.36 (m, 2H), 1.34–1.27 (m, 4H), 0.95–0.84 (m, 4H), 0.84–0.75 (m, 1H). ^13^C NMR (100 MHz, CDCl_3_) δ 34.68, 28.20, 23.47, 22.65, 14.15. ^11^B NMR (128 MHz, CDCl_3_) δ 33.32 (s). m.p. 88.2 °C.

**Potassium *n*‐propylboron trifluoride** (**22**): Synthesized according to general procedure I from *n*‐propylboronic acid (**20**, 200 mg, 2.28 mmol), potassium fluoride (529 mg, 9.10 mmol) and 2,3‐dihydroxysuccinic acid (700 mg, 4.66 mmol) to afford **22** (257 mg, 75 %) as white crystals. ^1^H NMR (400 MHz, (CD_3_)_2_SO) δ 1.22–1.06 (m, 2H), 0.83–0.76 (m, 3H), –0.01 to –0.10 (m, 2H). ^13^C NMR (100 MHz, (CD_3_)_2_SO) δ 18.74, 18.23 (CH_2_ next to B not visible; quadrupolar relaxation). ^11^B NMR (128 MHz, (CD_3_)_2_SO) δ 4.76 (d,* J* = 64.8 Hz). ^19^F NMR (377 MHz (CD_3_)_2_SO) δ –136.49 to –136.98 (m). m.p. 378.9 °C.

**Potassium *n*‐pentylboron trifluoride** (**23**): Synthesized according to general procedure I from *n*‐pentylboronic acid (**21**, 600 mg, 5.17 mmol), potassium fluoride (1.20 g, 20.7 mmol) and 2,3‐dihydroxysuccinic acid (1.59 g, 10.6 mmol) to afford **23** (872 mg, 95 %) as white crystals. ^1^H NMR (400 MHz, (CD_3_)_2_SO) δ 1.26–1.07 (m, 6H), 0.82 (t, *J* = 7.1 Hz, 3H), –0.03 to –0.14 (m, 2H). ^13^C NMR (100 MHz, (CD_3_)_2_SO) δ 35.54, 25.30, 22.44, 14.18 (CH_3_) (CH_2_ next to B not visible; quadrupolar relaxation). ^11^B NMR (128 MHz, (CD_3_)_2_SO) δ 4.83 (d, *J* = 65.6 Hz). ^19^F NMR (377 MHz (CD_3_)_2_SO) δ –136.86 (d, *J* = 74.4 Hz). m.p. 392.2 °C.

**Potassium (1‐naphthalene) trifluoroborate** (**24**): Synthesized according to general procedure I from naphthalene‐1‐ylboronic acid (400 mg, 2.33 mmol), potassium fluoride (540 mg, 9.30 mmol) and 2,3‐dihydroxysuccinic acid (716 mg, 4.77 mmol) to afford **24** (525 mg, 97 %) as white crystals. ^1^H NMR (400 MHz, (CD_3_)_2_SO) δ 8.39 (d, *J* = 8.8 Hz, 1H), 7.74–7.69 (m, 1H), 7.57 (d, *J* = 8.2 Hz, 1H), 7.55–7.52 (m, 1H), 7.34–7.23 (m, 3H). ^13^C NMR (100 MHz, (CD_3_)_2_SO) δ 136.63, 132.99, 130.29, 128.55, 127.38, 125.20, 124.93, 123.90, 123.38 (C next to B not visible; quadrupolar relaxation). ^11^B NMR (128 MHz, (CD_3_)_2_SO) δ 3.51 (d, *J* = 56.0 Hz). ^19^F NMR (377 MHz (CD_3_)_2_SO) δ –135.27 (d, *J* = 65.0 Hz). m.p. 117.9 °C.

**Potassium (4‐methoxyphenyl) trifluoroborate** (**25**): Synthesized according to general procedure I from (4‐methoxyphenyl)boronic acid (400 mg, 2.63 mmol), potassium fluoride (612 mg, 10.5 mmol) and 2,3‐dihydroxysuccinic acid (810 mg, 5.40 mmol) to afford **25** (548 mg, 97 %) as white crystals. ^1^H NMR (400 MHz, (CD_3_)_2_SO) δ 7.21 (d, *J* = 8.4 Hz, 2H), 6.66 (d, *J* = 7.7 Hz, 2H), 3.66 (s, 3H, CH_3_). ^13^C NMR (100 MHz, (CD_3_)_2_SO) δ 157.20, 132.25, 111.87, 54.55 (C next to B not visible; quadrupolar relaxation). ^11^B NMR (128 MHz, (CD_3_)_2_SO) δ 3.44 (m). ^19^F NMR (377 MHz (CD_3_)_2_SO) δ –138.19 (m). m.p. 256.9 °C.

## Supporting information

Supporting Information
