# Supplementary material for: A Revised Modular Approach to (–)‐trans‐Δ8‐THC and Derivatives Through Late‐Stage Suzuki–Miyaura Cross‐Coupling Reactions
Source: European J Org Chem. 2019 Mar 18;2019(12):2289–96. doi: 10.1002/ejoc.201900059 (PMC6686972; doi:10.1002/ejoc.201900059)
Supplement: Supplementary file 1 — Supporting Information [file EJOC-2019-2289-s001.pdf]

EurJOC
European Journal of Organic Chemistry

Supporting Information

A Revised Modular Approach to (–)-*trans*-Δ8-THC and Derivatives Through Late-Stage Suzuki–Miyaura Cross-Coupling Reactions

Victor R. L. J. Bloemendal, Daan Sondag, Hidde Elferink,
Thomas J. Boltje, Jan. C. M. van Hest,* and Floris P. J. T. Rutjes*

ejoc201900059-sup-0001-SupMat.pdf

Table of Contents

Pages

I.	Initial Cross-Coupling Conditions on **4**	S2
II.	NMR Data Comparison Compound **5** and **6**	S3-S5
III.	NMR Data Comparison of Regioisomers **10c** and **11c**	S6
IV.	NMR Data Comparison of Atropisomers **10b-*R*$_a$** and **10b-*S*$_a$**	S7-S9
**V.**	^{1}H, ^{13}C, ^{11}B and ^{19}F NMR Spectra	S10-S46

I. Initial Cross-Coupling Conditions on 4

Table 1. Initial cross-coupling conditions on **4** towards (–)-*Trans*-Δ[8]-THC derivatives**.**

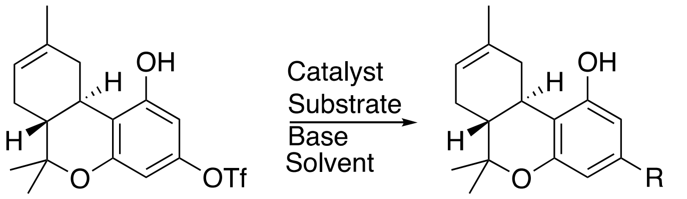

(-)-Δ8-THC-OTf, (**4**) (-)-Δ8-THC derivative

Entry[a]	Catalyst	Substrate	Base	Solvent	Conv.[b]
I.	6% Pd(dppf)Cl_2	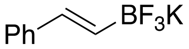	Cs_2CO_3	MeOH	0%
II.	3% Pd$(OAc)_2$	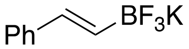	NaOH	THF	0%
III.	9% Pd(dppf)Cl_2	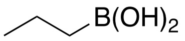	K_2CO_3	PhMe	0%
IV.	9% Pd(dppf)Cl_2	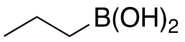	K_2CO_3	THF:H_2O (10:1)	0%
V.	9% Pd(dppf)Cl_2	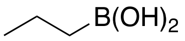	NaOH	THF:H_2O (10:1)	0%
VI.	9% Pd(dppf)Cl_2	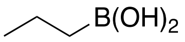	Cs_2CO_3 Ag_2O (2 eq)	THF	0%
VII.	2% Pd(dppf)Cl_2	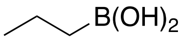	KOAc	THF	0%
VIII.	5% Pd(dppf)Cl_2	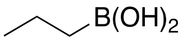	K_3PO_4	PhMe	0%
IX.	2% $Pd_2(dba)_3$ + 5% RuPhos	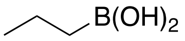	K_3PO_4	PhMe	0%
X.	5% $Pd(PPh_3)_4$	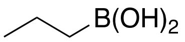	Cs_2CO_3	PhMe	0%

[a]Reaction conditions: Substrate (1.6 eq), base (3 eq) and catalyst (mol%) combined, flask evacuated and backfilled with Ar thrice. (**4**) Was added in solvent and refluxed for 16h. [b]Conversion determined using crude ^{1}H-NMR.

II. NMR Data Comparison Compounds 6 and 7

Table 2: ^{1}H-NMR and ^{13}C-NMR comparison of the two different THC bromide isomers **6** and **7**.

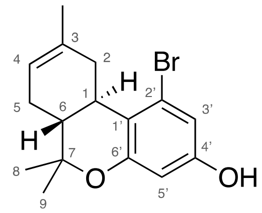

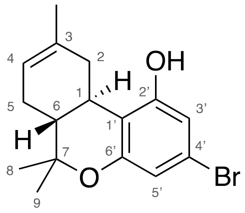

**POSITION**	**^{1}H-NMR**	**^{13}C-NMR**	**^{1}H-NMR**	**^{13}C-NMR**
1	2.64 (td, *J* = 10.5, 4.3 Hz)	35.15	2.66 (td, *J* = 11.1, 4.8 Hz)	31.70
2	2a) 3.41 (dd, *J* = 16.4, 3.4 Hz),<br>2b) 1.86 (m)	36.80	2a) 3.16 (dd, *J* = 15.7, 4.4 Hz)<br>2b) 1.84 – 1.74 (m)	35.81
3	 1.71 (s)	134.84<br>23.56 (3-Me)	 1.70 (s)	134.67<br>23.58 (3-Me)
4	5.46 – 5.41 (m)	119.64	5.46 – 5.40 (m)	119.43
5	5a) 2.18 – 2.11 (m),<br>5b) 1.86 (m)	28.41	5a) 2.19 – 2.09 (m)<br>5b) 1.84 – 1.74 (m)	27.93
6	1.86 (m)	46.55	1.84 – 1.74 (m)	44.81
7	-	77.51	-	77.62
8	1.37 (s)	27.41	1.38 (s)	27.53
9	1.07 (s)	18.29	1.09 (s)	18.58
1′	-	118.78	-	112.74
2′	-	123.71	5.24 (s)	155.94
3′	**6.68 (d, *J* = 2.6 Hz)**	113.64	**6.61 (d, *J* = 1.9 Hz)**	113.82
4′	5.05 (s)	155.01	-	119.77
5′	**6.28 (d, *J* = 2.6 Hz)**	104.40	**6.43 (d, *J* = 1.9 Hz)**	110.84
6′	-	155.81	-	155.79

The difference between **6** and **7** can be seen in the essential difference in shifts of the two aromatic protons (3′ and 5′) and their corresponding 0.7 Hz difference in *J*-coupling (Figure 1). The difference between the two isomers can also be visualized using TLC (0.12 difference in Rf (EtOAc/*n*-heptane, 1/9 v/v), Figure 2). These compounds can easily be separated *via* silicagel column chromatography using an apolar eluent and slow gradient (0 - 4% - EtOAc in *n*-heptane). Best results were obtained using dry loading the crude mixture, a high amount of silicagel and starting with pure heptane (10-20 CV) and slowly going up to 4% EtOAc (10-20 CV).

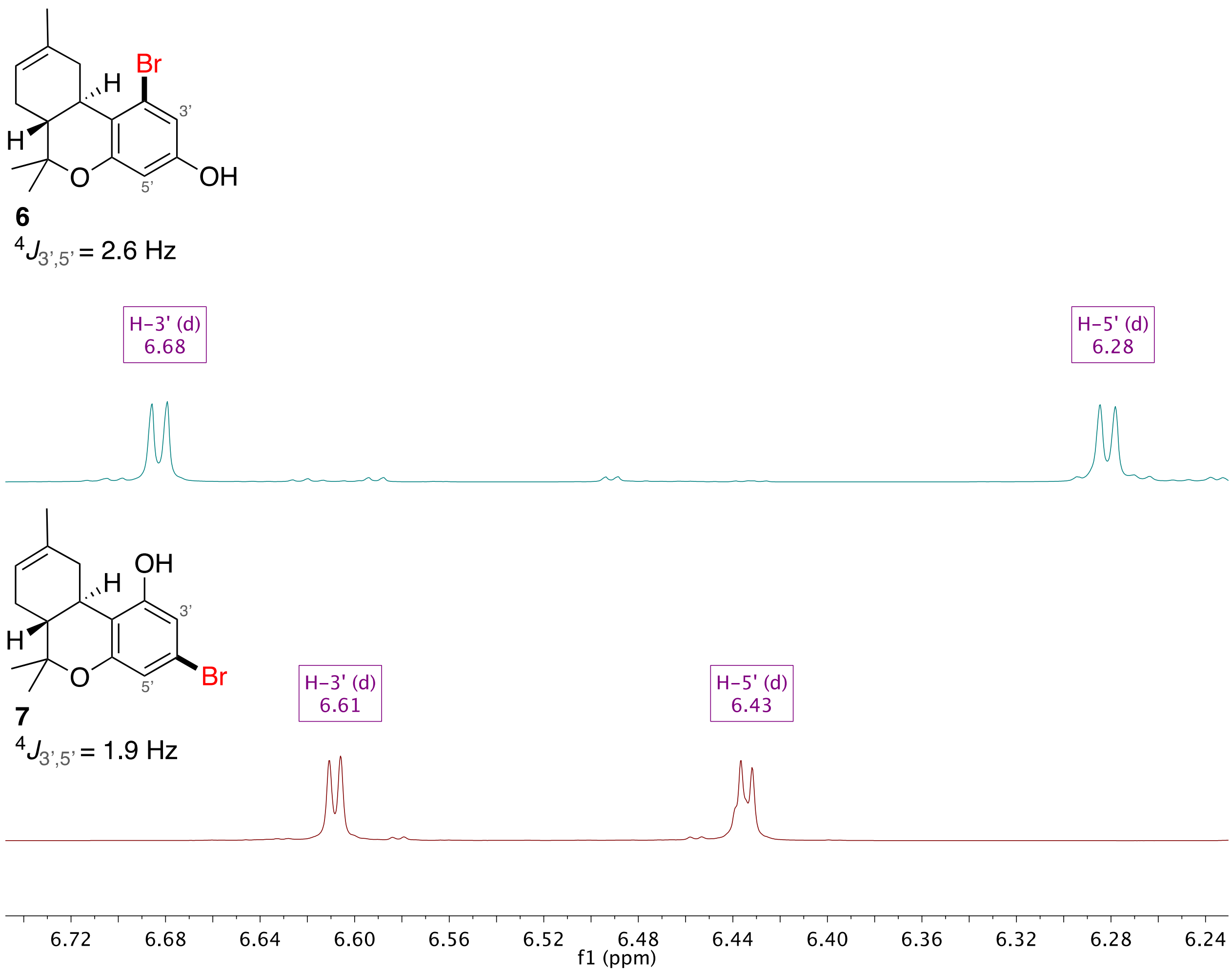

Figure 1: ^{1}H-NMR spectrum of aryl protons H-3' and H-5' of both isomers **6** and **7** and their difference in shift and $^4J_{3',5'}$-coupling constant.

Assignment was performed with ^{1}H-^{13}C-HMBC spectroscopy. *Ortho* substituted product **6** shows a coupling of H-1 with carbon signal at 123.71 ppm, belonging to C-2' of the aryl-bromide (Figure 3). This three bond coupling is visible in the *ortho* substituted product **6** but not in *para* substituted **7** (119.77 ppm, C-4'). The lack of this signal indicates that the bromide is substituted at positions as assigned in Table 1. Extra evidence was found when **7** was converted into the readily known and synthesized (–)-*Trans*-Δ^8-tetrahydrocannabinol (**1**).

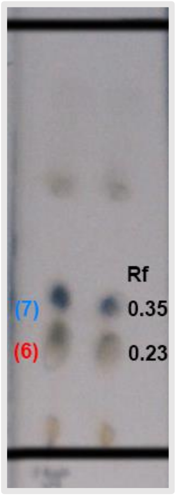

Figure 2: Corresponding difference on a TLC plate (EtOAc/*n*-heptane, 1/9 v/v) of the crude mixture of **6** and **7**.

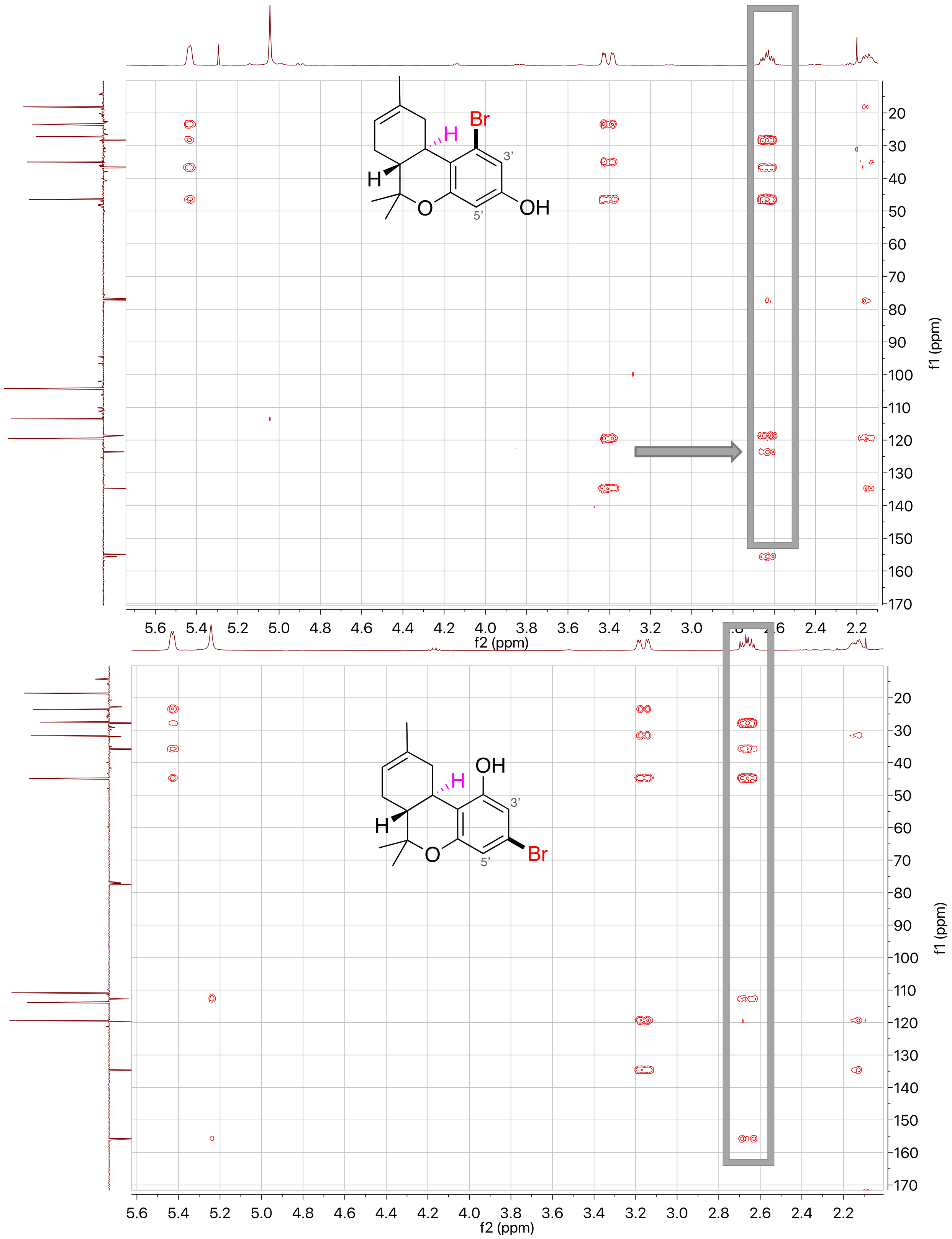

Figure 3: HMBC spectroscopy of **6** and **7** where **6** shows 3-bond coupling with C-Br (C-2') signal and **7** does not (C-4', 5-bond coupling).

III. NMR Data Comparison of Regioisomers 10c and 11c

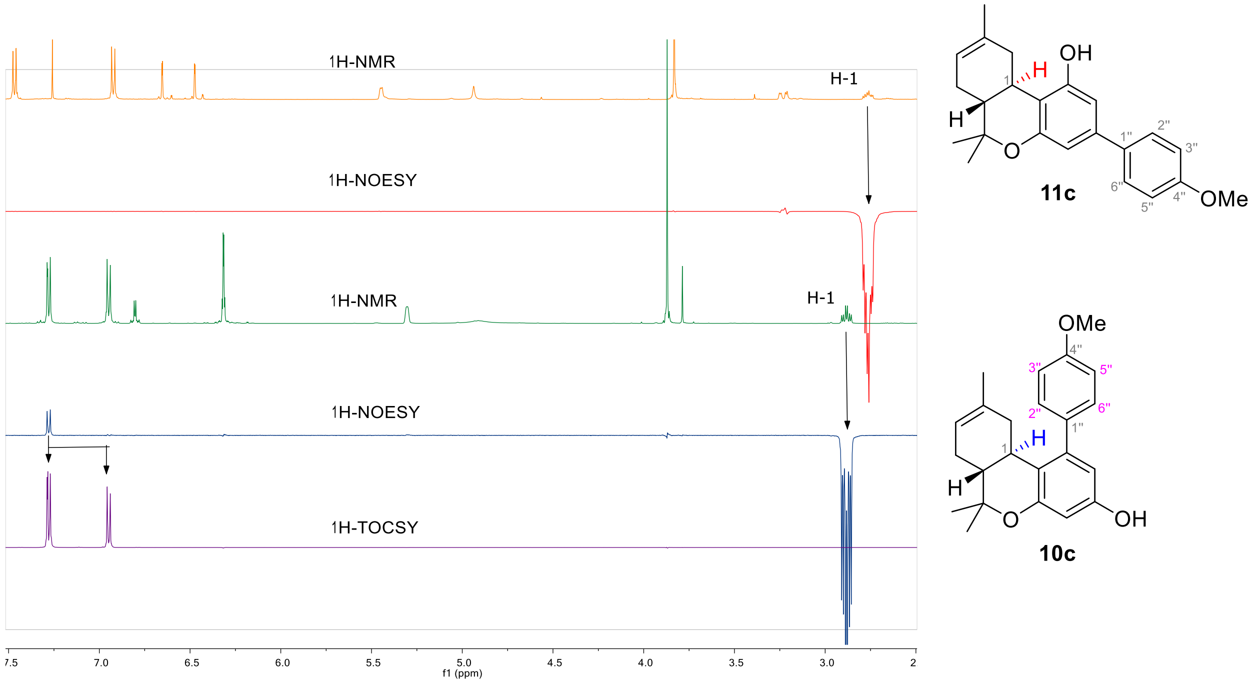

Figure 4: selective 1D ^{1}H-NOESY and ^{1}H-TOCSY NMR of regioisomers **11c** and **10c**, proving the spectroscopic difference Top to bottom: 1 (**Orange**) ^{1}H-NMR of **11c**. 2 (**Red**) ^{1}H-NOESY of 2.76 ppm of **11c**, showing no neighboring protons and is therefore *para* substituted product **11c**. 3 (**Green**) ^{1}H-NMR of **10c**. 4 (**Blue**) ^{1}H-NOESY of 2.88 ppm showing a correlation to 7.28 ppm (H-2″, H-6″). 4 (**Purple**) ^{1}H-TOCSY of 7.28 ppm showing a correlation with 6.95 ppm (H-3″, H-5″) and is therefore ortho substituted product **10c**.

IV. NMR Data Comparison of Atropisomers 10b-R_a and 10b-S_a

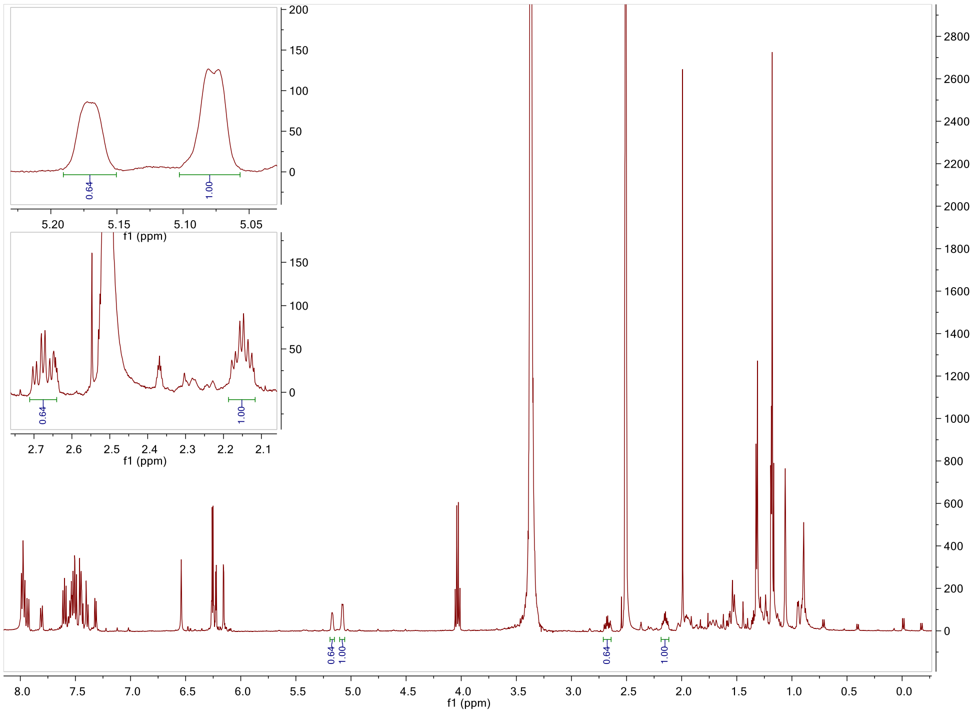

Figure 5: ^{1}H-NMR spectrum of **10b** in D_6-DMSO and its observed isomers.

Following from the NMR data as shown in Figure 5, two isomers were identified and hypothesized rotamers. The rotamer was not confirmed using ^{1}H-EXSY and VT-NMR (T = 373 K) (data not shown). This result was proven using 1D-selective ^{1}H-TOCSY-NMR (Figure 6), showing only one isomer and no correlation between isomers.

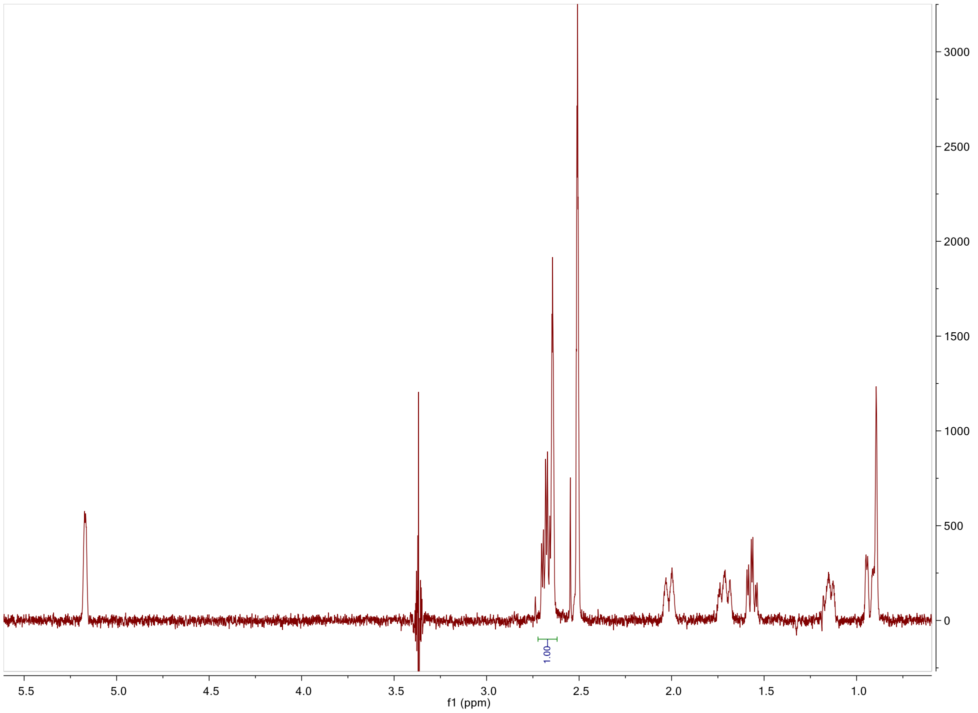

Figure 6: Selective-1D ^{1}H-TOCSY NMR on 2.67 ppm showing one isomer of **10b.**

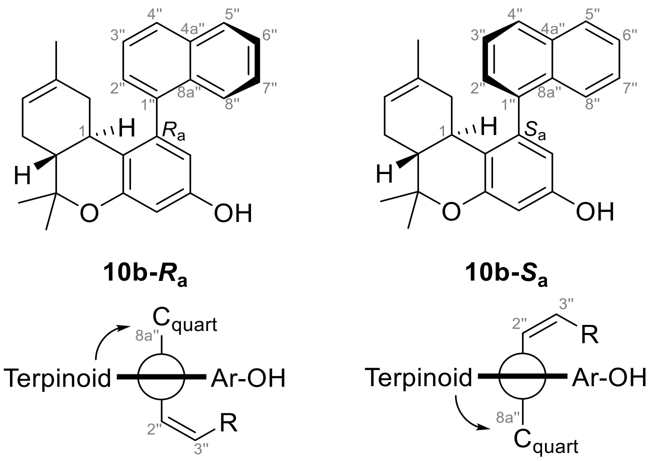

Figure 7: Structural difference between atropisomers **10b-R_a** and **10b-S_a** with the corresponding Newman projections.

10b Exists of two atropisomers, **10b-R_a** and **10b-S_a** (Figure 7). The orientation of the naphthalene moiety in isomers, causes a difference in chemical shift is observed in the ^{1}H-NMR due to the orientation of π-orbitals. To distinguish between both atropisomers, selective 1D ^{1}H-NOESY and ^{1}H-TOCSY NMR elucidated the individual molecules. In each isomer the central proton (H-1) shows a ^{1}H-NOESY correlation with one aromatic signal. A following ^{1}H-TOCSY experiment on the aromatic signal directly quantifies the naphthalene protons, and elucidates which signals belong to which atropisomer of **10b**. In this case **10b-S_a** is observed in higher amounts than **10b-R_a** (1.00 : 0.64 ratio).

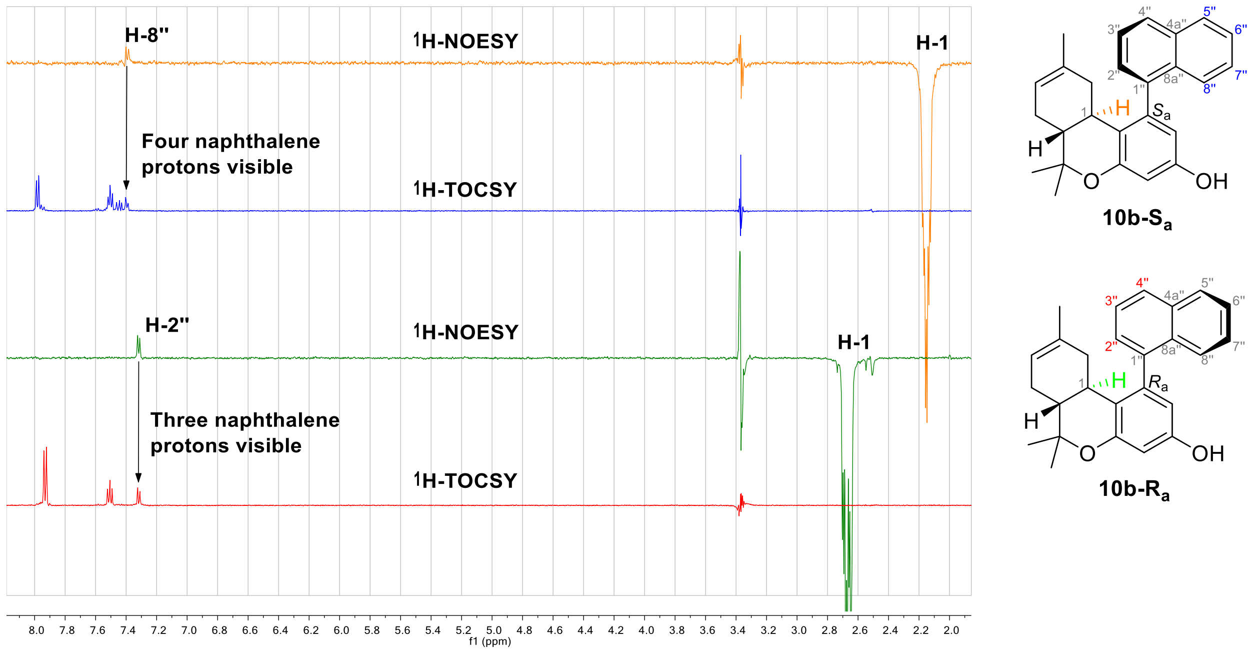

Figure 8: selective 1D ^{1}H-NOESY and ^{1}H-TOCSY NMR of both atropisomers of **10b**. Top to bottom: 1 (**orange**) ^{1}H-NOESY of 2.15 ppm showing a correlation to 7.39 ppm (H-8"). 2 (**blue**) ^{1}H-TOCSY of 7.39 ppm showing four naphthalene protons (H-5" to H-8") of compound **10b-S_a**. 3 (**green**) ^{1}H-NOESY of 2.67 ppm showing a correlation to 7.32 ppm (H-2"). 4 (**red**) ^{1}H-TOCSY of 7.32 ppm showing three naphthalene protons (H-2" to H-4") of compound **10b-R_a**

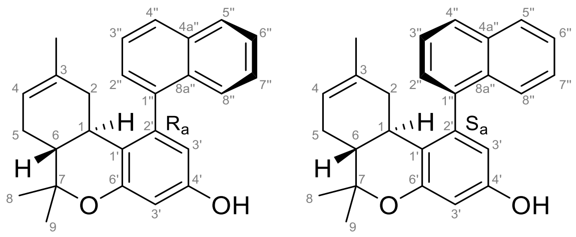

2′-Naphthalene(–)-*trans*-Δ^8-tetrahydrocannabinol (10b): Synthesized according to general procedure (II) from 2′-bromo-(–)-*trans*-Δ^8-tetrahydrocannabinol (**6**) (150.0 mg, 464 µmol) and potassium (1-naphthalene) trifluoroborate (**24**) (174.0 mg, 743 µmol) and purified using preparative HPLC which afforded (**10b**) (28.5 mg, 17%) as a colorless oil. The product was obtained as an inseparable mixture of two atropisomers **10b-R$_a$** and **10b-S$_a$** in respective ratio of 0.64:1.00. **TLC** (EtOAc/*n*-heptane, 1/9 v/v): R_f = 0.15

10b-*R*$_a$: ^{1}H NMR (500 MHz, DMSO-d$_6$) δ 7.94 (d, J = 8.2 Hz, 1H, CH Ar), 7.81 (d, J = 7.9 Hz, 1H, CH Ar), 7.56 (dd, J = 6.8, 1.6 Hz, 1H, CH Ar), 7.56 – 7.52 (m, 1H, CH Ar), 7.45 – 7.43 (m, 1H, CH Ar), 7.41 – 7.39 (m, 1H, CH Ar), 7.32 (dd, J = 7.1, 1.2 Hz, 1H, CH Ar), 6.23 (d, J = 2.6 Hz, 1H, CH Ar, H-5′), 6.16 (d, J = 2.6 Hz, 1H, CH Ar, H-3′), 5.18 (d, J = 2.7 Hz, 1H, CH Alkene, H-4), 2.69 (dt, J = 11.2, 5.6 Hz, 1H, H-1), 2.04 (m, 1H, H-5a), 1.77 – 1.67 (m, 1H, H-5b), 1.58 (dd, J = 11.6, 4.4 Hz, 1H, H-6), 1.33 (s, 3H, CH_3, H-8), 1.19 (s, 3H,CH_3, H-9), 1.17 (s, 1H, H-2a), 0.93 (d, J = 12.6 Hz, 1H, H-2b), 0.92 – 0.86 (bs, 3H, H-10). **^{13}C NMR** (126 MHz, DMSO-d$_6$) δ 156.09 (C_{quart} C-6′), 155.36 (C_{quart} Naphthalene C-4′), 140.94 (C_{quart} C-1″), 140.22 (C_{quart} C-2′), 133.93 (C_{quart} Naphthalene C-8a″), 133.30 (C Alkene C-3), 132.06 (C_{quart} Naphthalene C-4a″), 128.49 (C-Ar), 127.63 (C-Ar), 126.62 (C-Ar), 126.36 (C-Ar), 126.29 (C-Ar), 125.92 (C-Ar), 125.85 (C-Ar), 119.94 (C Alkene C-4), 115.78 (C_{quart} C-1′), 111.97 (C-5′), 103.51 (C-3′), 76.41 (C_{quart} C-7), 45.15 (C-6), 36.65 (C-2), 32.61 (C-1), 27.75 (C-8), 27.56 (C-5), 23.14 (C-10), 18.86 (C-9).

10b-*S*$_a$: **^{1}H NMR** (500 MHz, DMSO-d$_6$) δ 8.00 – 7.98 (m, 2H, CH Ar), 7.60 (dd, *J* = 8.3, 7.0 Hz, 1H, CH Ar), 7.51 (d, *J* = 1.5 Hz, 1H, CH Ar), 7.45 (m, 2H, CH Ar), 7.41 – 7.39 (m, 1H, CH Ar), 6.27 (d, *J* = 2.6 Hz, 1H, CH Ar, H-3′), 6.25 (d, *J* = 2.6 Hz, 1H, CH Ar, H-5′), 5.08 (d, *J* = 4.3 Hz, 1H, CH Alkene, H-4), 2.16 (td, J = 10.7, 4.9 Hz, 1H, H-1), 1.97 – 1.93 (m, 1H, H-5a), 1.55 – 1.53 (m, 2H, H-6, H-5b), 1.32 (s, 3H, CH_3, H-8), 1.31 – 1.29 (m, 1H, H-2a), 1.18 (s, 3H, CH_3, H-9), 1.06 (s, 3H, CH_3, H-10), 0.97 – 0.90 (m, 1H, H-2b). **^{13}C NMR** (126 MHz, DMSO-d$_6$) δ 156.85 (C_{quart} C-4′), 154.69 (C_{quart} C-6′), 141.46(C_{quart} Naphthalene C-1″), 141.14(C_{quart} C-2′), 133.30 (C_{quart} Naphthalene C-8a″), 133.26 (C_{quart} C-3), 130.55 (C_{quart} Naphthalene C-4a″), 128.83 (C-Ar), 127.89 (C-Ar), 126.93 (C-Ar), 126.67 (C-Ar), 126.45 (C-Ar), 126.09 (C-Ar), 125.41 (C-Ar), 119.68 (C Alkene C-4), 115.84 (C_{quart} C-1′), 111.66 (C-5′), 103.65 (C-3′), 76.35 (C_{quart} C-7), 45.01 (C-6), 37.58 (C-2), 33.44 (C-1), 27.75 (C-8), 27.56 (C-5), 23.31 (C-10), 18.90 (C-9). **HRMS** (m/z): $[M+H]^+$ calcd. for $C_{26}H_{26}O_2$, 371.20110; found, 371.20214.

V. ^{1}H, ^{13}C and ^{19}F NMR Spectra

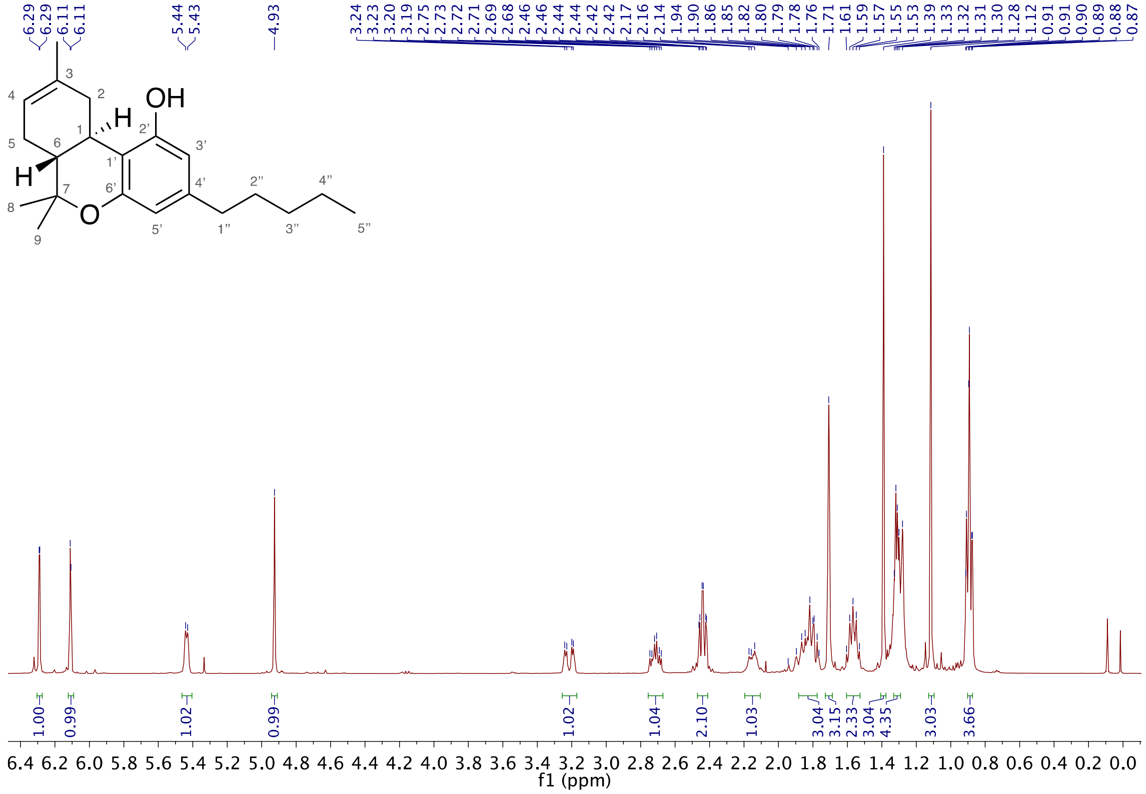

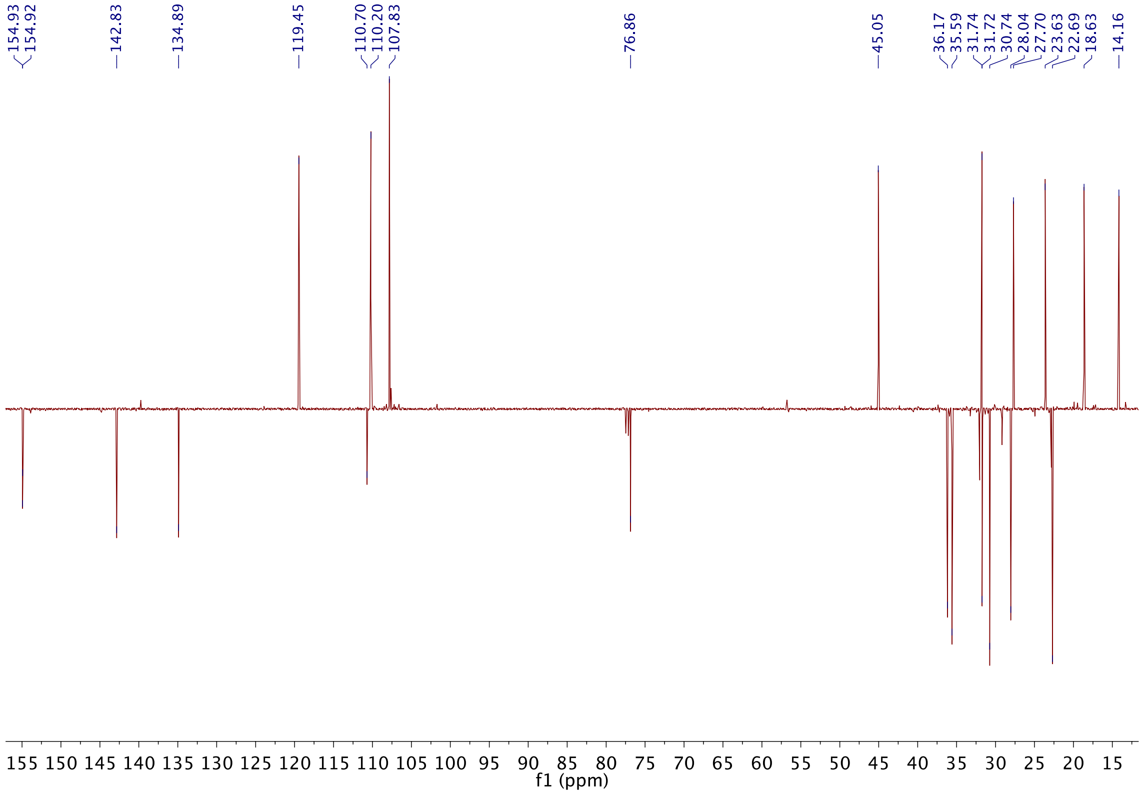

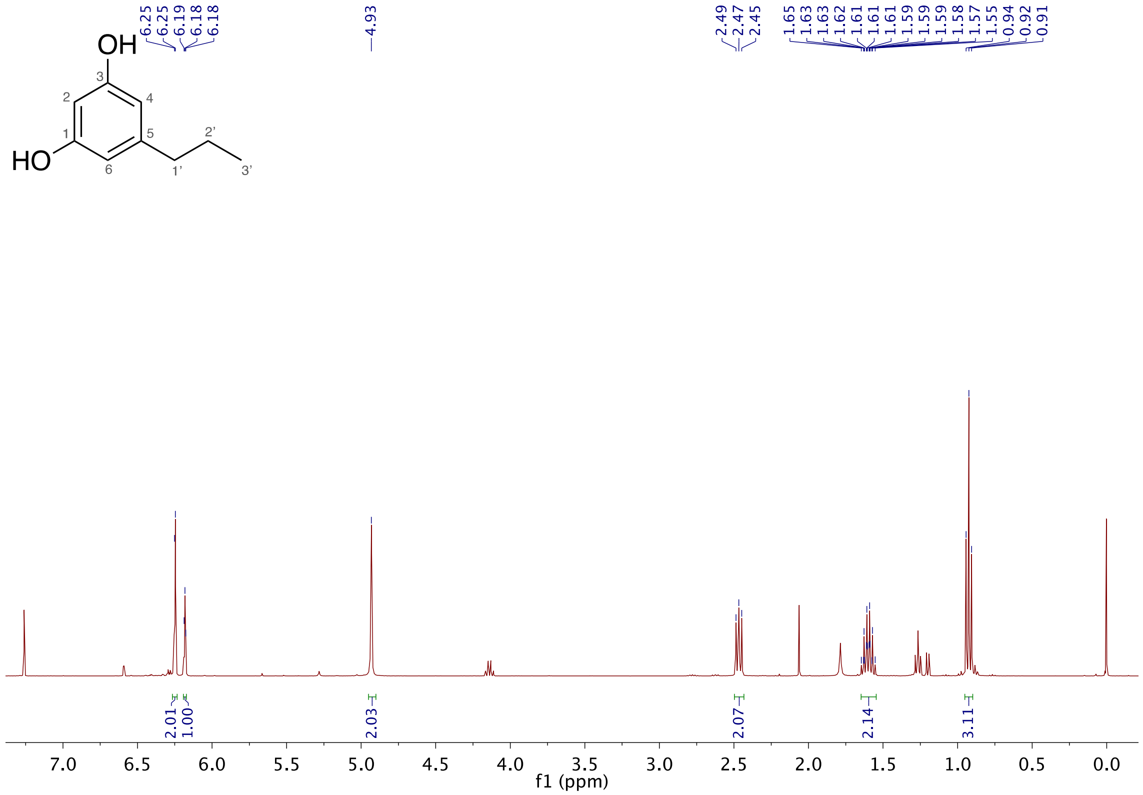
OH
HO
1
2
3
4
5
6
1'
2'
3'
6.25
6.25
6.19
6.18
6.18
4.93
2.49
2.47
2.45
1.65
1.63
1.63
1.62
1.61
1.61
1.61
1.59
1.59
1.59
1.58
1.57
1.55
0.94
0.92
0.91
2.01
1.00
2.03
2.07
2.14
3.11
7.0 6.5 6.0 5.5 5.0 4.5 4.0 3.5 3.0 2.5 2.0 1.5 1.0 0.5 0.0
f1 (ppm)

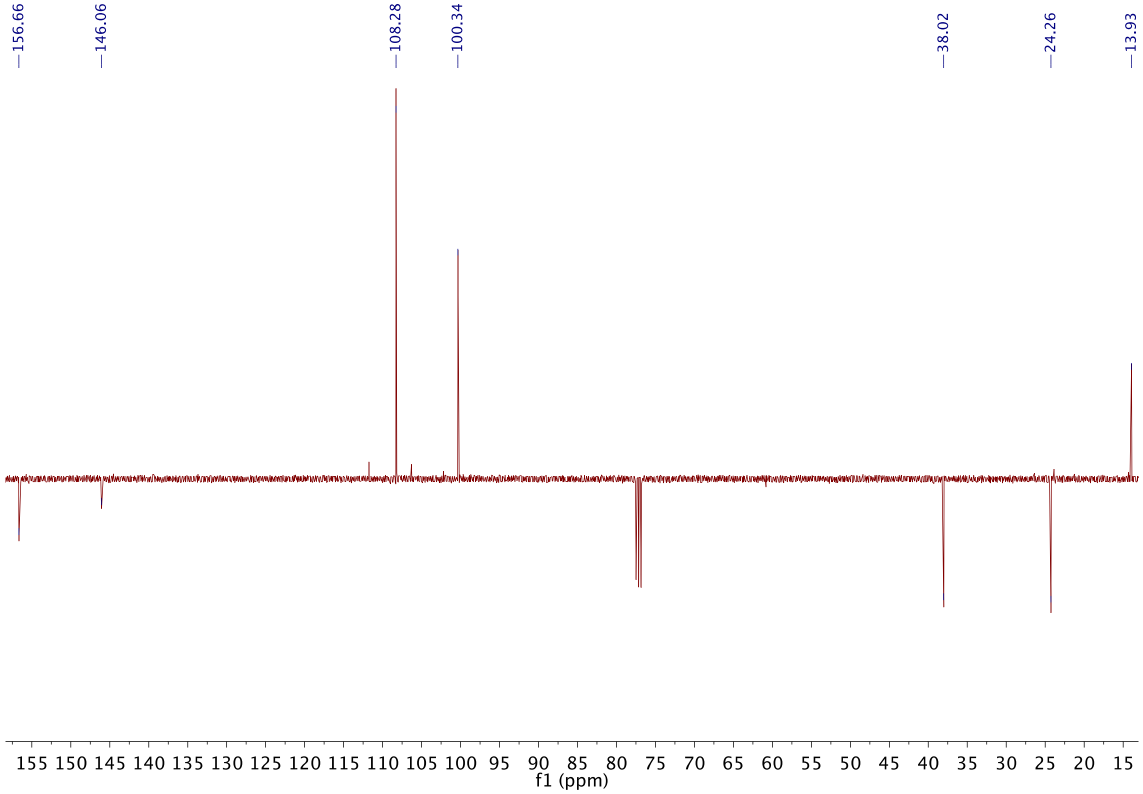
156.66
146.06
108.28
100.34
38.02
24.26
13.93
155 150 145 140 135 130 125 120 115 110 105 100 95 90 85 80 75 70 65 60 55 50 45 40 35 30 25 20 15
f1 (ppm)

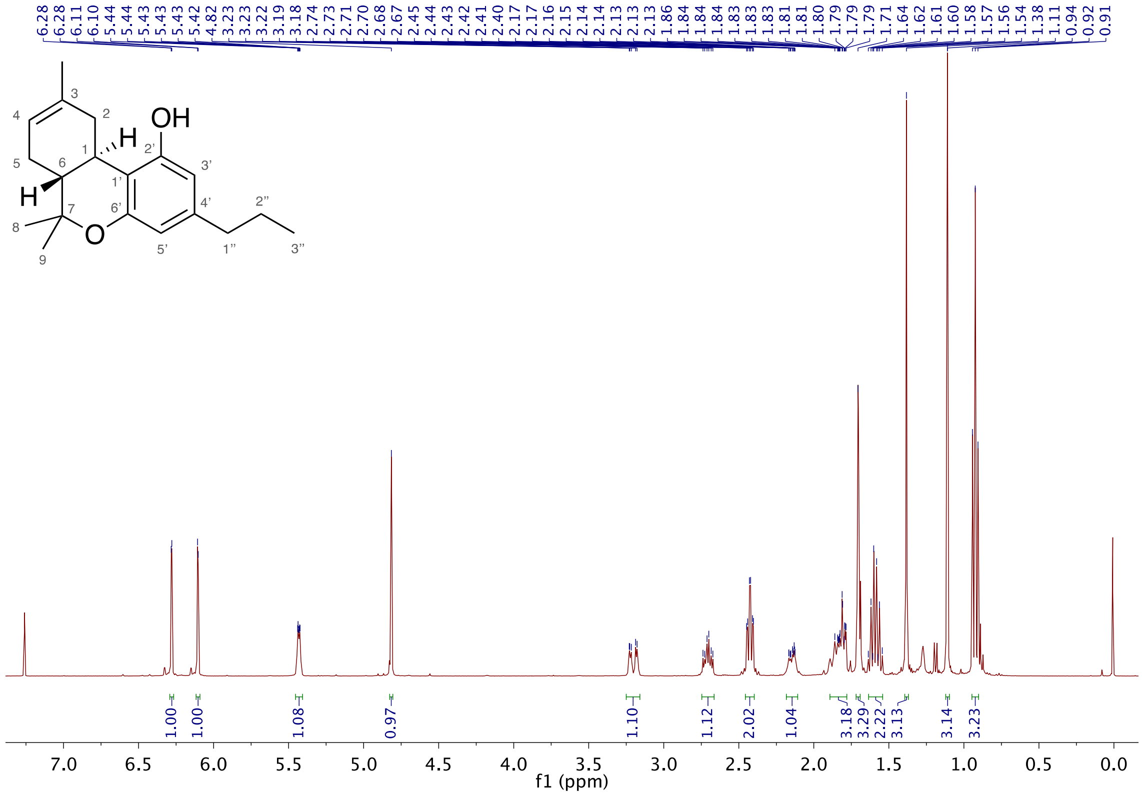

6.28 6.28 6.11 6.10 5.44 5.44 5.43 5.43 5.43 5.42 4.82 3.23 3.23 3.22 3.19 3.18 2.74 2.73 2.71 2.70 2.68 2.67 2.45 2.44 2.43 2.42 2.41 2.40 2.17 2.17 2.16 2.15 2.14 2.14 2.13 2.13 2.13 1.86 1.84 1.84 1.84 1.83 1.83 1.83 1.81 1.81 1.80 1.79 1.79 1.79 1.71 1.64 1.62 1.61 1.60 1.58 1.57 1.56 1.54 1.38 1.11 0.94 0.92 0.91
OH
H
H
O
1 2 3 4 5 6 7 8 9 1' 2' 3' 4' 5' 6' 1'' 2'' 3''
1.00 1.00 1.08 0.97 1.10 1.12 2.02 1.04 3.18 3.29 2.22 3.13 3.14 3.23
7.0 6.5 6.0 5.5 5.0 4.5 4.0 3.5 3.0 2.5 2.0 1.5 1.0 0.5 0.0
f1 (ppm)

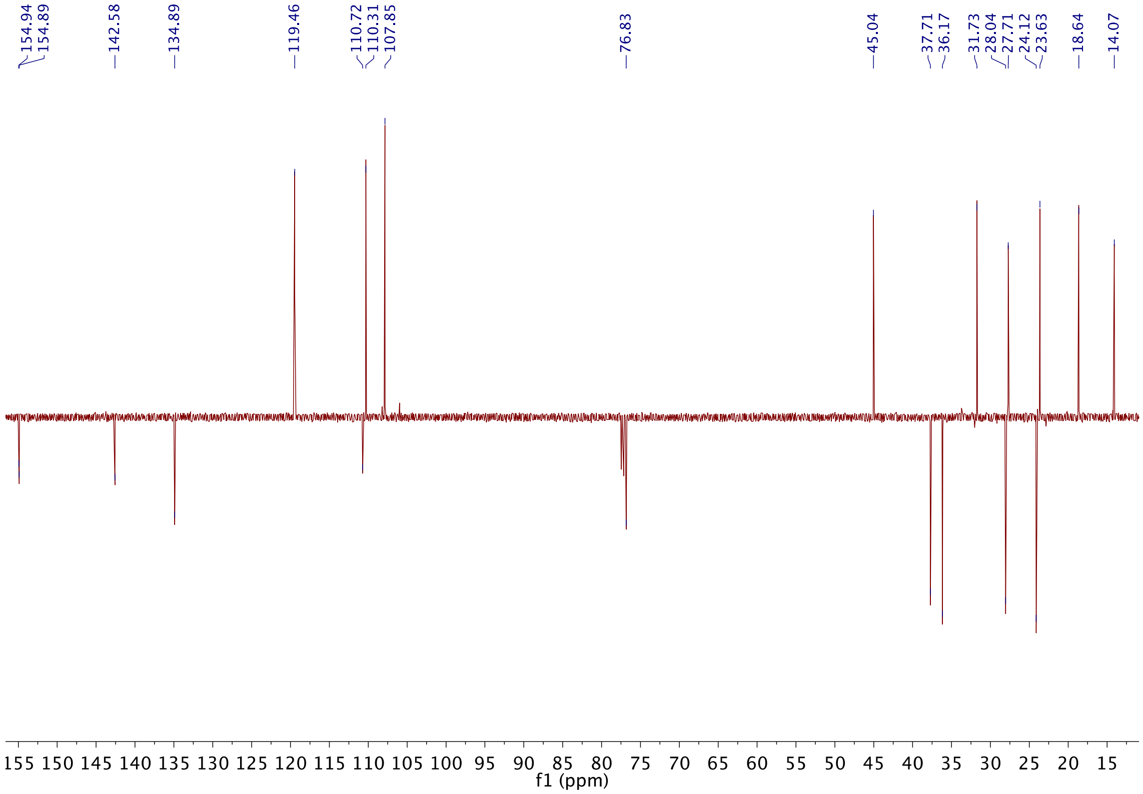

154.94 154.89 142.58 134.89 119.46 110.72 110.31 107.85 76.83 45.04 37.71 36.17 31.73 28.04 27.71 24.12 23.63 18.64 14.07
155 150 145 140 135 130 125 120 115 110 105 100 95 90 85 80 75 70 65 60 55 50 45 40 35 30 25 20 15
f1 (ppm)

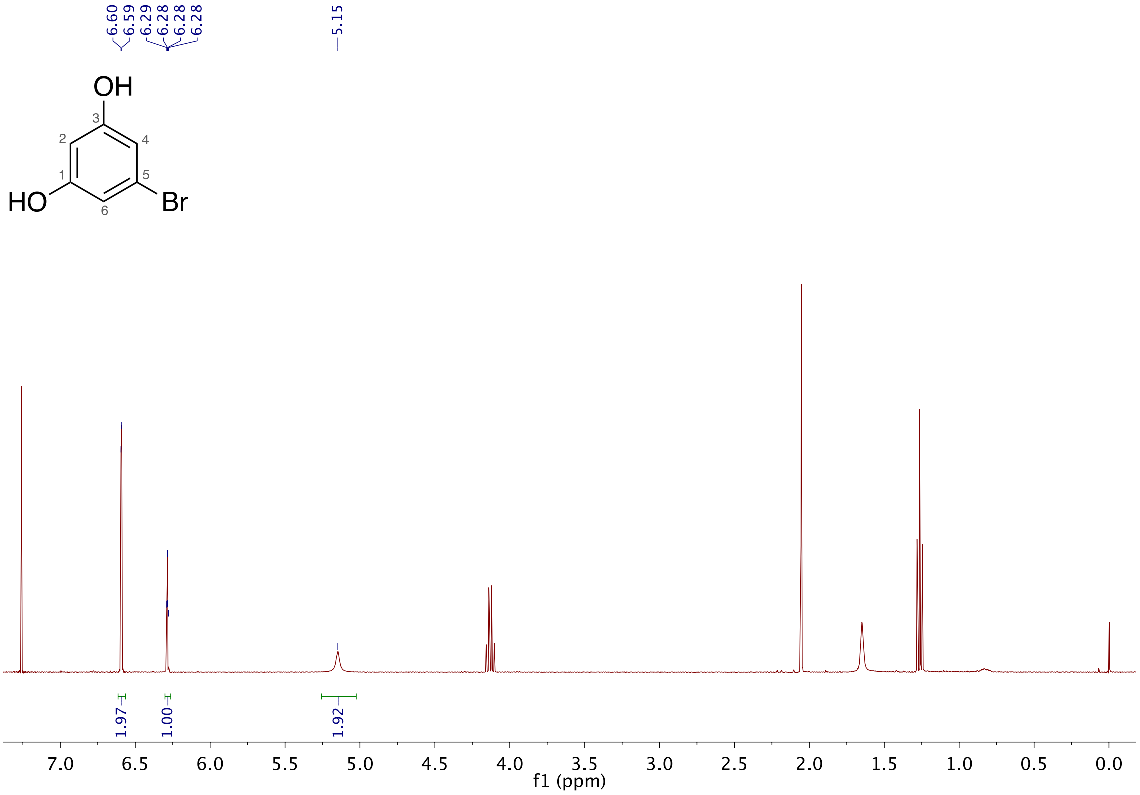
6.60
6.59
6.29
6.28
6.28
6.28
5.15
OH
HO
Br
1.97
1.00
1.92
7.0 6.5 6.0 5.5 5.0 4.5 4.0 3.5 3.0 2.5 2.0 1.5 1.0 0.5 0.0
f1 (ppm)

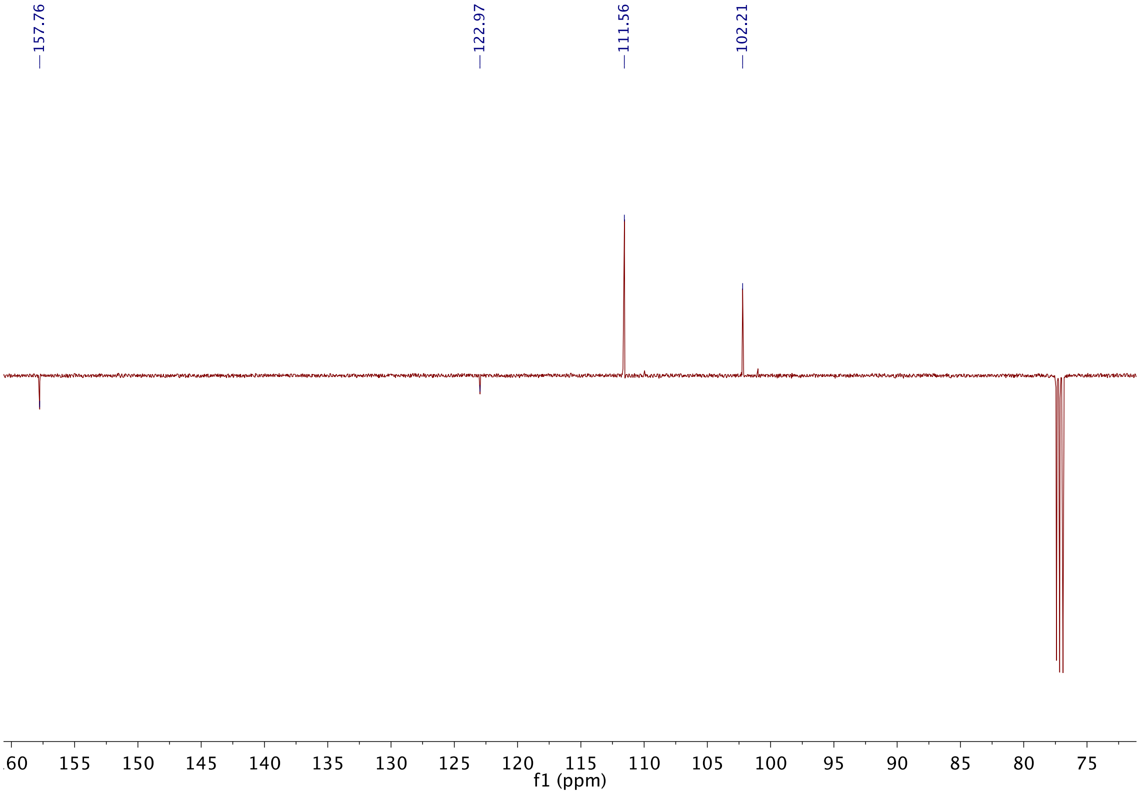
157.76
122.97
111.56
102.21
160 155 150 145 140 135 130 125 120 115 110 105 100 95 90 85 80 75
f1 (ppm)

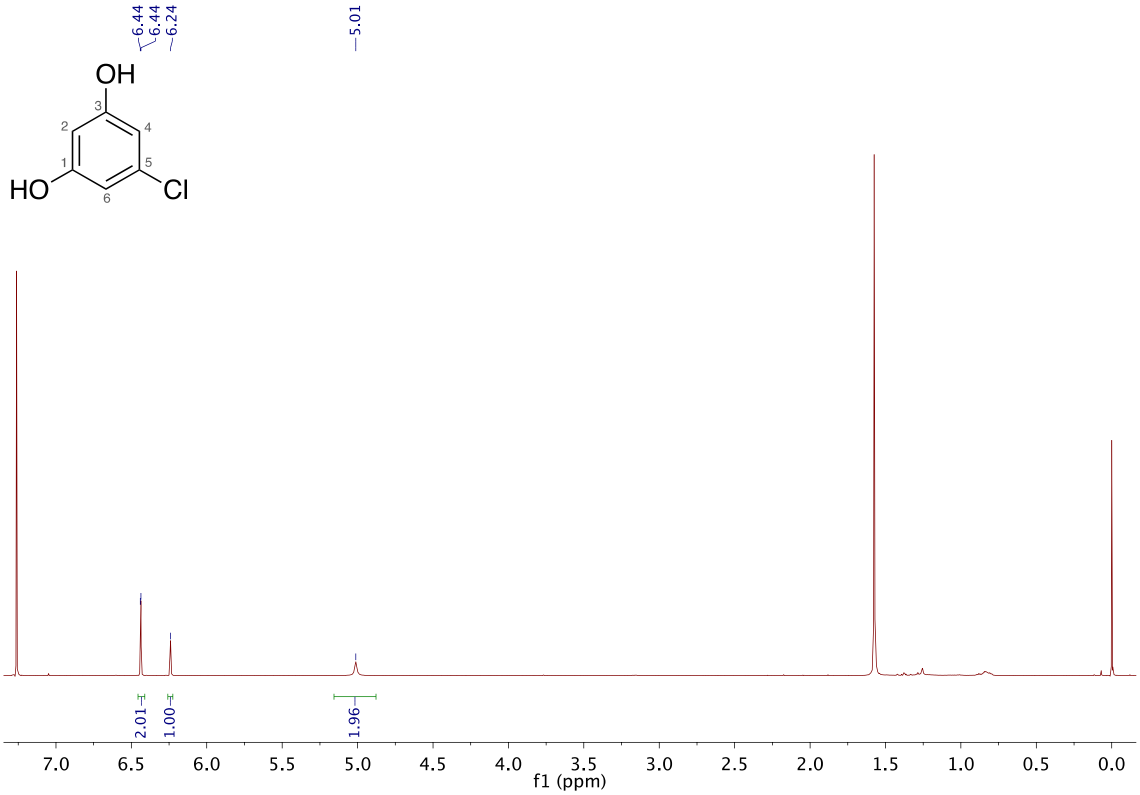
6.44
6.44
6.24
5.01
OH
HO
Cl
1
2
3
4
5
6
2.01
1.00
1.96
7.0 6.5 6.0 5.5 5.0 4.5 4.0 3.5 3.0 2.5 2.0 1.5 1.0 0.5 0.0
f1 (ppm)

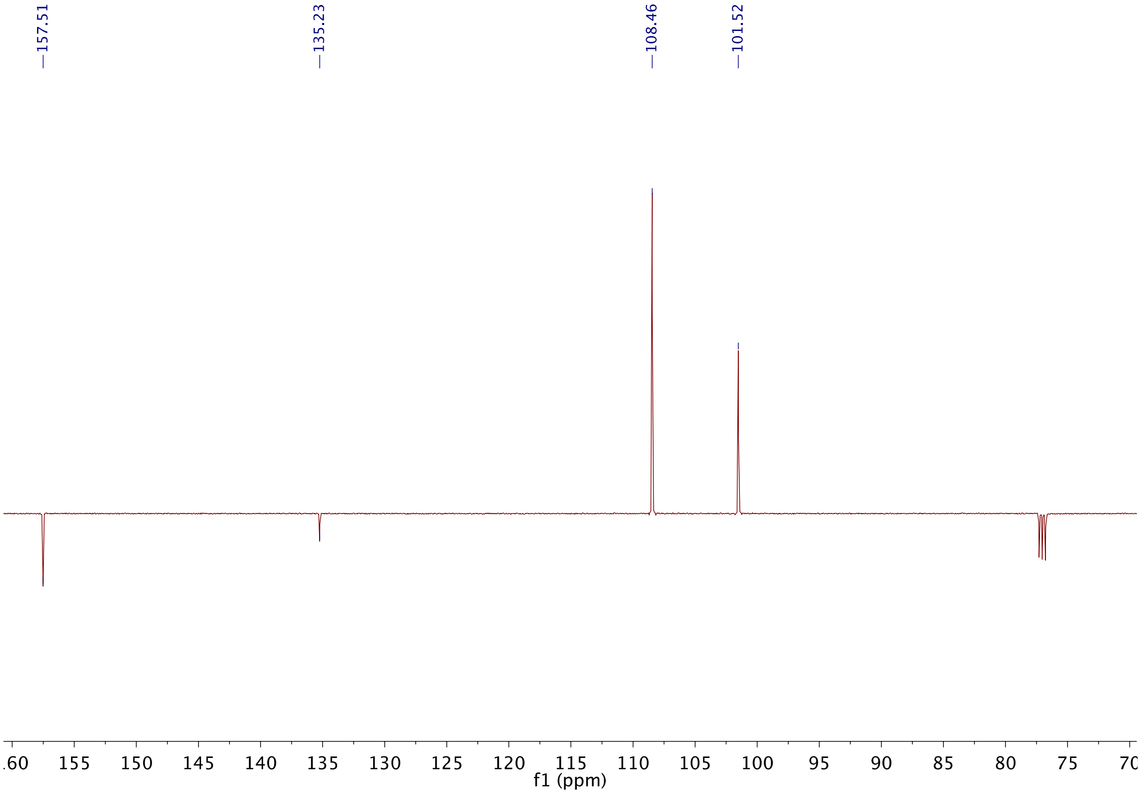
157.51
135.23
108.46
101.52
.60 155 150 145 140 135 130 125 120 115 110 105 100 95 90 85 80 75 70
f1 (ppm)

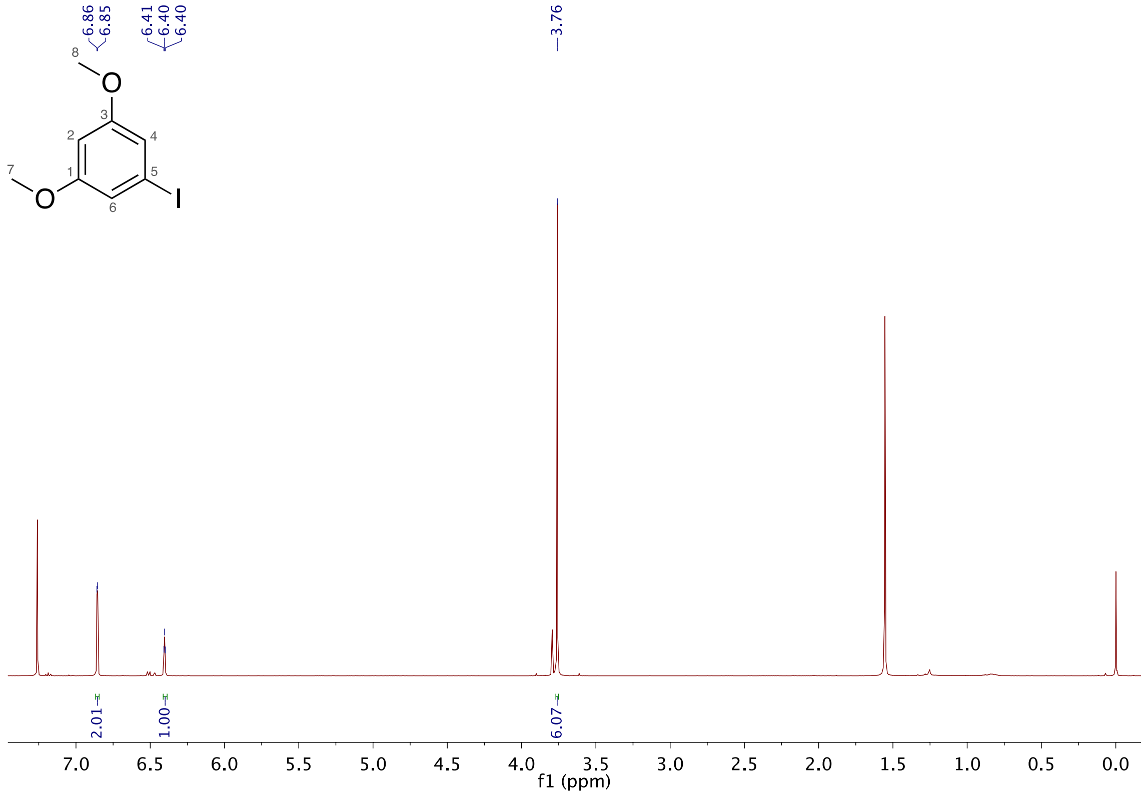

8
3
2
4
7
1
5
6
O
O
I
6.86
6.85
6.41
6.40
6.40
3.76
2.01
1.00
6.07
7.0 6.5 6.0 5.5 5.0 4.5 4.0 3.5 3.0 2.5 2.0 1.5 1.0 0.5 0.0
f1 (ppm)

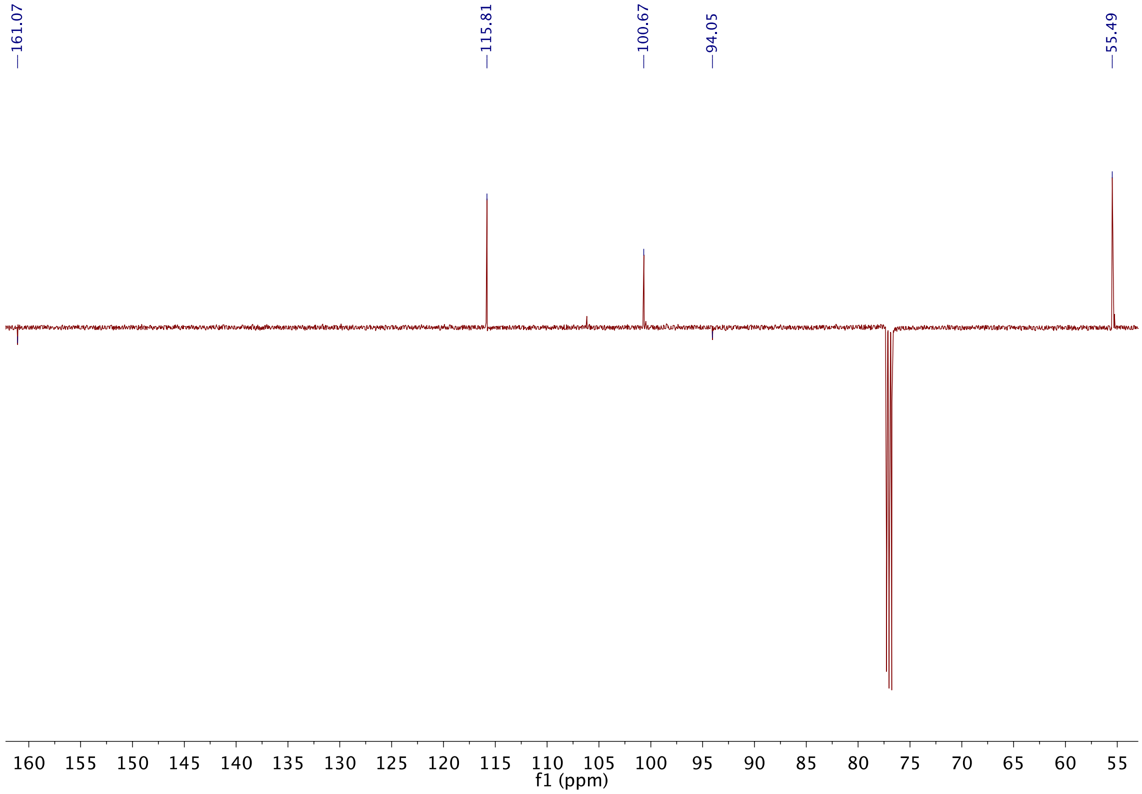

161.07
115.81
100.67
94.05
55.49
160 155 150 145 140 135 130 125 120 115 110 105 100 95 90 85 80 75 70 65 60 55
f1 (ppm)

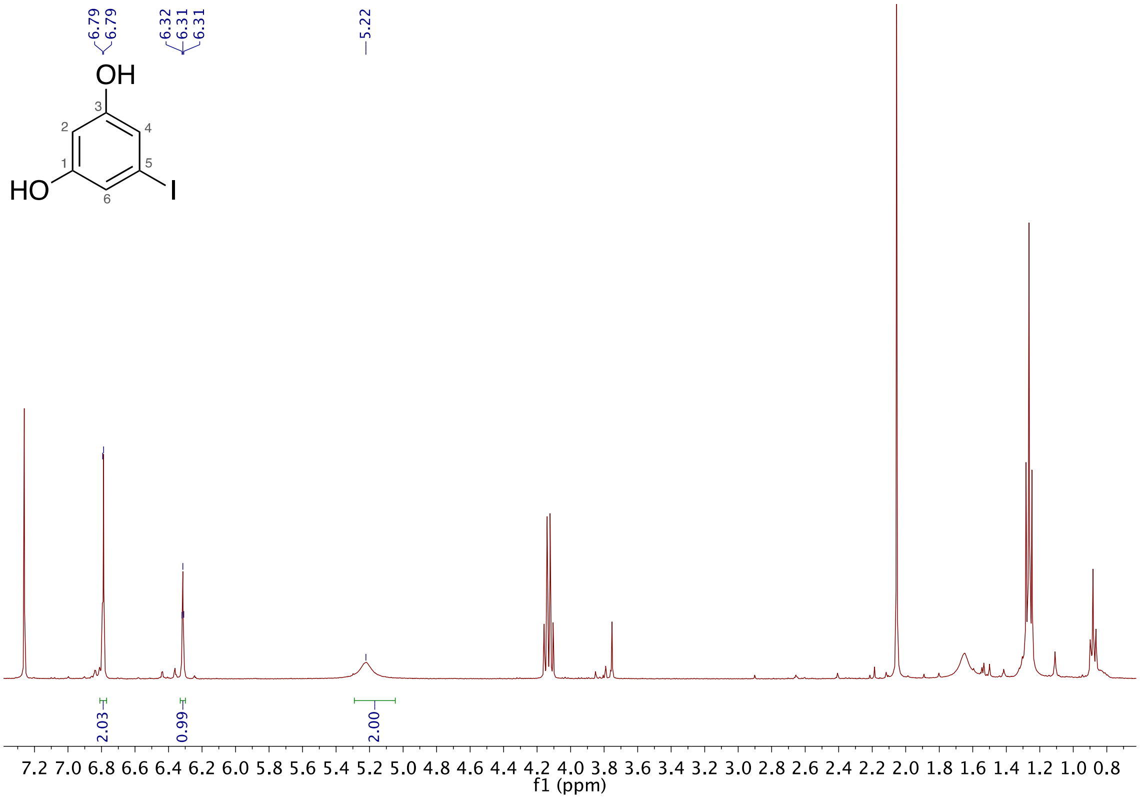

6.79
6.79
6.32
6.31
6.31
5.22
OH
HO
I
1
2
3
4
5
6
2.03
0.99
2.00
7.2 7.0 6.8 6.6 6.4 6.2 6.0 5.8 5.6 5.4 5.2 5.0 4.8 4.6 4.4 4.2 4.0 3.8 3.6 3.4 3.2 3.0 2.8 2.6 2.4 2.2 2.0 1.8 1.6 1.4 1.2 1.0 0.8
f1 (ppm)

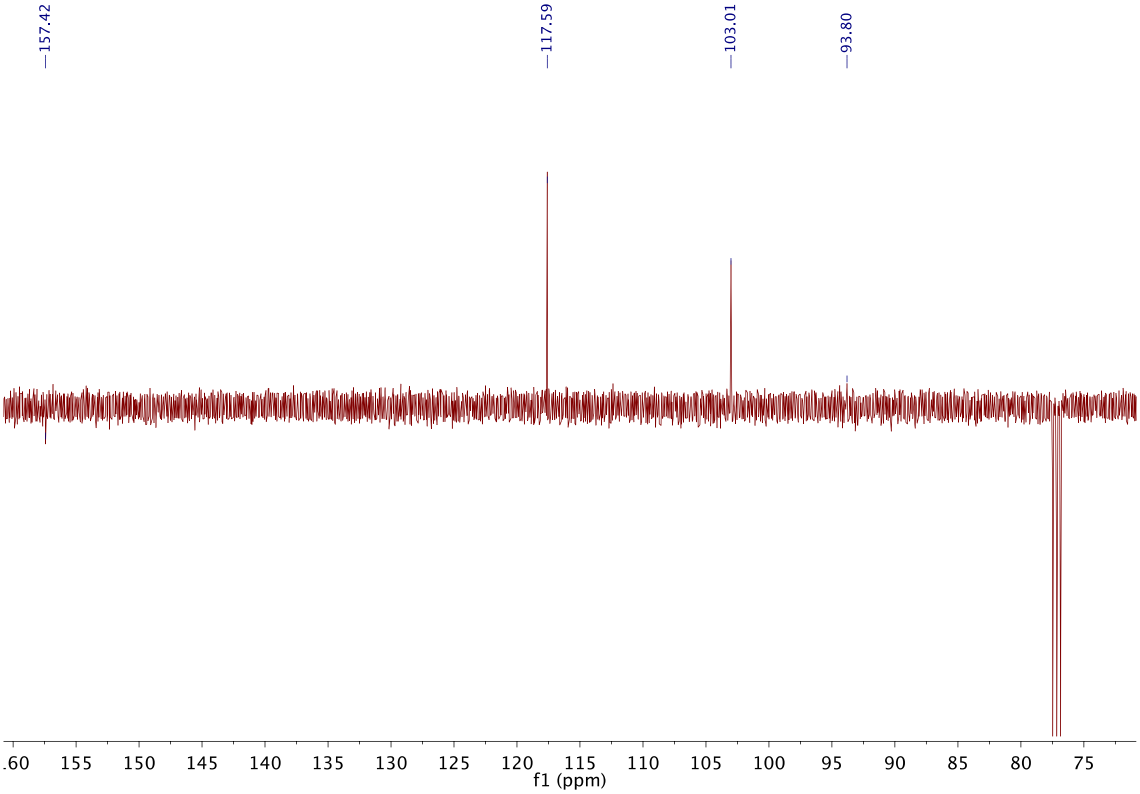

157.42
117.59
103.01
93.80
.60 155 150 145 140 135 130 125 120 115 110 105 100 95 90 85 80 75
f1 (ppm)

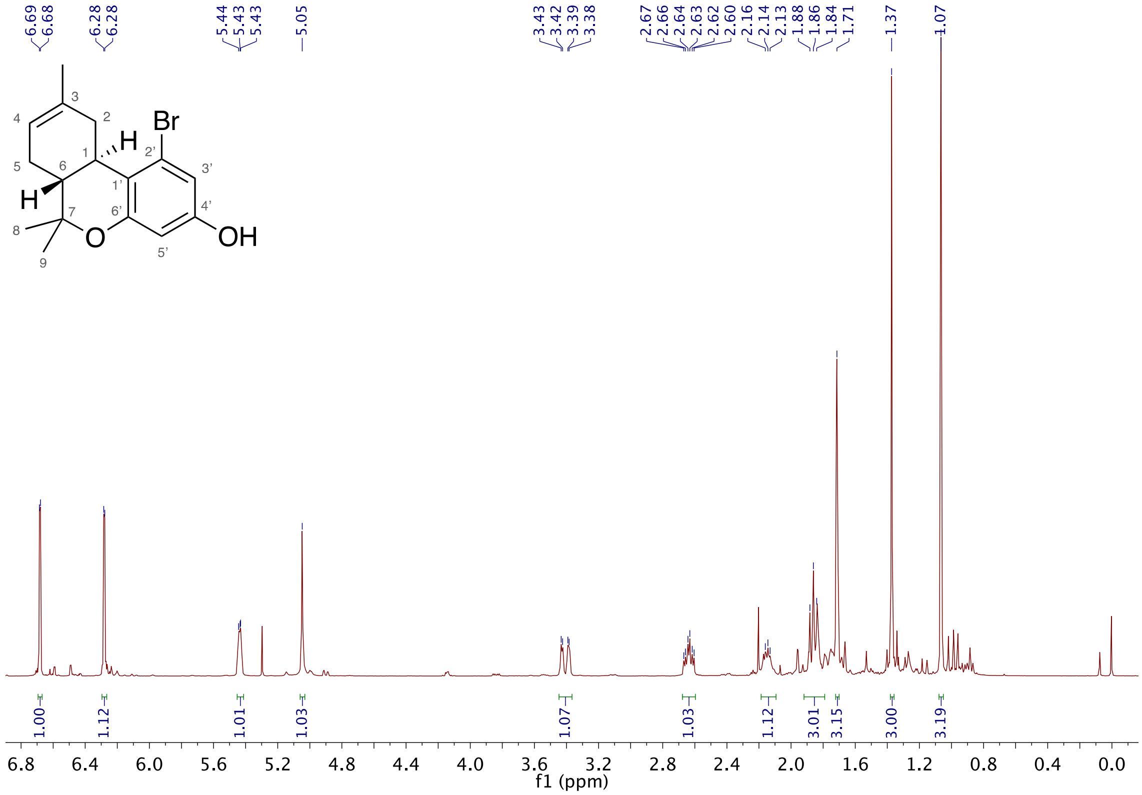
6.69
6.68
6.28
6.28
5.44
5.43
5.43
5.05
3.43
3.42
3.39
3.38
2.67
2.66
2.64
2.63
2.62
2.60
2.16
2.14
2.13
1.88
1.86
1.84
1.71
1.37
1.07
Br
OH
O
H
H
1.00
1.12
1.01
1.03
1.07
1.03
1.12
3.01
3.15
3.00
3.19
f1 (ppm)

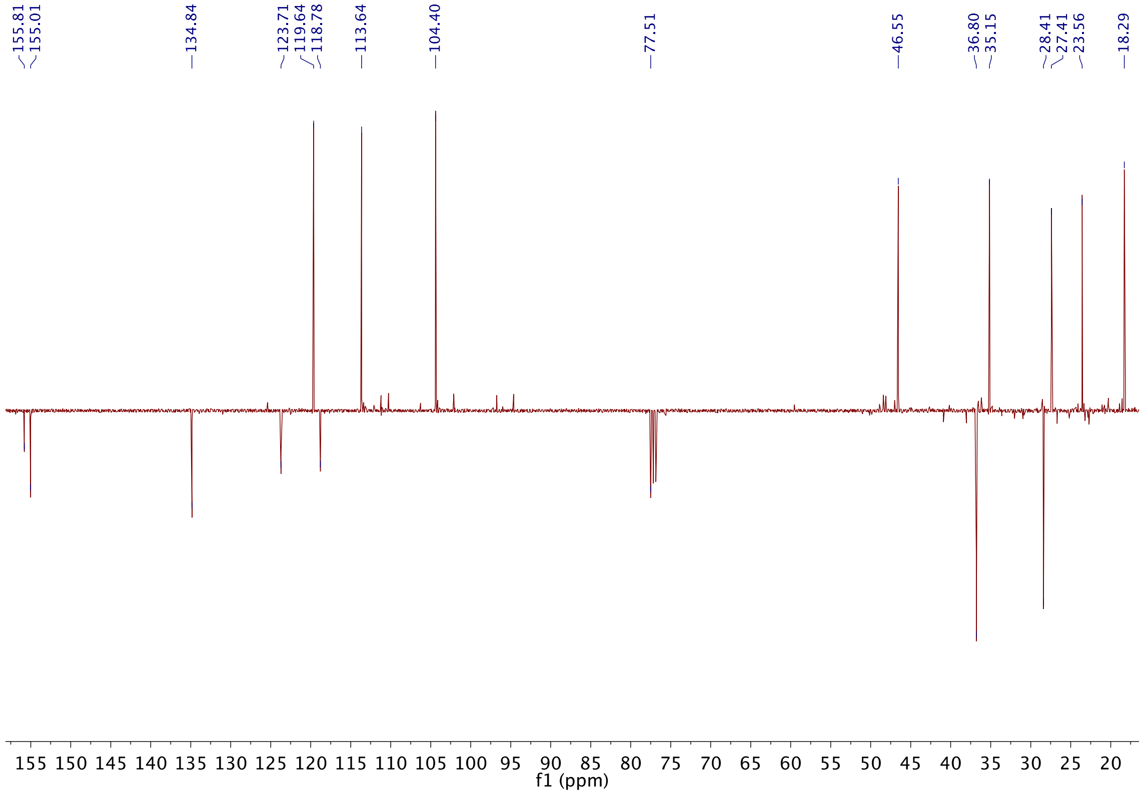
155.81
155.01
134.84
123.71
119.64
118.78
113.64
104.40
77.51
46.55
36.80
35.15
28.41
27.41
23.56
18.29
f1 (ppm)

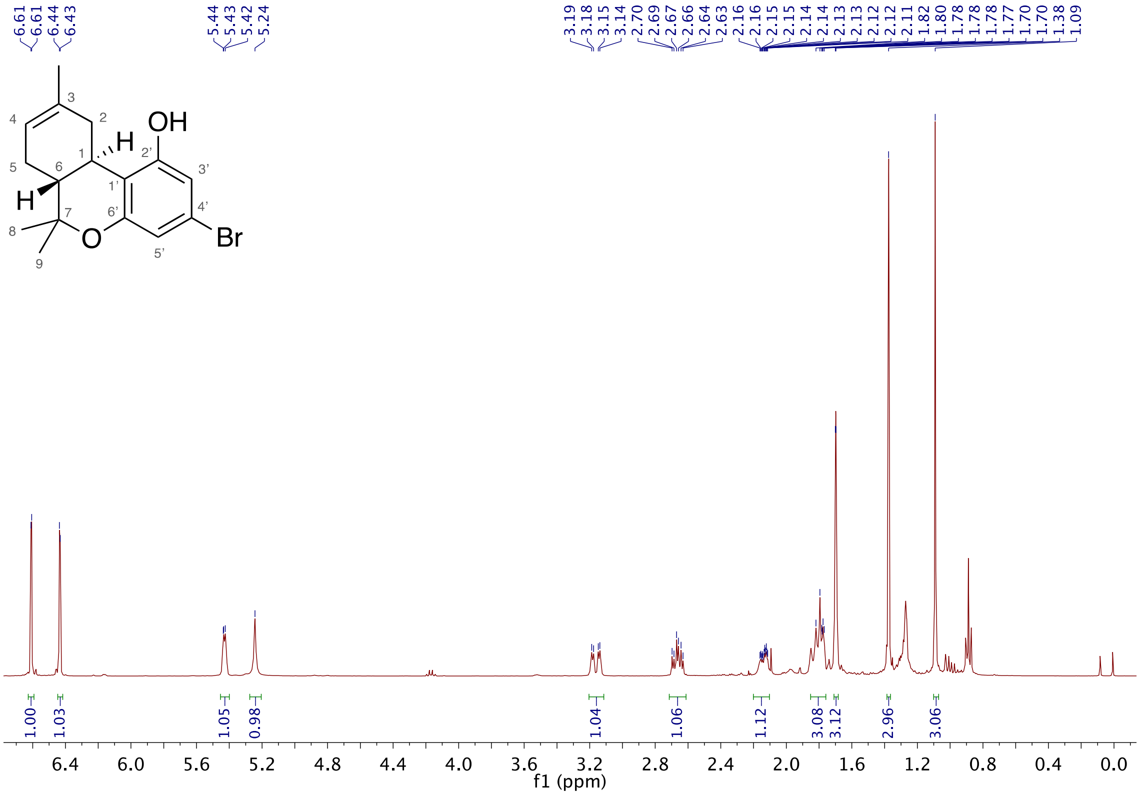
6.61
6.61
6.44
6.43
5.44
5.43
5.42
5.24
3.19
3.18
3.15
3.14
2.70
2.69
2.67
2.66
2.64
2.63
2.16
2.15
2.15
2.14
2.14
2.13
2.13
2.12
2.12
2.11
1.82
1.80
1.78
1.78
1.78
1.77
1.70
1.70
1.38
1.09
OH
H
H
O
Br
1
2
3
4
5
6
7
8
9
1'
2'
3'
4'
5'
6'
1.00
1.03
1.05
0.98
1.04
1.06
1.12
3.08
3.12
2.96
3.06
6.4
6.0
5.6
5.2
4.8
4.4
4.0
3.6
3.2
2.8
2.4
2.0
1.6
1.2
0.8
0.4
0.0
f1 (ppm)

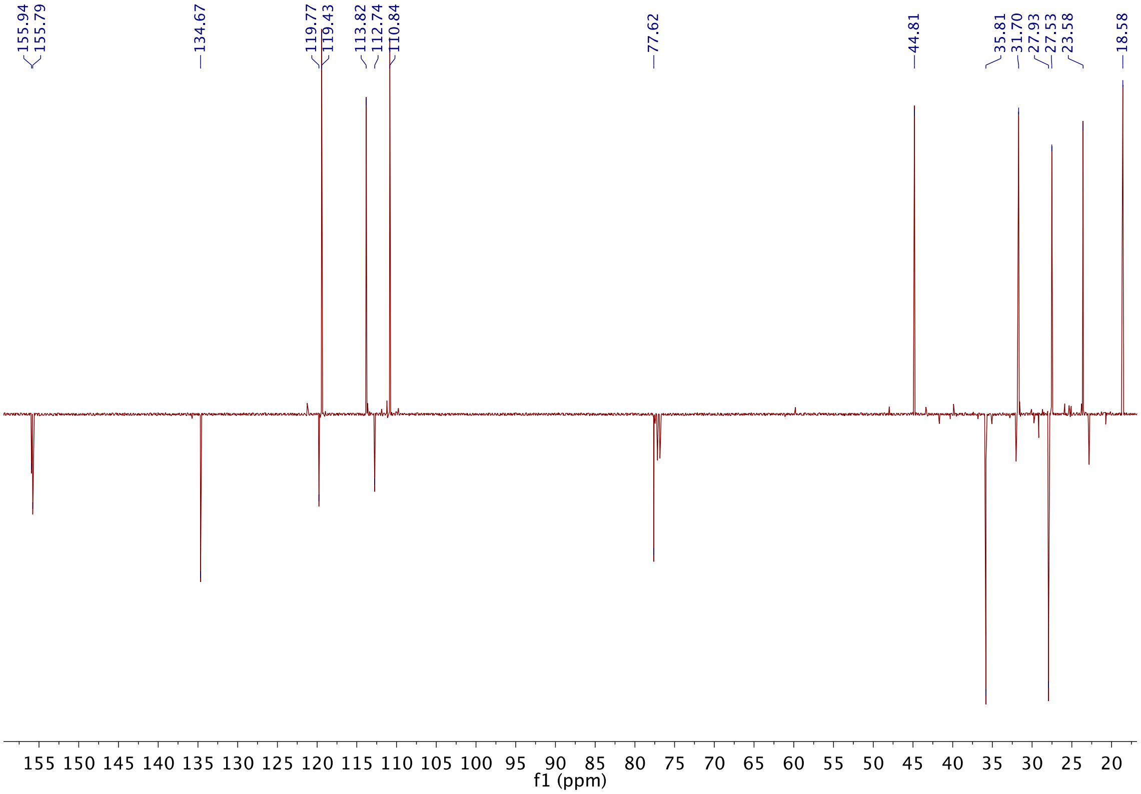
155.94
155.79
134.67
119.77
119.43
113.82
112.74
110.84
77.62
44.81
35.81
31.70
27.93
27.53
23.58
18.58
155 150 145 140 135 130 125 120 115 110 105 100 95 90 85 80 75 70 65 60 55 50 45 40 35 30 25 20
f1 (ppm)

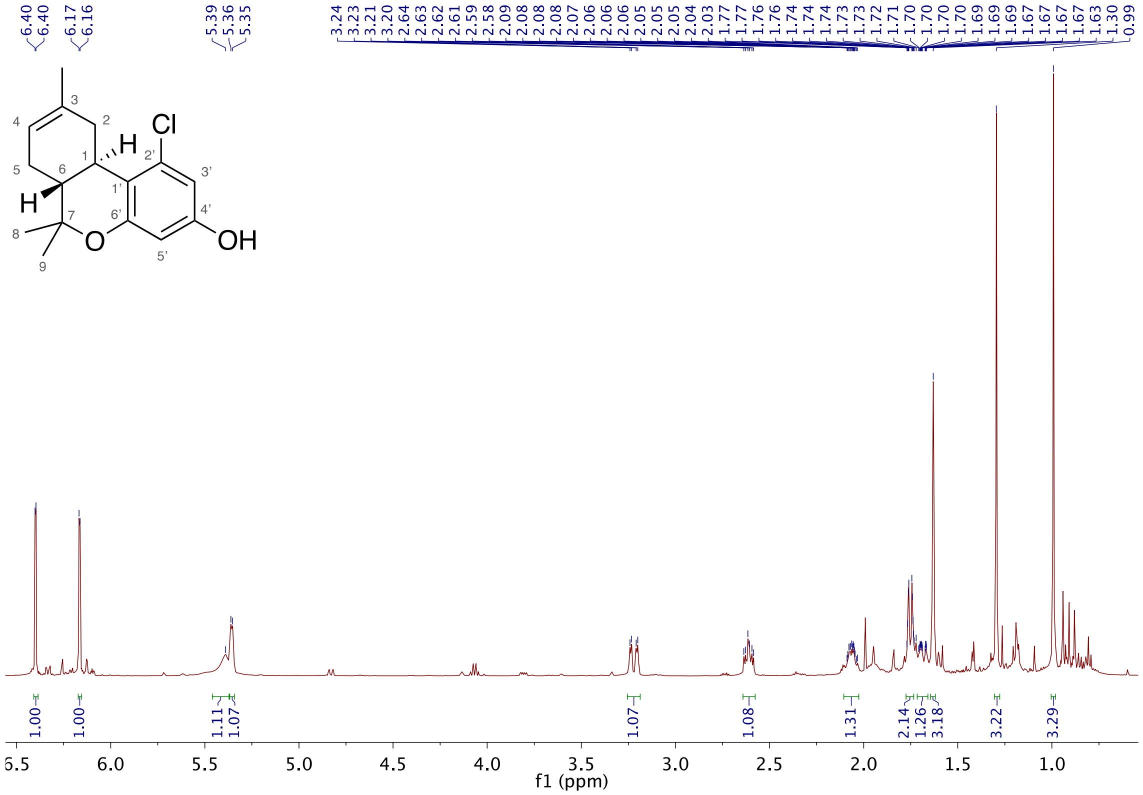
6.40 6.40 6.17 6.16 5.39 5.36 5.35 3.24 3.23 3.21 3.20 2.64 2.63 2.62 2.61 2.59 2.58 2.09 2.08 2.08 2.08 2.07 2.06 2.06 2.05 2.05 2.05 2.04 2.03 1.77 1.77 1.76 1.74 1.74 1.74 1.73 1.73 1.73 1.72 1.71 1.70 1.70 1.70 1.70 1.69 1.69 1.69 1.67 1.67 1.67 1.67 1.63 1.30 0.99
Cl
H
H
O
OH
1.00 1.00 1.11 1.07 1.07 1.08 1.31 2.14 1.26 3.18 3.22 3.29
f1 (ppm)

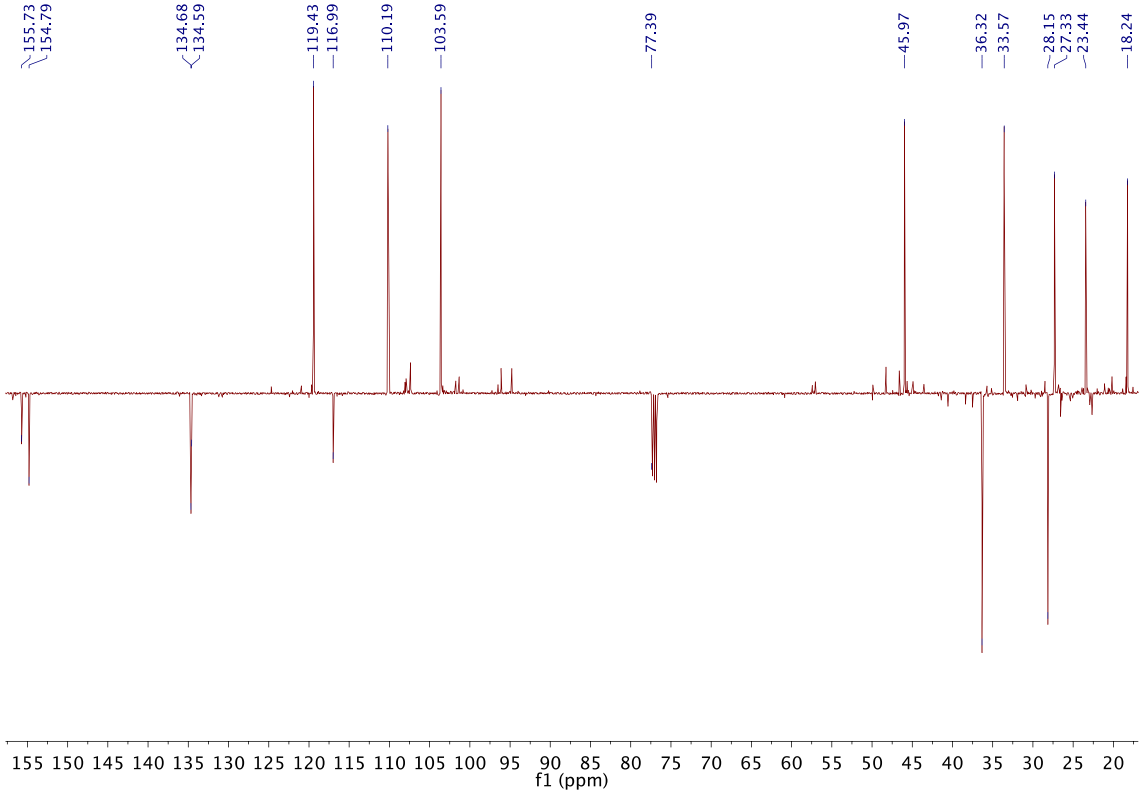
155.73 154.79 134.68 134.59 119.43 116.99 110.19 103.59 77.39 45.97 36.32 33.57 28.15 27.33 23.44 18.24
f1 (ppm)

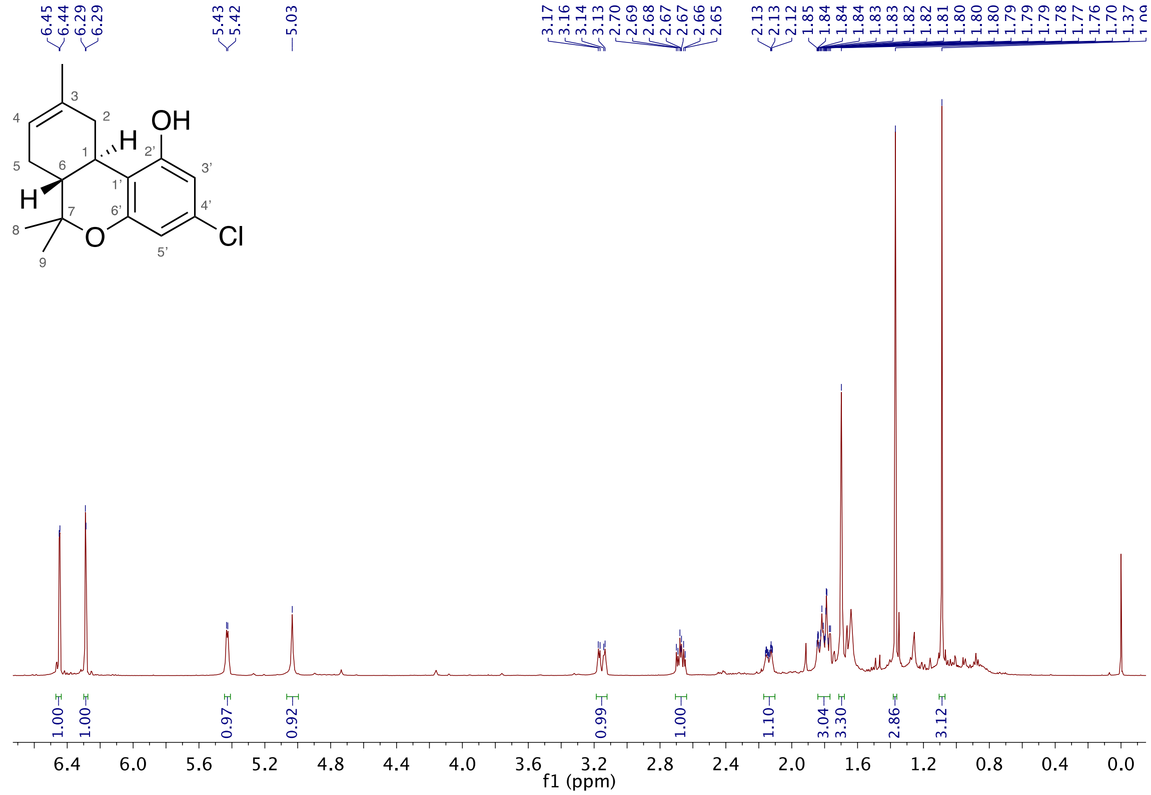
6.45
6.44
6.29
6.29
5.43
5.42
5.03
3.17
3.16
3.14
3.13
2.70
2.69
2.68
2.67
2.67
2.66
2.65
2.13
2.13
2.12
1.85
1.84
1.84
1.84
1.83
1.83
1.82
1.82
1.81
1.80
1.80
1.80
1.79
1.79
1.79
1.78
1.77
1.76
1.70
1.37
OH
H
H
O
Cl
1.00
1.00
0.97
0.92
0.99
1.00
1.10
3.04
3.30
2.86
3.12
6.4 6.0 5.6 5.2 4.8 4.4 4.0 3.6 3.2 2.8 2.4 2.0 1.6 1.2 0.8 0.4 0.0
f1 (ppm)

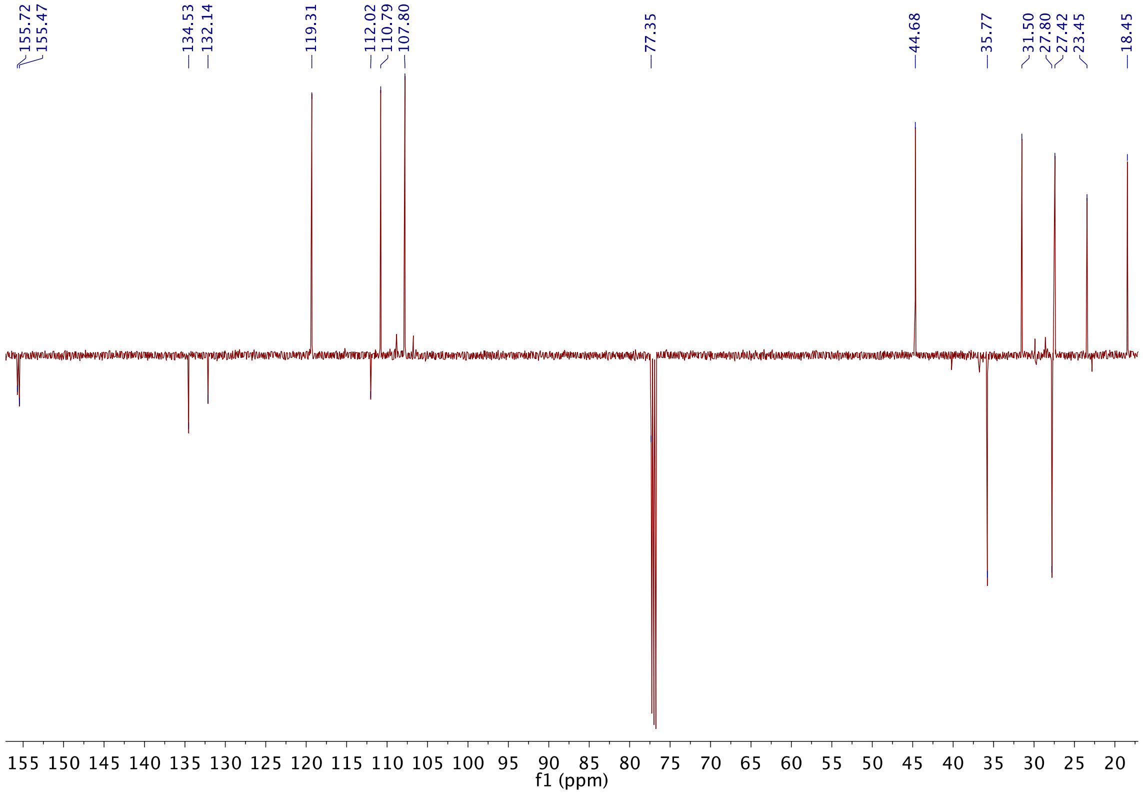
155.72
155.47
134.53
132.14
119.31
112.02
110.79
107.80
77.35
44.68
35.77
31.50
27.80
27.42
23.45
18.45
155 150 145 140 135 130 125 120 115 110 105 100 95 90 85 80 75 70 65 60 55 50 45 40 35 30 25 20
f1 (ppm)

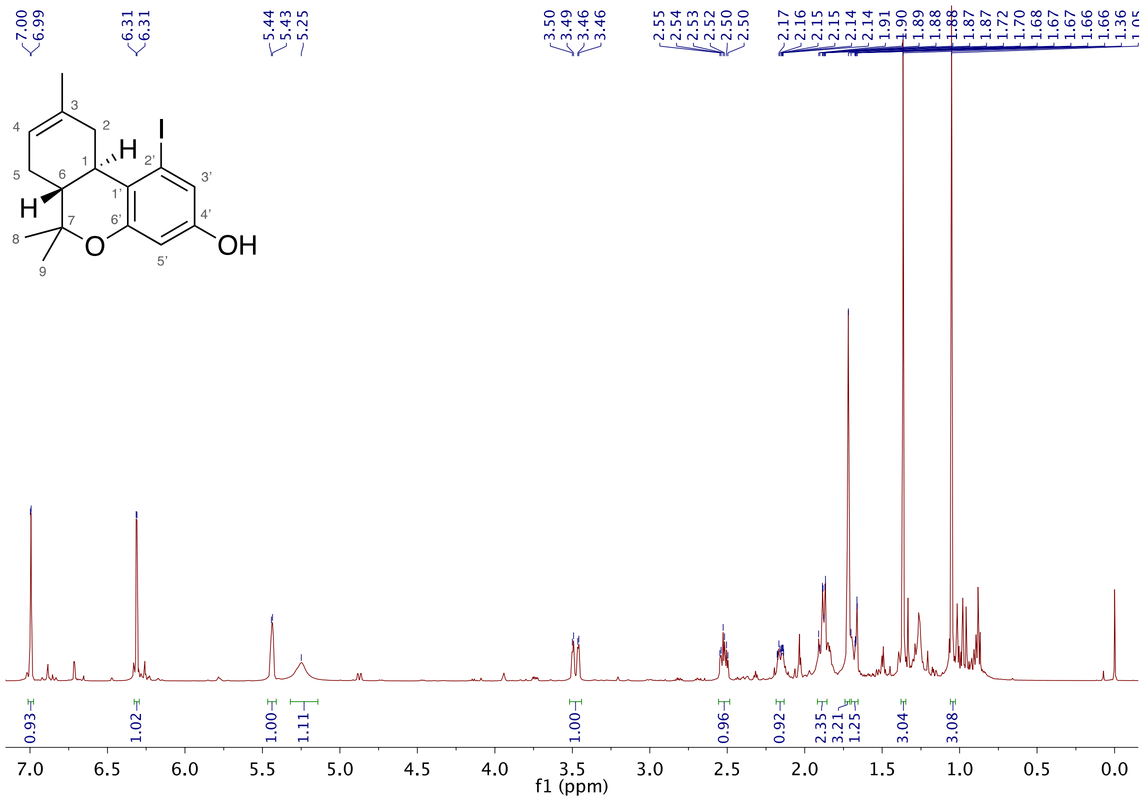
7.00
6.99
6.31
6.31
5.44
5.43
5.25
3.50
3.49
3.46
3.46
2.55
2.54
2.53
2.52
2.50
2.50
2.17
2.16
2.15
2.15
2.14
2.14
1.91
1.90
1.89
1.88
1.88
1.87
1.87
1.72
1.70
1.68
1.67
1.67
1.66
1.66
1.36
OH
0.93
1.02
1.00
1.11
1.00
0.96
0.92
2.35
3.21
1.25
3.04
3.08
7.0 6.5 6.0 5.5 5.0 4.5 4.0 3.5 3.0 2.5 2.0 1.5 1.0 0.5 0.0
f1 (ppm)

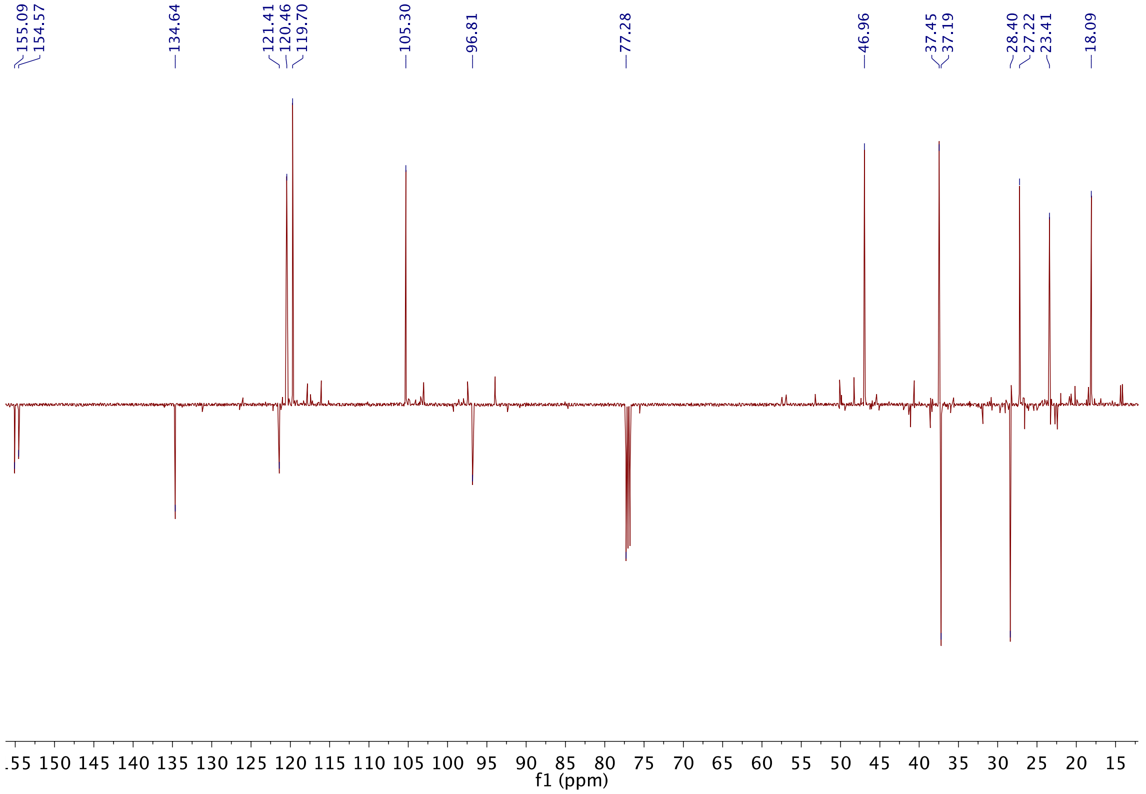
155.09
154.57
134.64
121.41
120.46
119.70
105.30
96.81
77.28
46.96
37.45
37.19
28.40
27.22
23.41
18.09
.55 150 145 140 135 130 125 120 115 110 105 100 95 90 85 80 75 70 65 60 55 50 45 40 35 30 25 20 15
f1 (ppm)

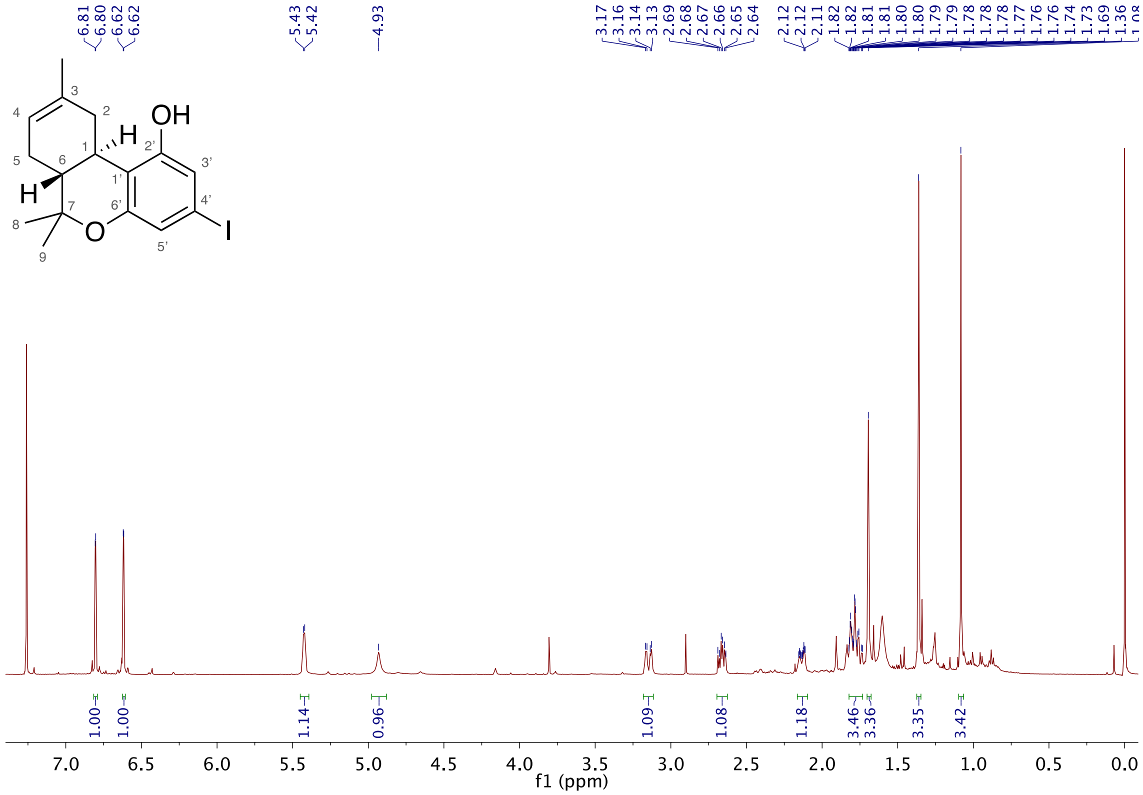

6.81
6.80
6.62
6.62
5.43
5.42
4.93
3.17
3.16
3.14
3.13
2.69
2.68
2.67
2.66
2.65
2.64
2.12
2.12
2.11
1.82
1.82
1.81
1.81
1.80
1.80
1.79
1.79
1.78
1.78
1.78
1.77
1.76
1.74
1.73
1.69
1.36
OH
H
H
O
I
1
2
3
4
5
6
7
8
9
1'
2'
3'
4'
5'
6'
1.00
1.00
1.14
0.96
1.09
1.08
1.18
3.46
3.36
3.35
3.42
7.0 6.5 6.0 5.5 5.0 4.5 4.0 3.5 3.0 2.5 2.0 1.5 1.0 0.5 0.0
f1 (ppm)

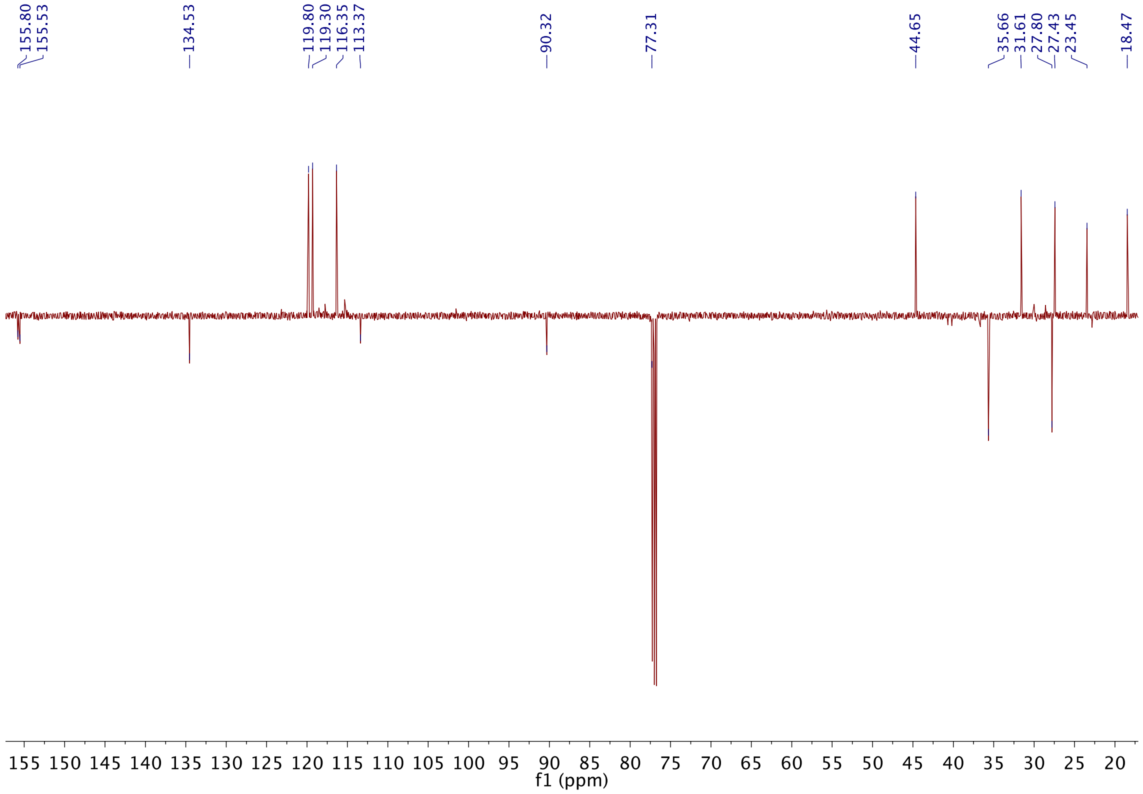

155.80
155.53
134.53
119.80
119.30
116.35
113.37
90.32
77.31
44.65
35.66
31.61
27.80
27.43
23.45
18.47
155 150 145 140 135 130 125 120 115 110 105 100 95 90 85 80 75 70 65 60 55 50 45 40 35 30 25 20
f1 (ppm)

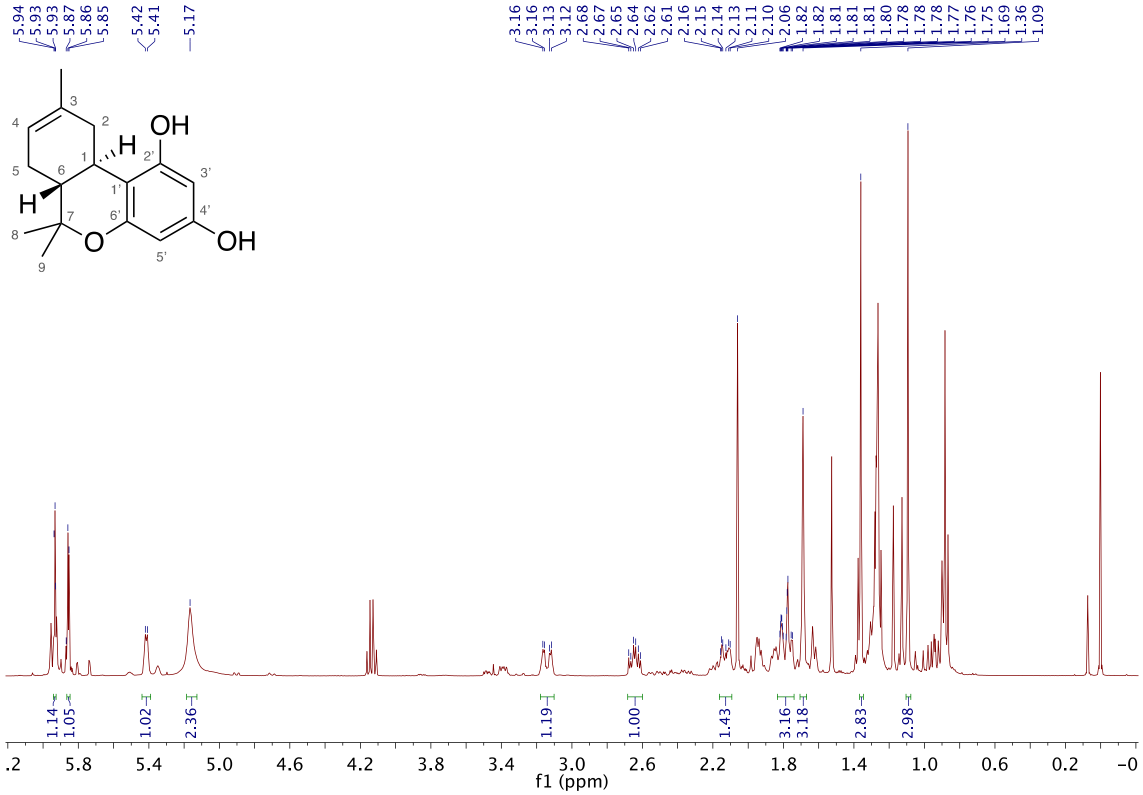
5.94
5.93
5.93
5.87
5.86
5.85
5.42
5.41
5.17
3.16
3.16
3.13
3.12
2.68
2.67
2.65
2.64
2.62
2.61
2.16
2.15
2.14
2.13
2.11
2.10
2.06
1.82
1.82
1.81
1.81
1.81
1.80
1.78
1.78
1.77
1.76
1.75
1.69
1.36
1.09
OH
OH
H
H
O
1.14
1.05
1.02
2.36
1.19
1.00
1.43
3.16
3.18
2.83
2.98
f1 (ppm)

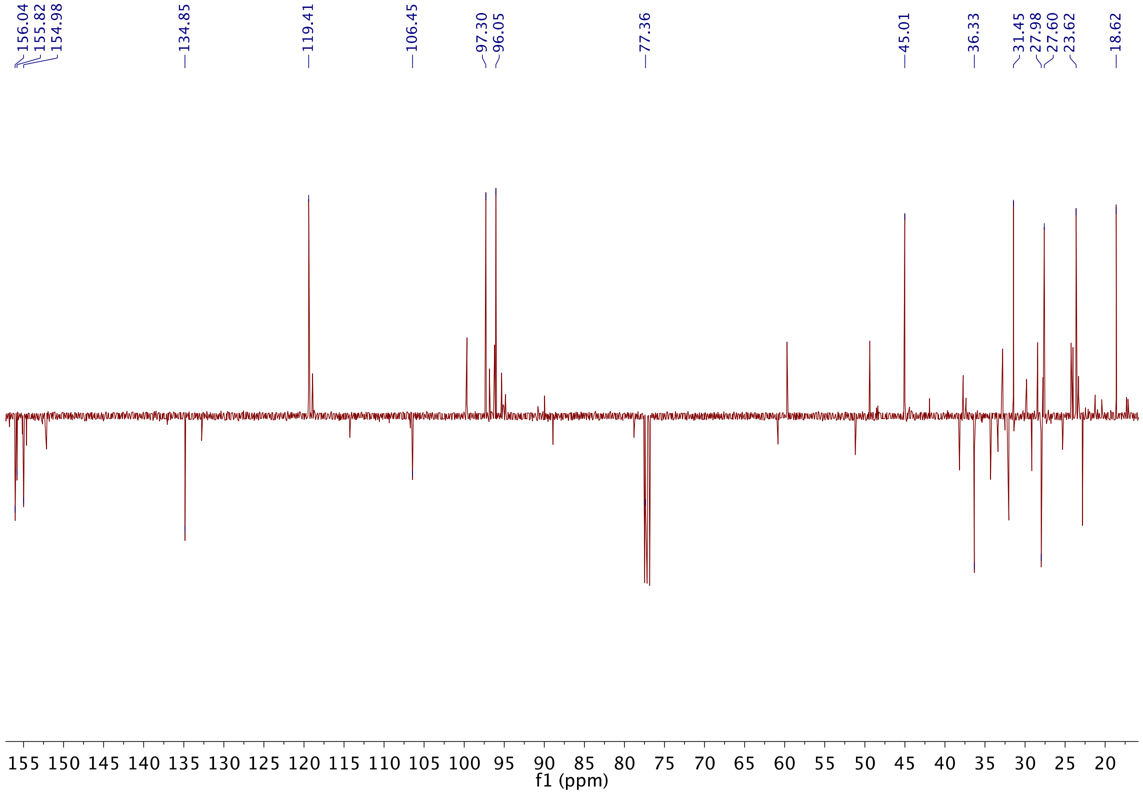
156.04
155.82
154.98
134.85
119.41
106.45
97.30
96.05
77.36
45.01
36.33
31.45
27.98
27.60
23.62
18.62
f1 (ppm)

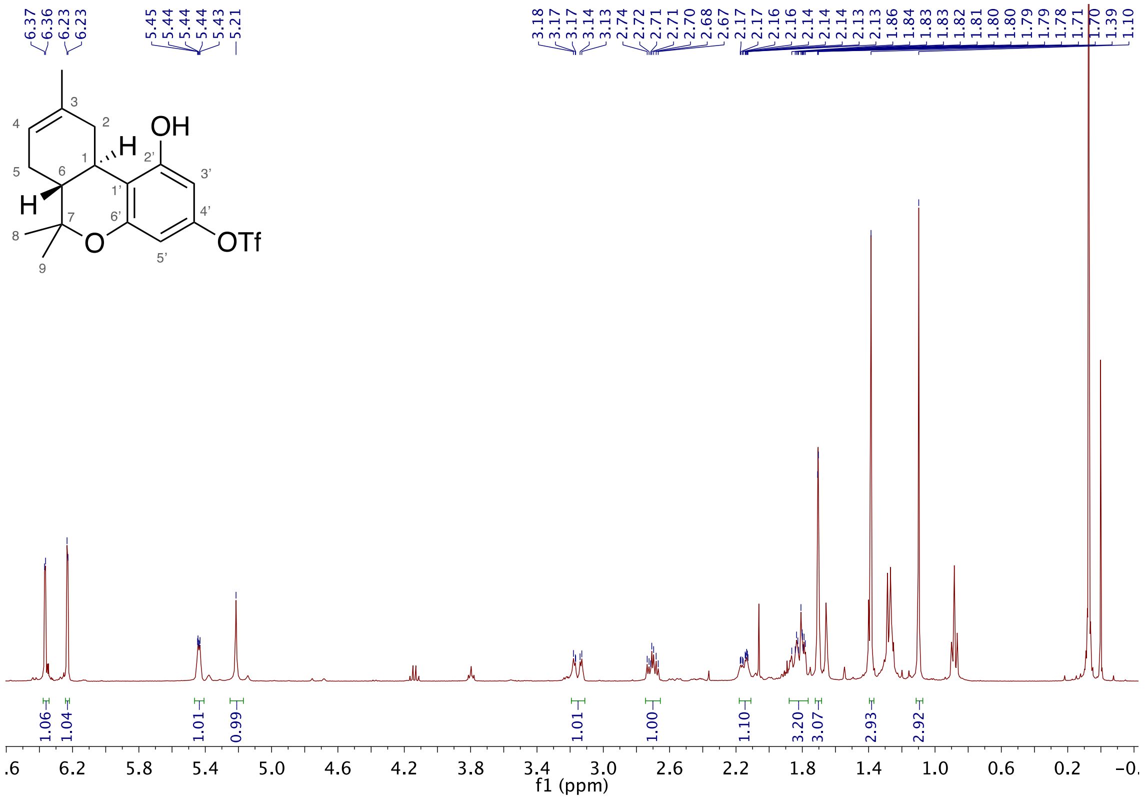
OH
OTf
f1 (ppm)

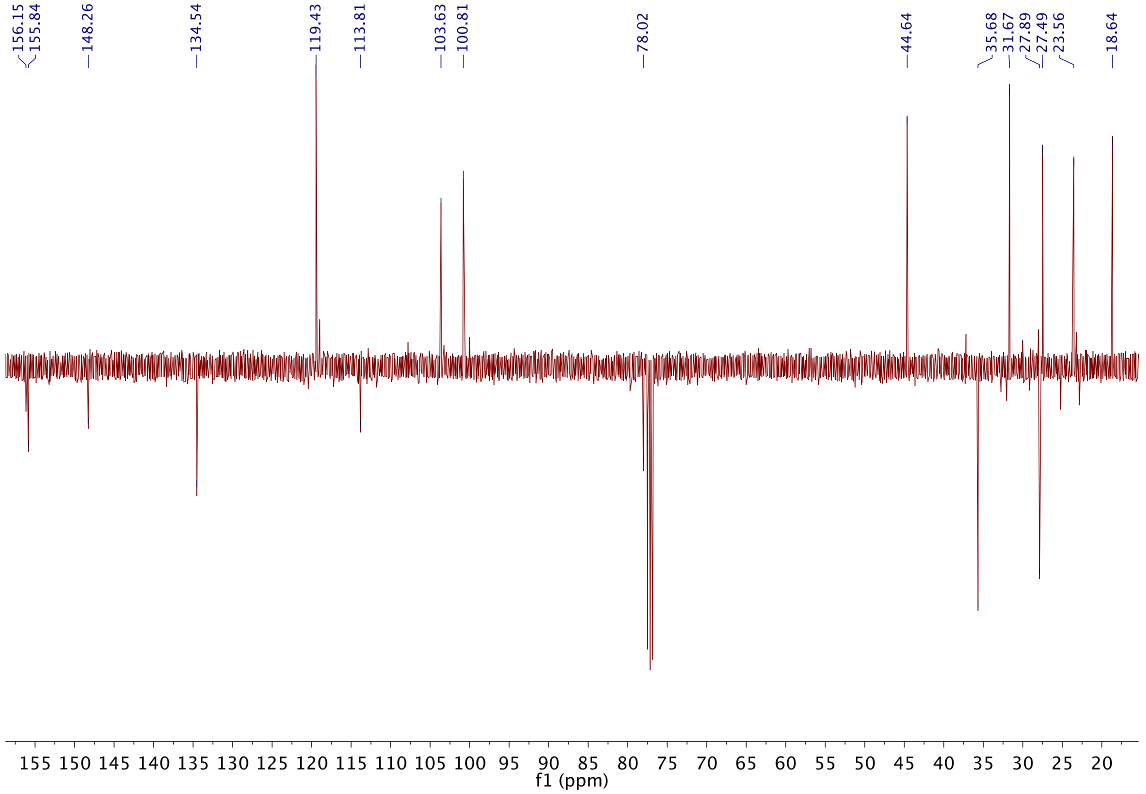
f1 (ppm)

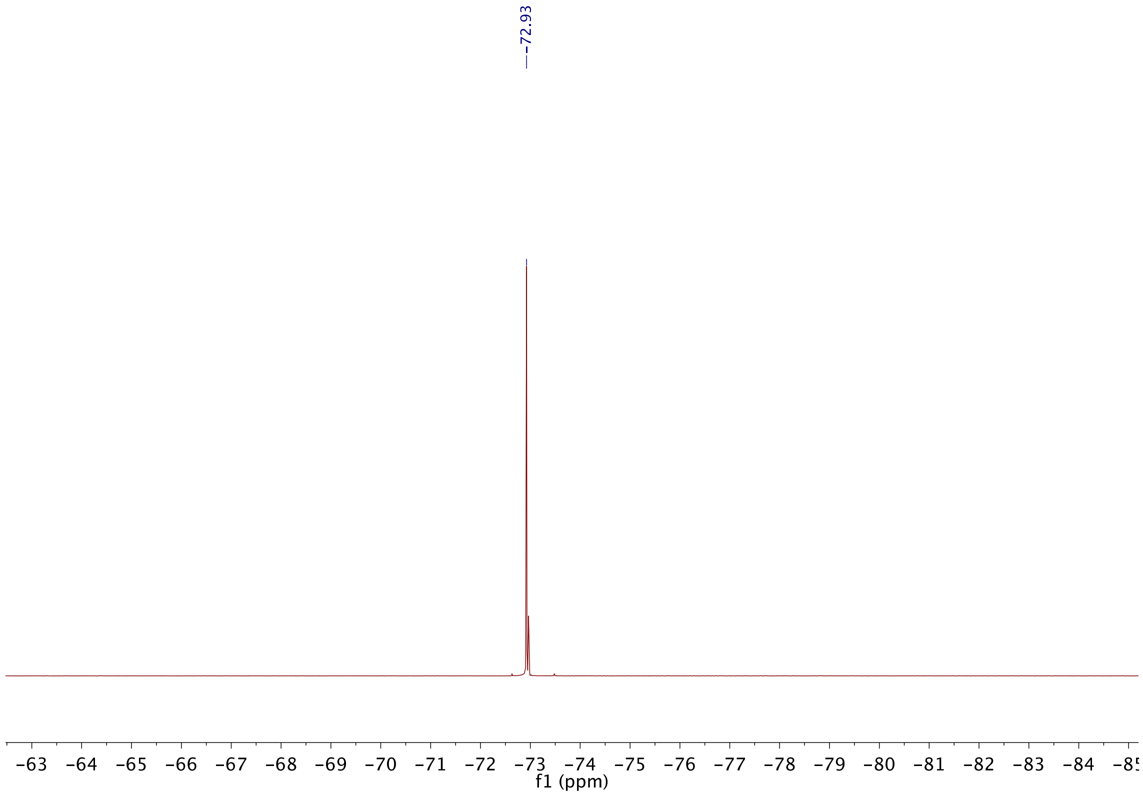

−72.93
−63 −64 −65 −66 −67 −68 −69 −70 −71 −72 −73 −74 −75 −76 −77 −78 −79 −80 −81 −82 −83 −84
f1 (ppm)

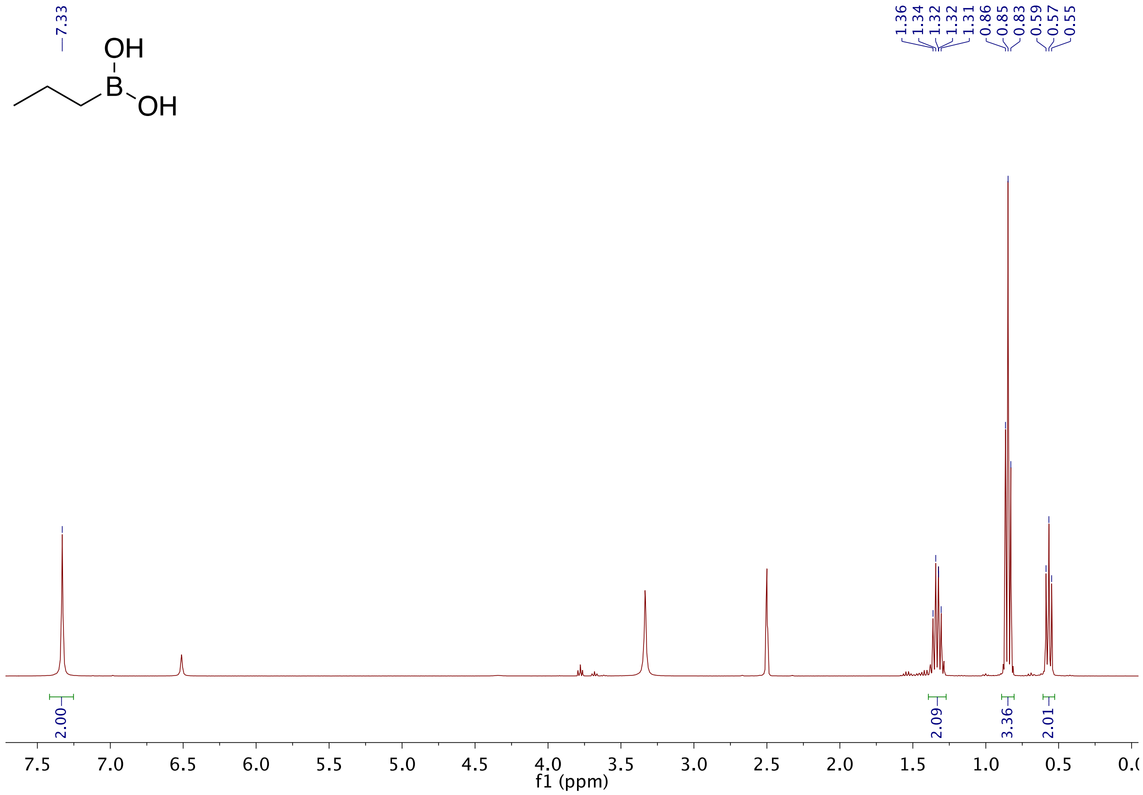
7.33
OH
B
OH
1.36
1.34
1.32
1.32
1.31
0.86
0.85
0.83
0.59
0.57
0.55
2.00
2.09
3.36
2.01
7.5 7.0 6.5 6.0 5.5 5.0 4.5 4.0 3.5 3.0 2.5 2.0 1.5 1.0 0.5
f1 (ppm)

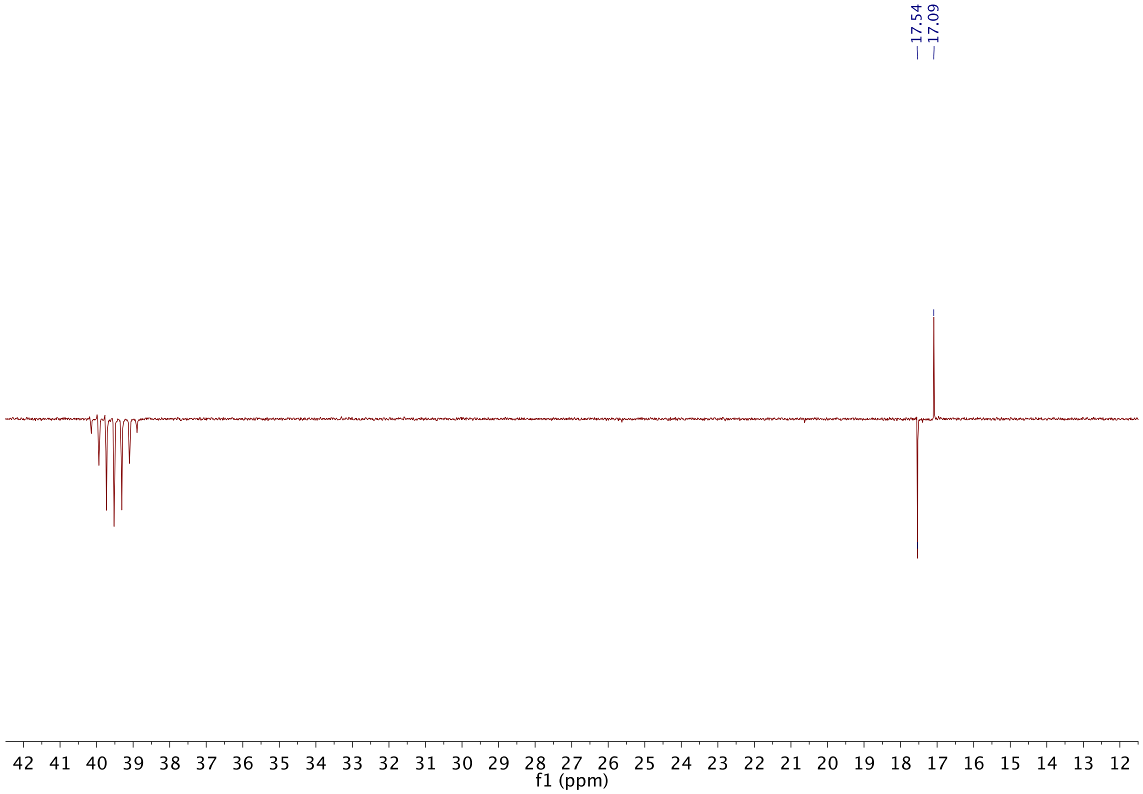
17.54
17.09
42 41 40 39 38 37 36 35 34 33 32 31 30 29 28 27 26 25 24 23 22 21 20 19 18 17 16 15 14 13 12
f1 (ppm)

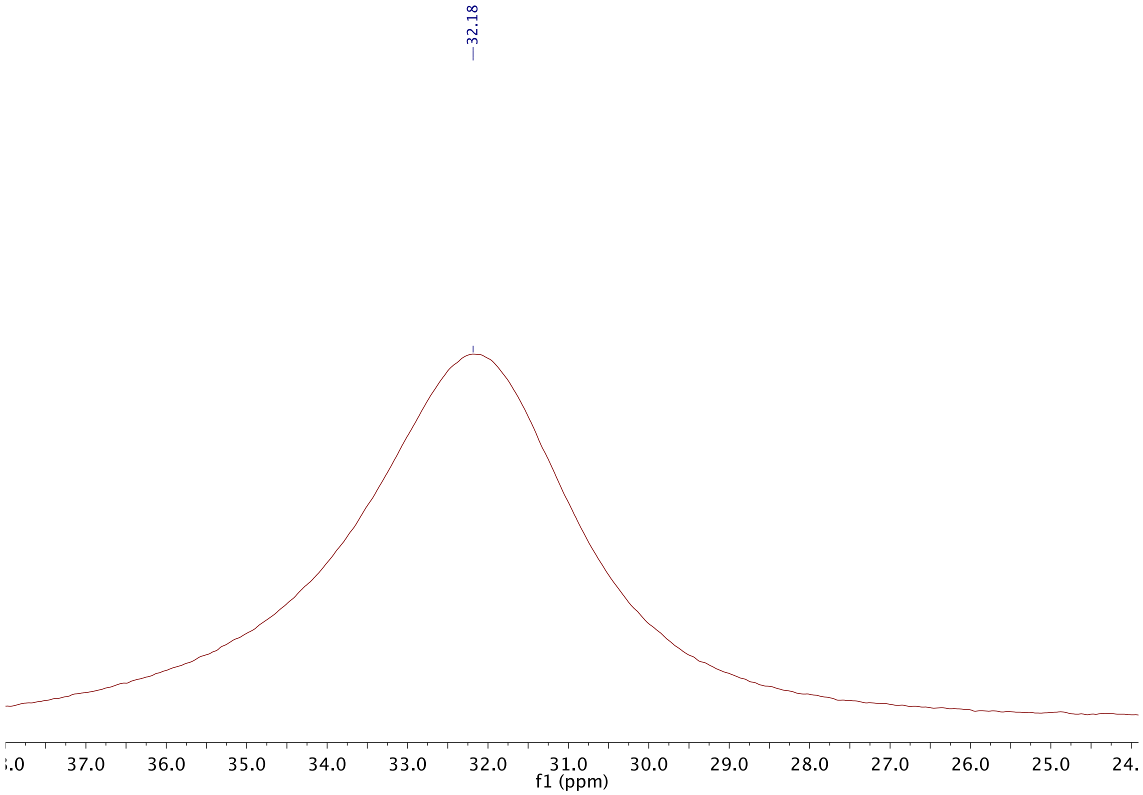

32.18
.0
37.0
36.0
35.0
34.0
33.0
32.0
31.0
30.0
29.0
28.0
27.0
26.0
25.0
24.
f1 (ppm)

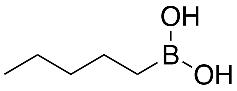
OH
B
OH

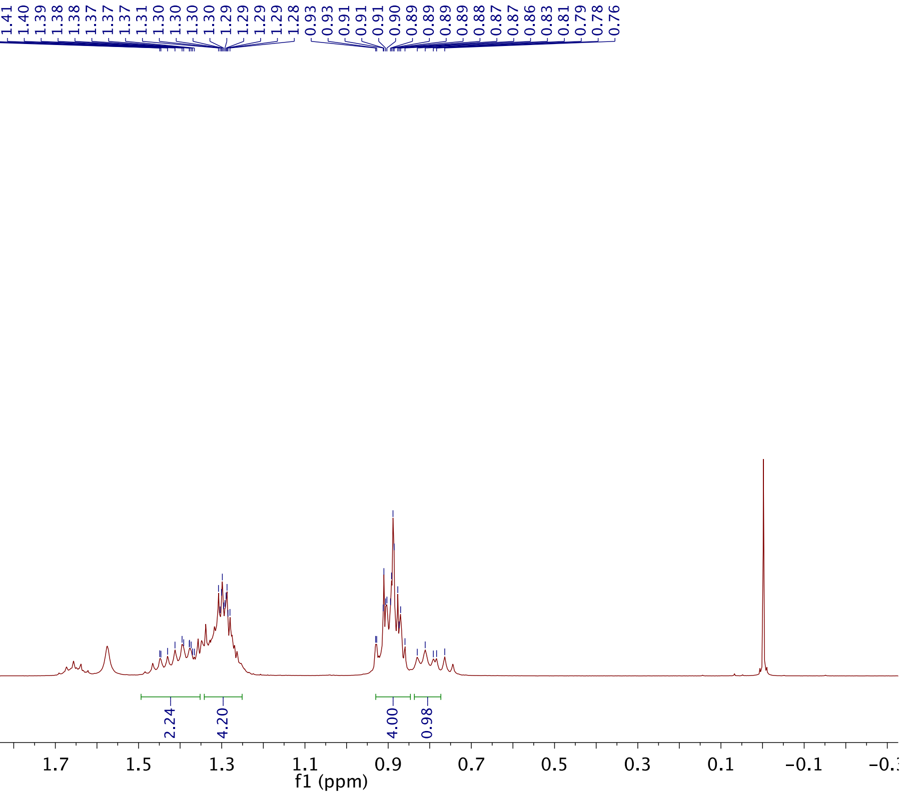
1.45 1.45 1.43 1.41 1.40 1.39 1.38 1.38 1.37 1.37 1.37 1.31 1.30 1.30 1.30 1.30 1.29 1.29 1.29 1.29 1.28 0.93 0.93 0.91 0.91 0.91 0.90 0.89 0.89 0.89 0.89 0.88 0.87 0.87 0.86 0.83 0.81 0.79 0.78 0.76
2.24
4.20
4.00
0.98
2.3 2.1 1.9 1.7 1.5 1.3 1.1 0.9 0.7 0.5 0.3 0.1 −0.1
f1 (ppm)

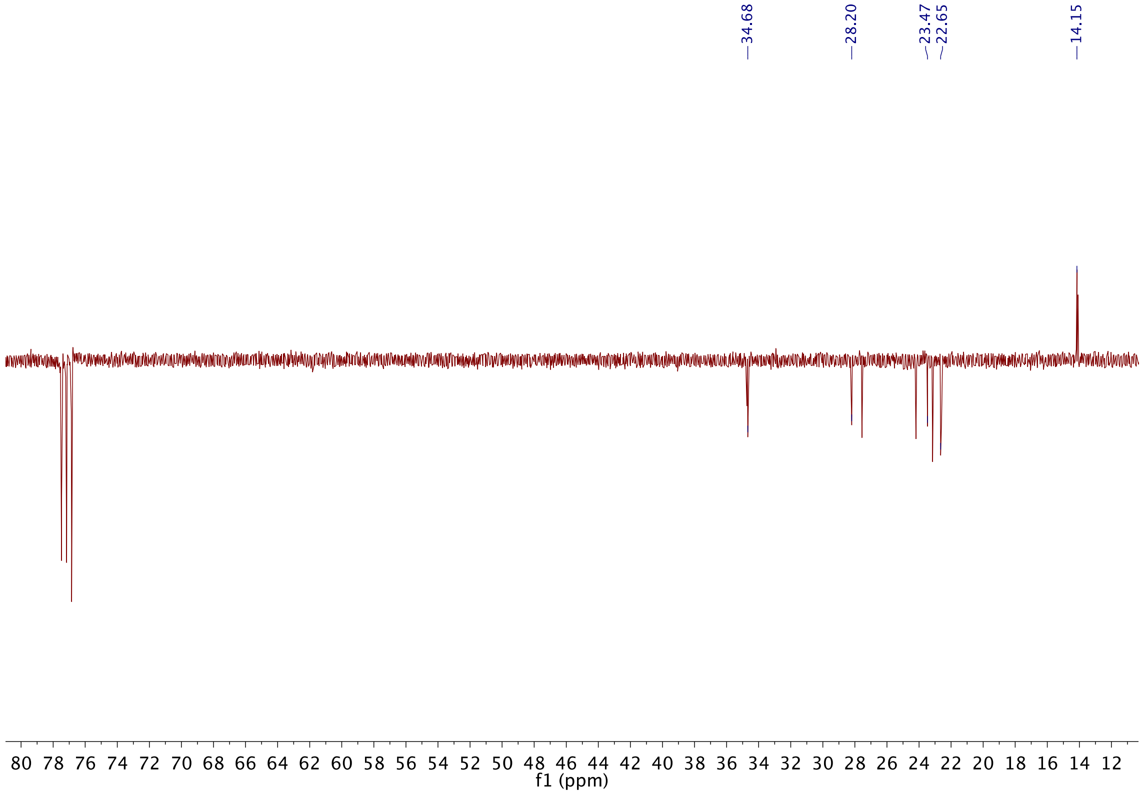
34.68
28.20
23.47
22.65
14.15
80 78 76 74 72 70 68 66 64 62 60 58 56 54 52 50 48 46 44 42 40 38 36 34 32 30 28 26 24 22 20 18 16 14 12
f1 (ppm)

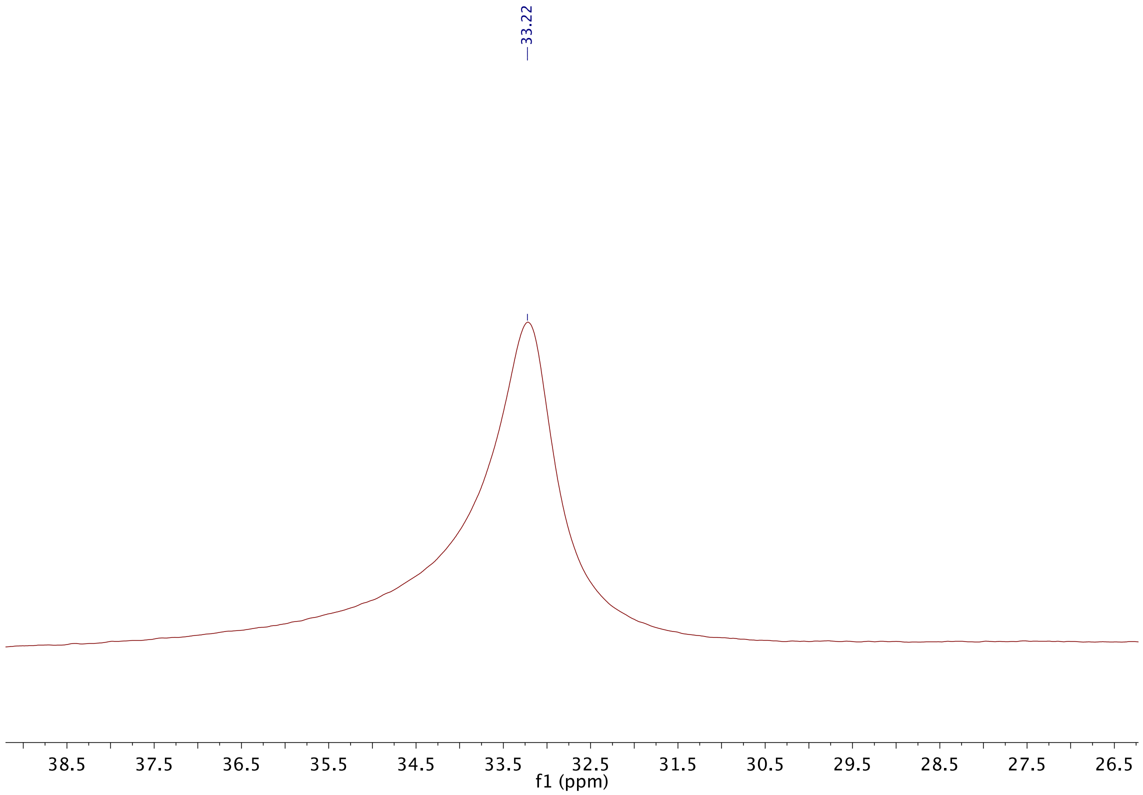
33.22
38.5
37.5
36.5
35.5
34.5
33.5
32.5
31.5
30.5
29.5
28.5
27.5
26.5
f1 (ppm)

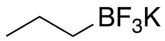
BF3K

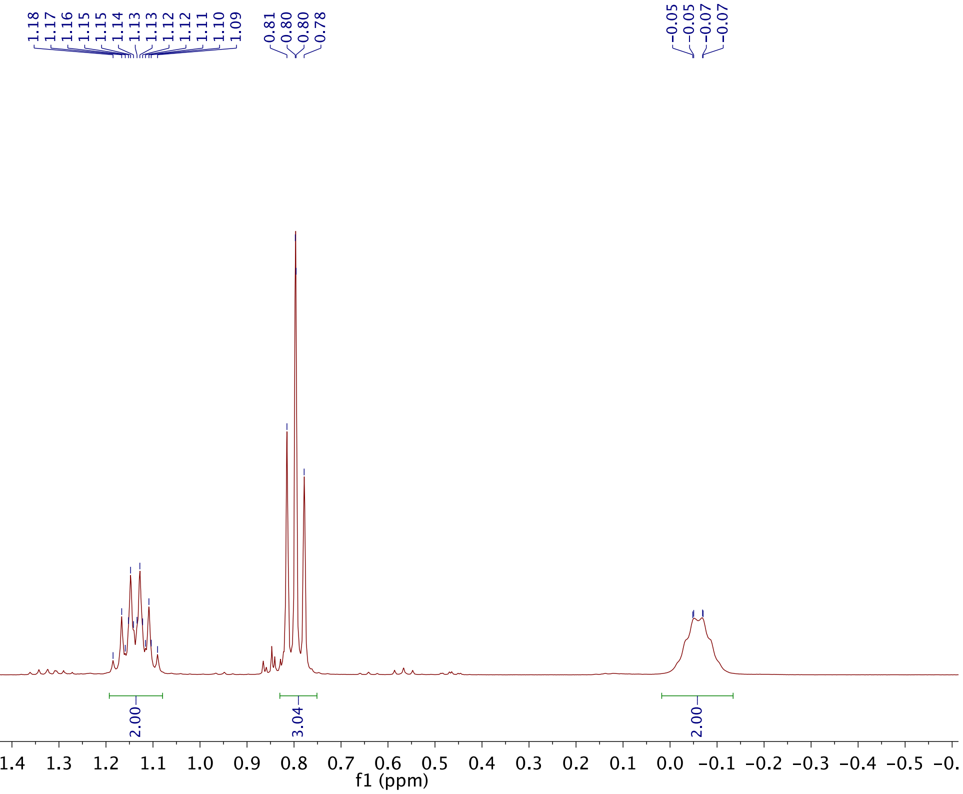
1.18
1.17
1.16
1.15
1.15
1.14
1.13
1.13
1.12
1.12
1.11
1.10
1.09
0.81
0.80
0.80
0.78
−0.05
−0.05
−0.07
−0.07
2.00
3.04
2.00
1.7 1.6 1.5 1.4 1.3 1.2 1.1 1.0 0.9 0.8 0.7 0.6 0.5 0.4 0.3 0.2 0.1 0.0 −0.1 −0.2 −0.3 −0.4 −0.5 −0.
f1 (ppm)

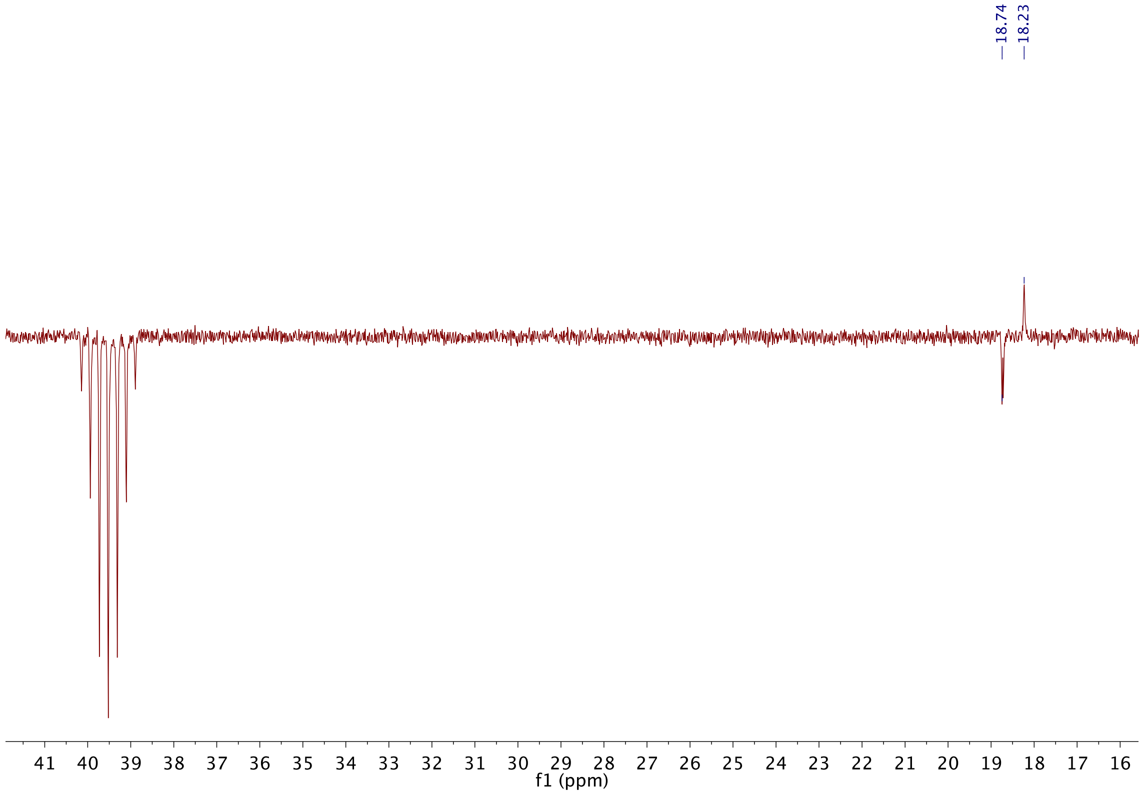
18.74
18.23
41 40 39 38 37 36 35 34 33 32 31 30 29 28 27 26 25 24 23 22 21 20 19 18 17 16
f1 (ppm)

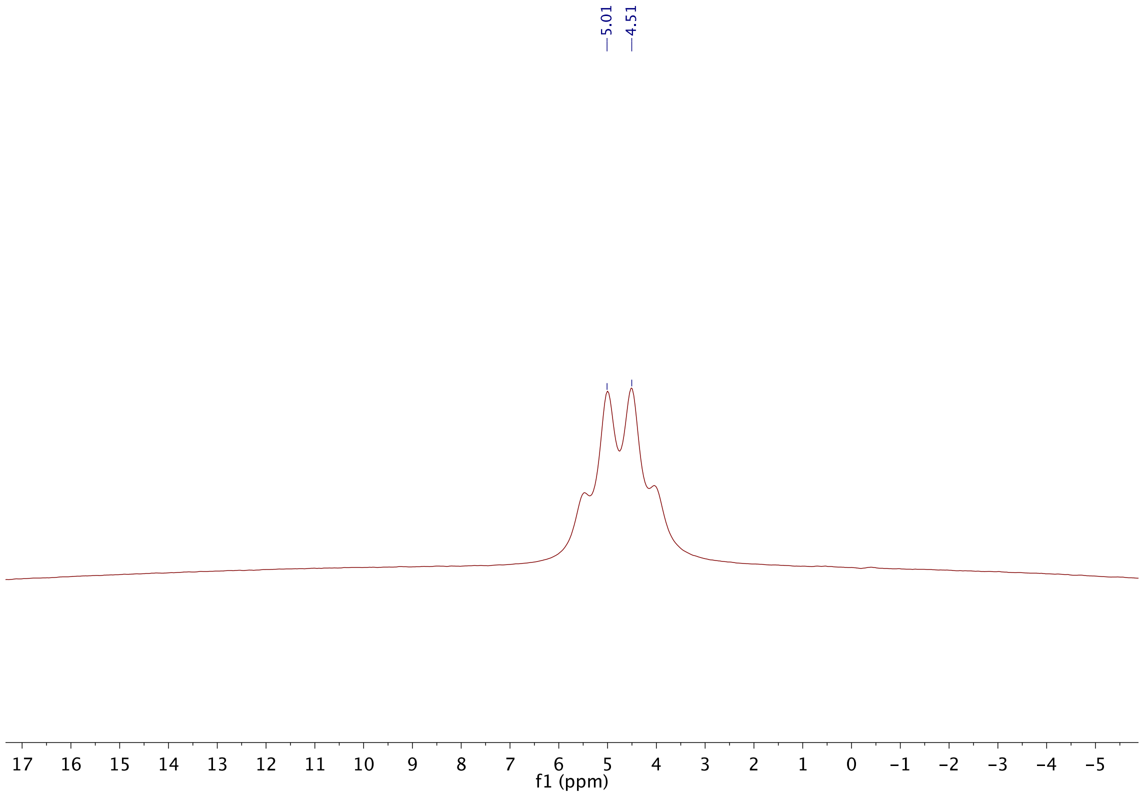
5.01
4.51
17 16 15 14 13 12 11 10 9 8 7 6 5 4 3 2 1 0 −1 −2 −3 −4 −5
f1 (ppm)

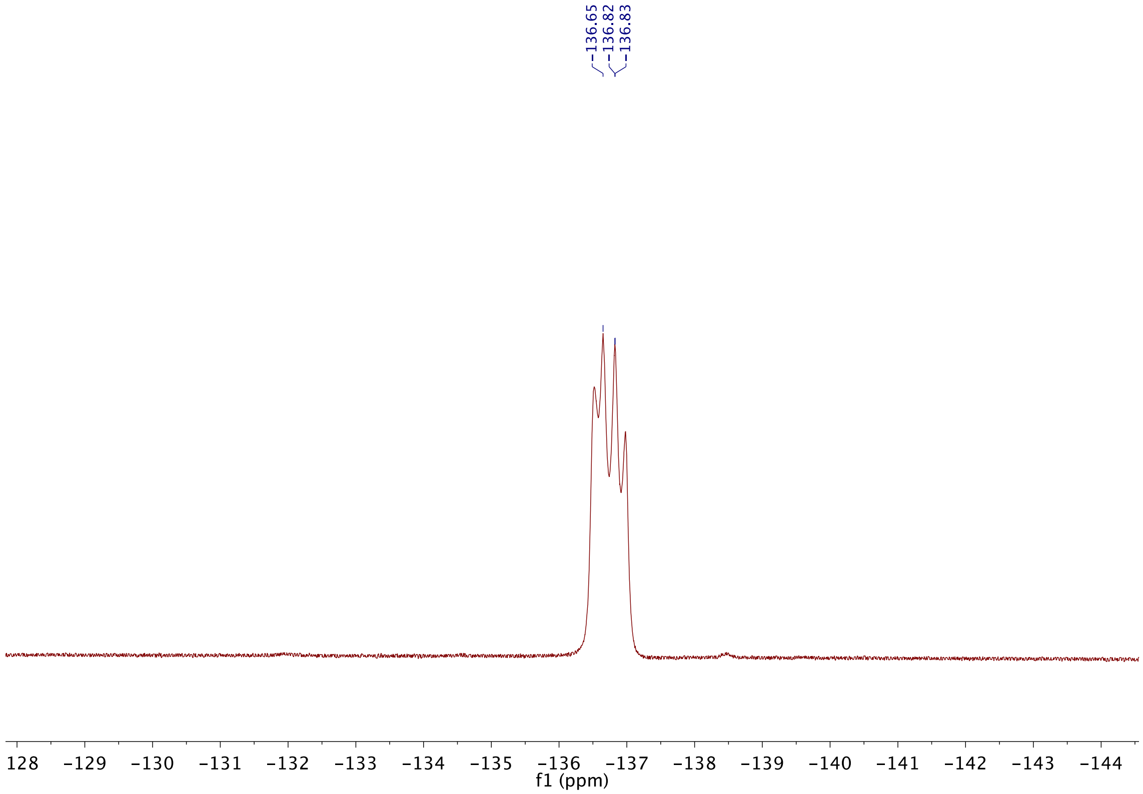
−136.65
−136.82
−136.83
128 −129 −130 −131 −132 −133 −134 −135 −136 −137 −138 −139 −140 −141 −142 −143 −144
f1 (ppm)

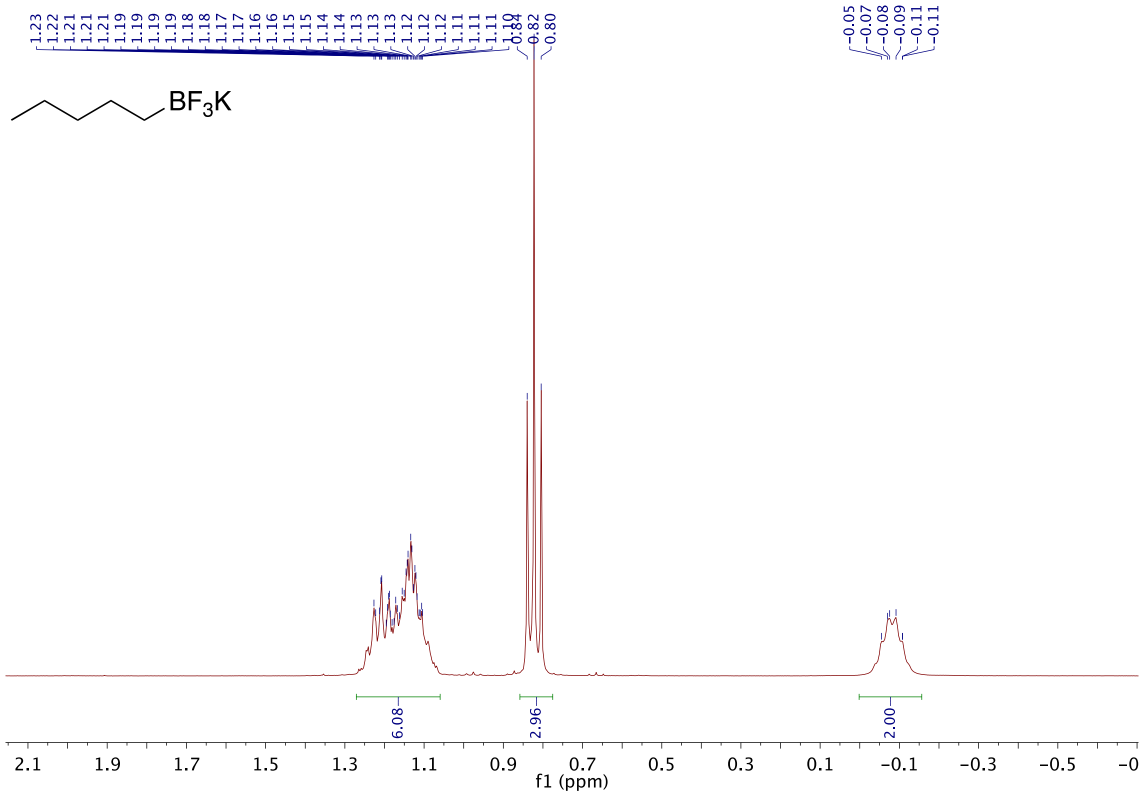
BF3K
6.08
2.96
2.00
f1 (ppm)

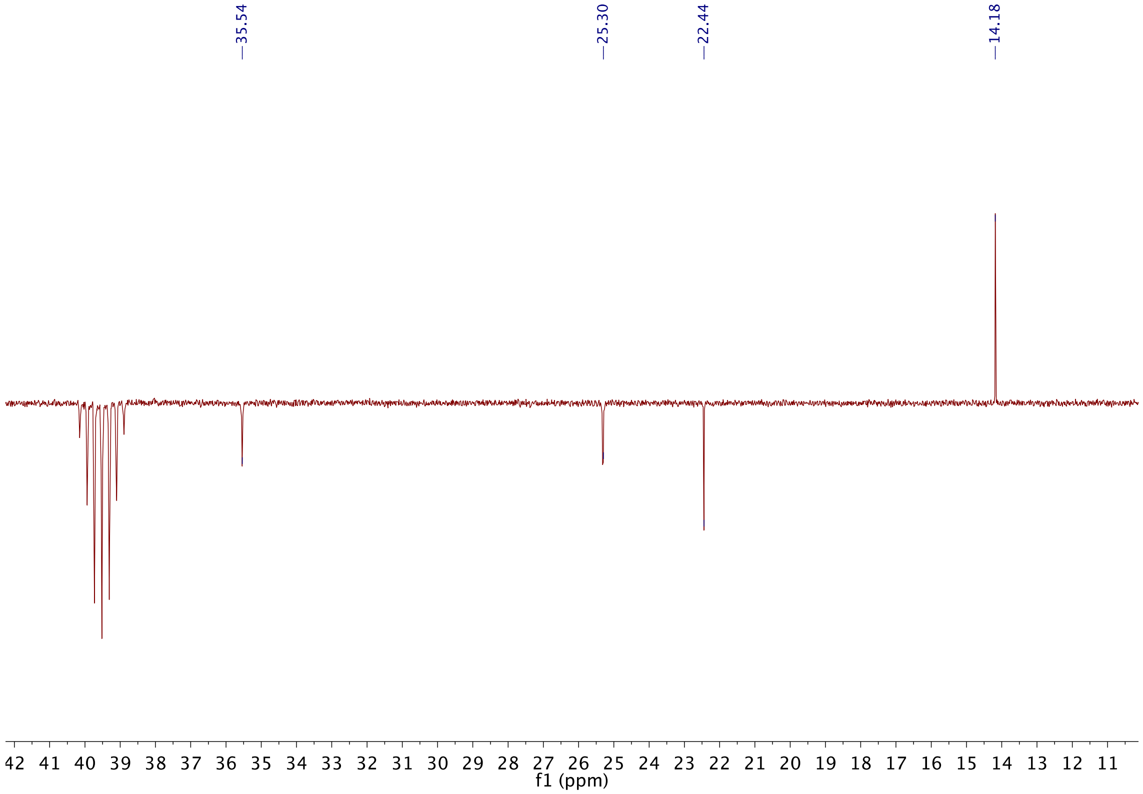
35.54
25.30
22.44
14.18
f1 (ppm)

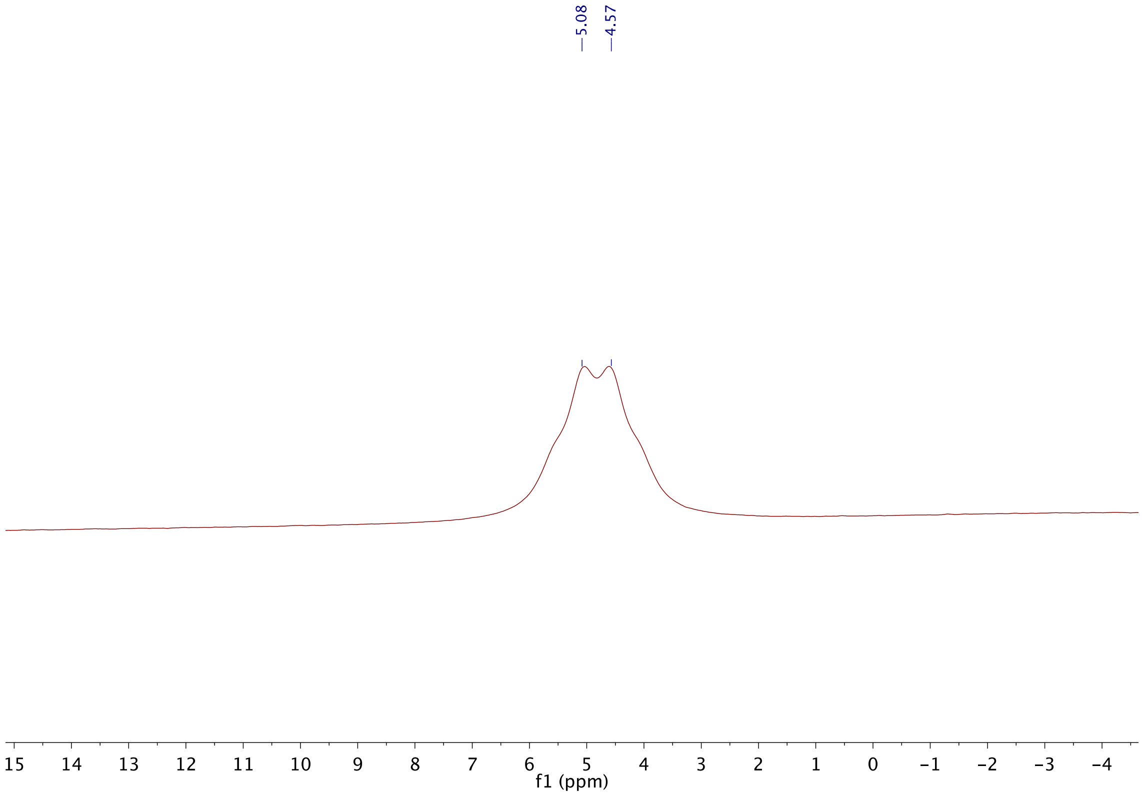

5.08
4.57
15
14
13
12
11
10
9
8
7
6
5
4
3
2
1
0
−1
−2
−3
−4
f1 (ppm)

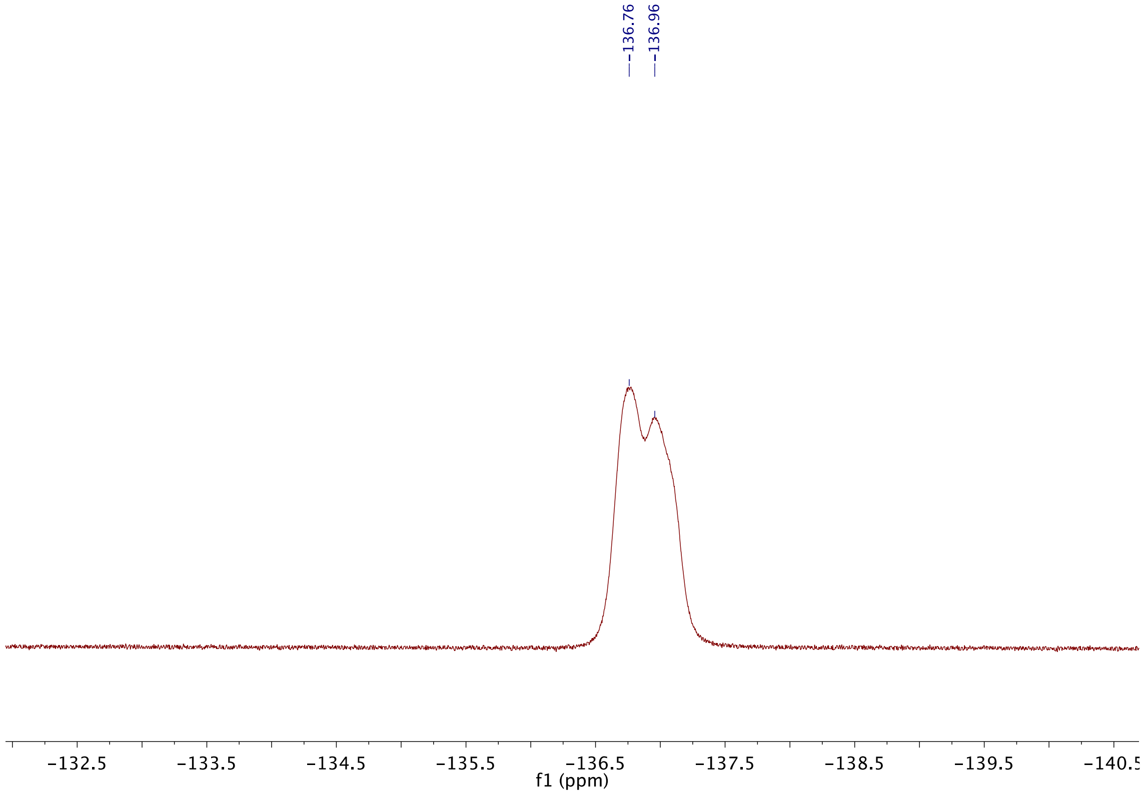

−136.76
−136.96
−132.5
−133.5
−134.5
−135.5
−136.5
−137.5
−138.5
−139.5
f1 (ppm)

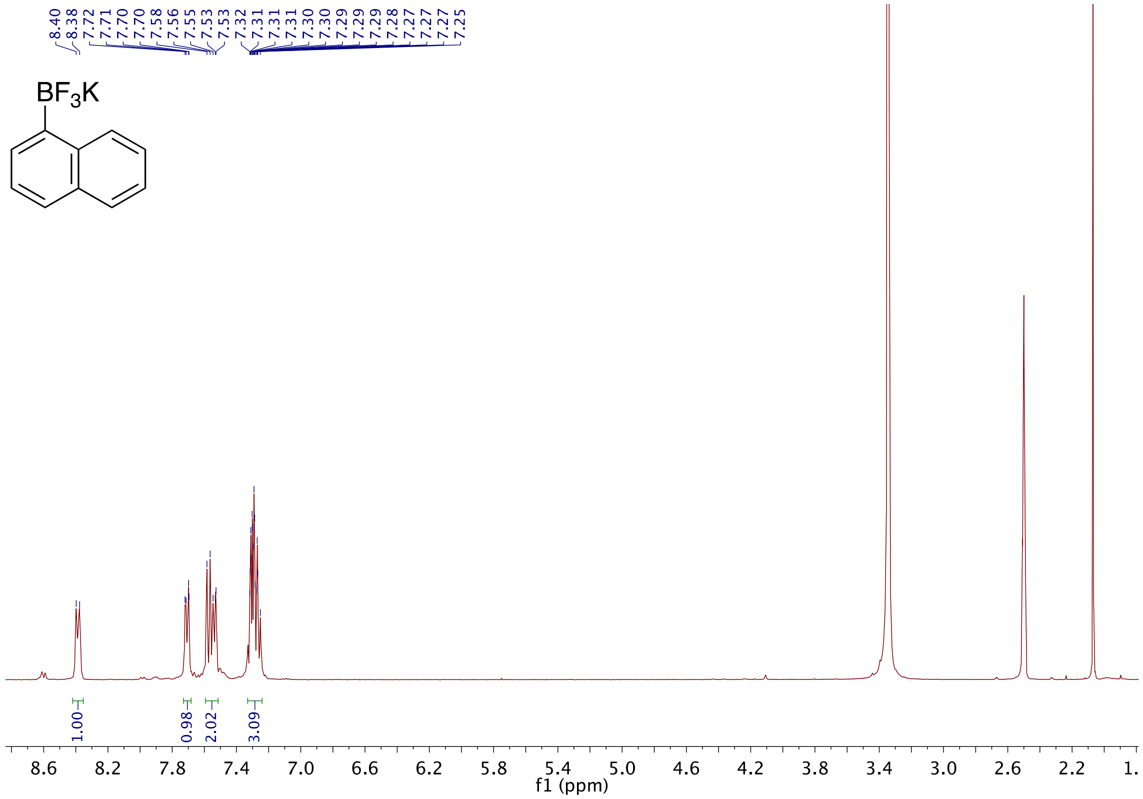
8.40
8.38
7.72
7.71
7.70
7.70
7.58
7.56
7.55
7.53
7.53
7.32
7.31
7.31
7.31
7.30
7.30
7.29
7.29
7.29
7.28
7.27
7.27
7.27
7.25
BF_3K
1.00
0.98
2.02
3.09
f1 (ppm)

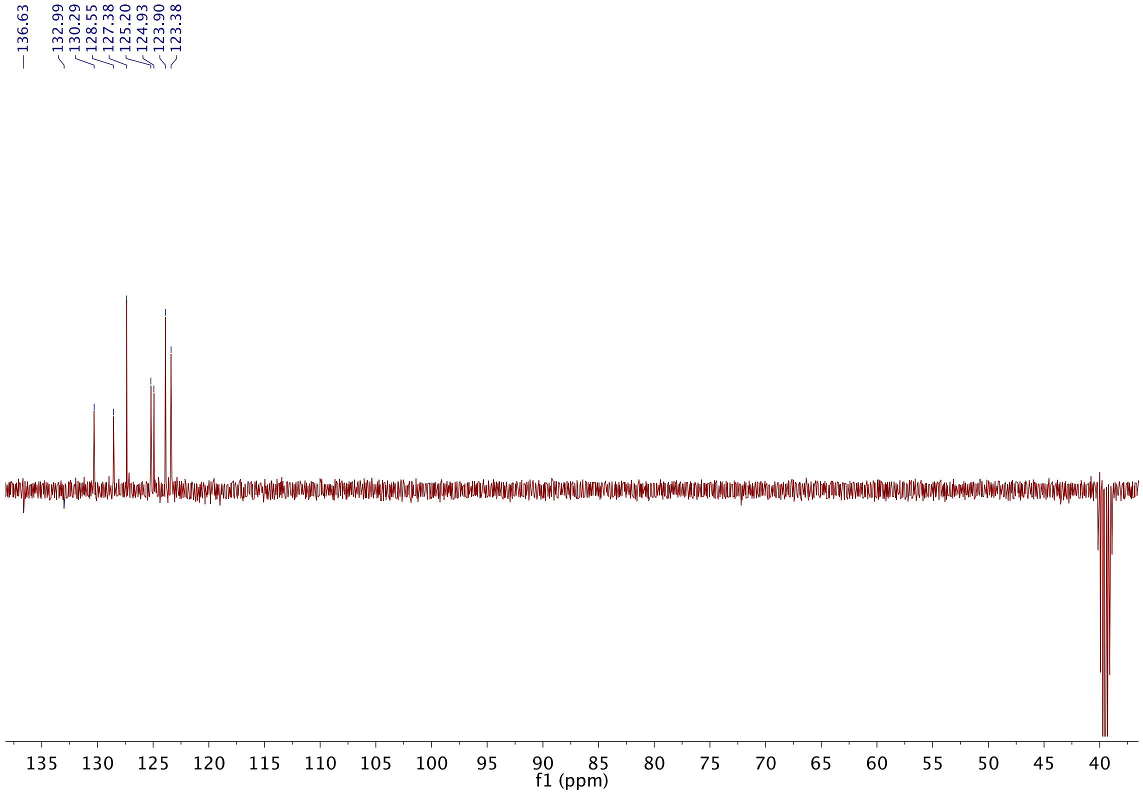
136.63
132.99
130.29
128.55
127.38
125.20
124.93
123.90
123.38
f1 (ppm)

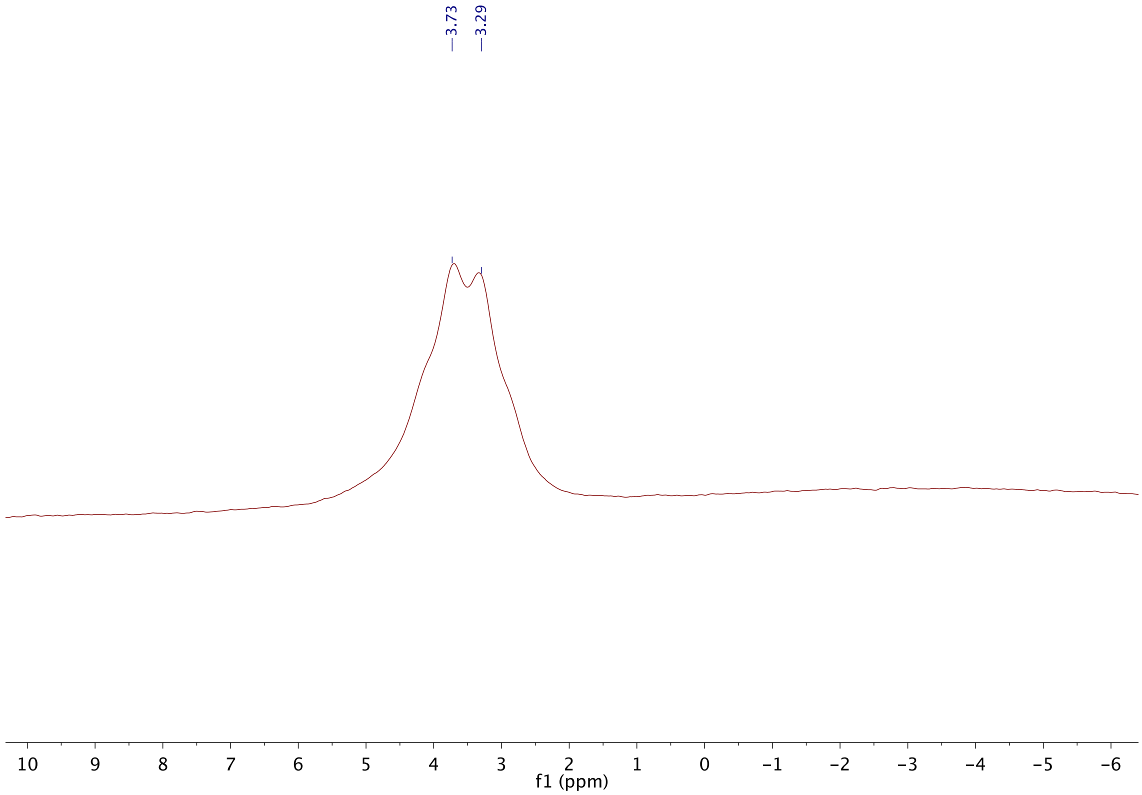

3.73
3.29
10 9 8 7 6 5 4 3 2 1 0 −1 −2 −3 −4 −5 −6
f1 (ppm)

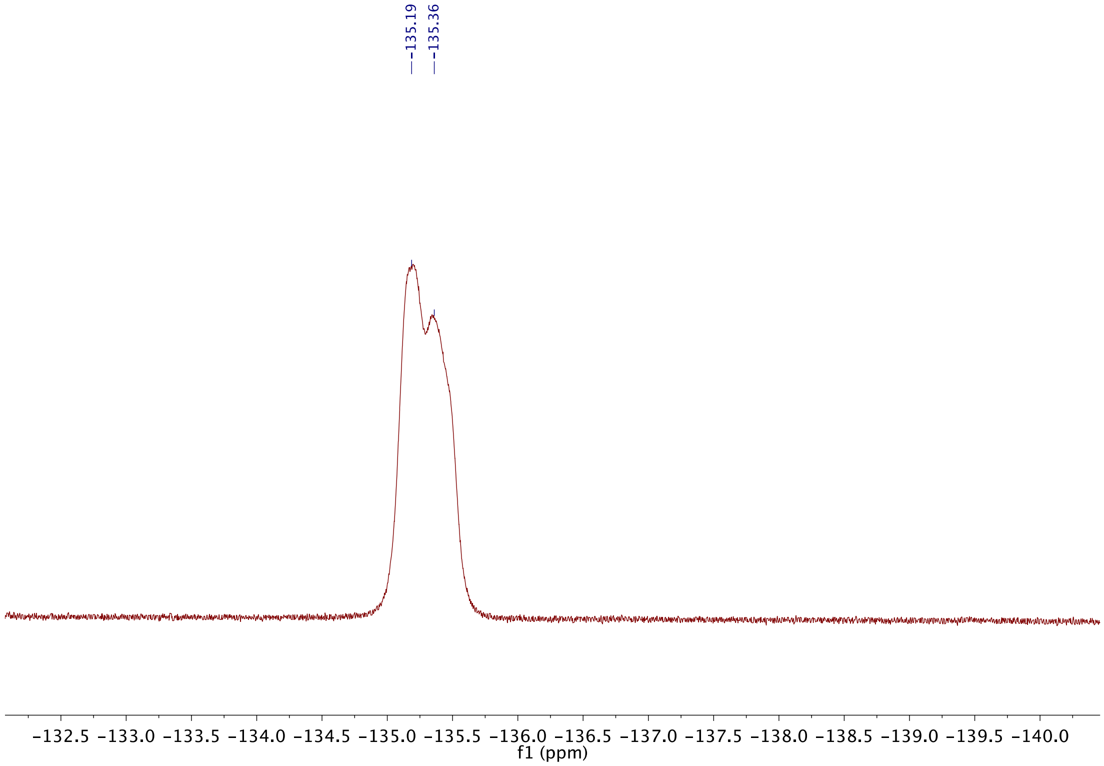

−135.19
−135.36
−132.5 −133.0 −133.5 −134.0 −134.5 −135.0 −135.5 −136.0 −136.5 −137.0 −137.5 −138.0 −138.5 −139.0 −139.5 −140.0
f1 (ppm)

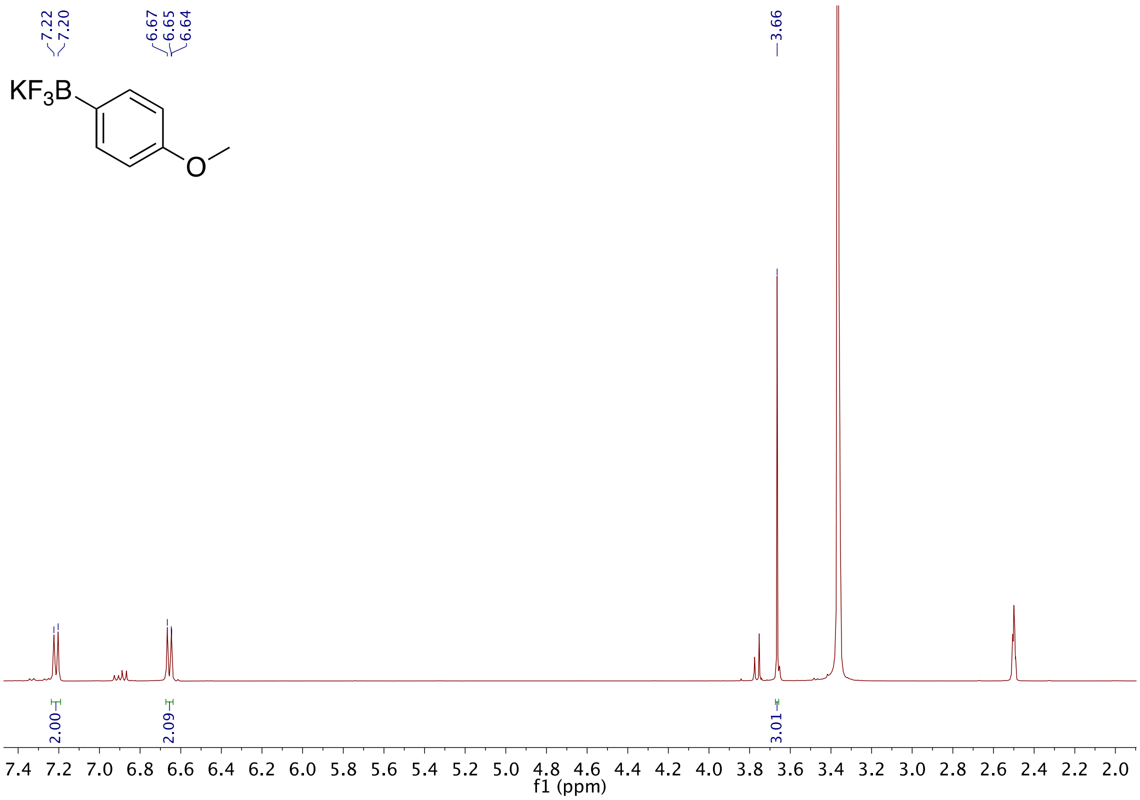
7.22
7.20
6.67
6.65
6.64
3.66
KF3B
O
2.00
2.09
3.01
f1 (ppm)

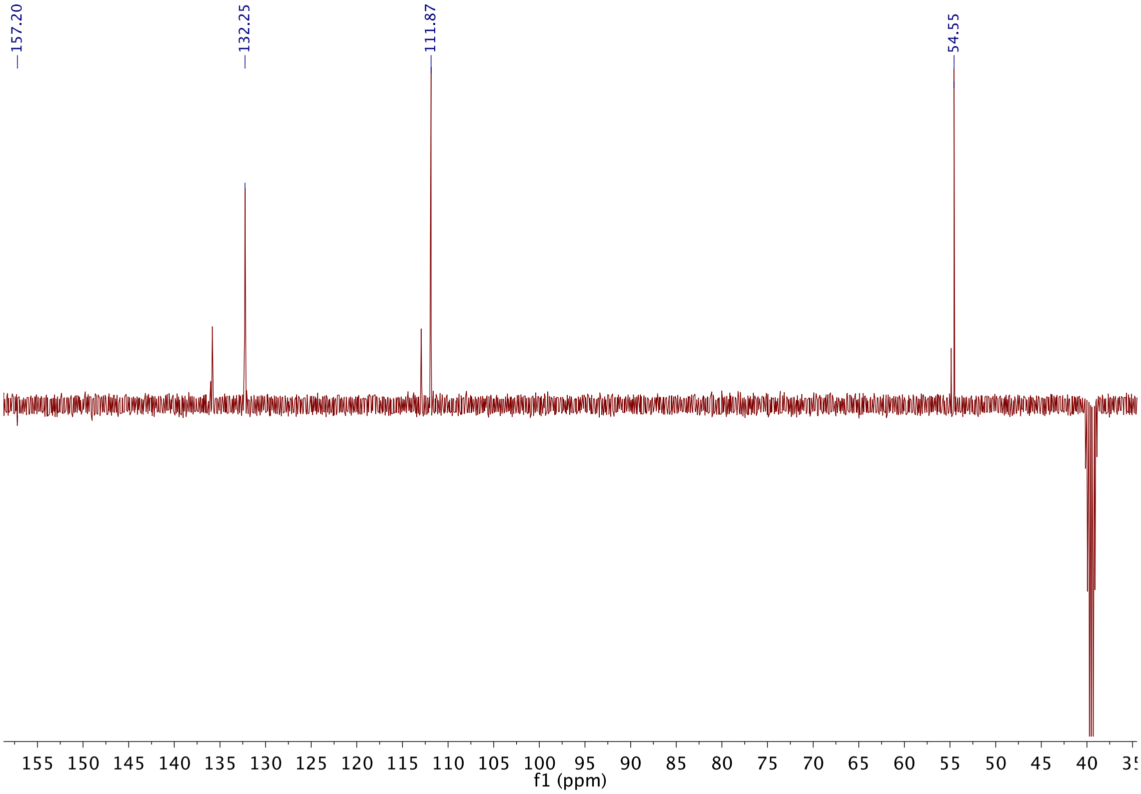
157.20
132.25
111.87
54.55
f1 (ppm)

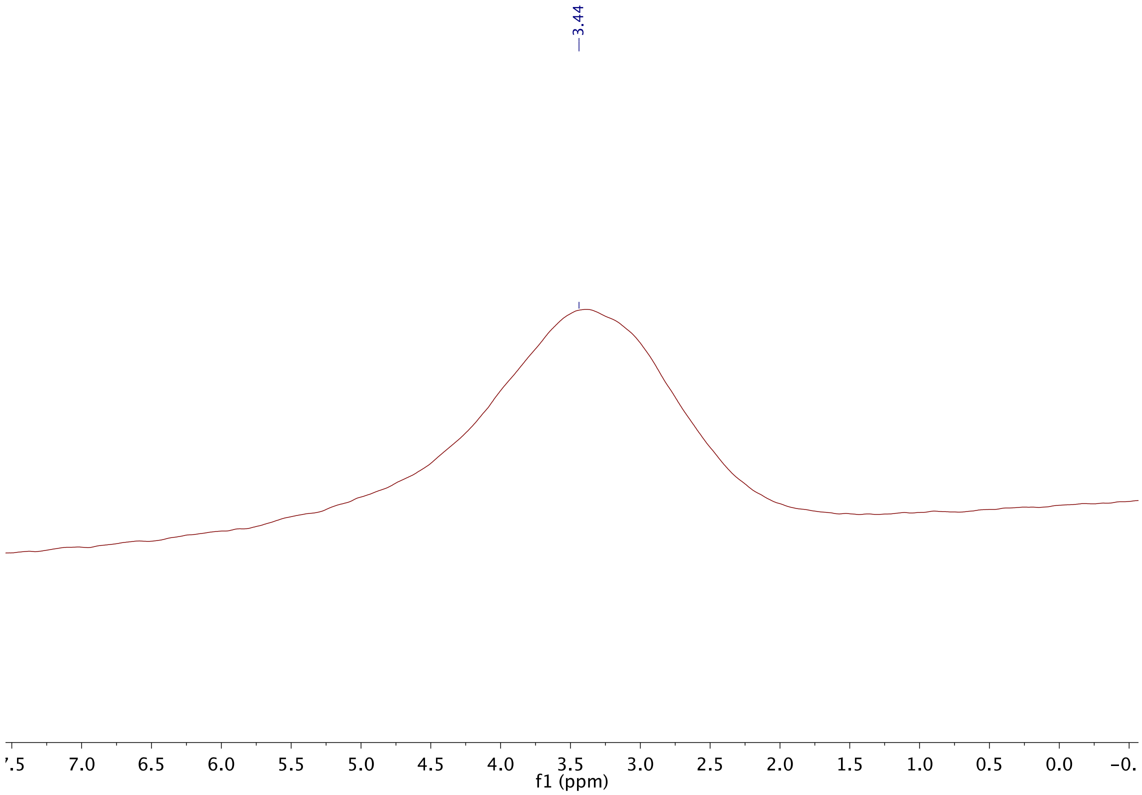

3.44
7.5
7.0
6.5
6.0
5.5
5.0
4.5
4.0
3.5
3.0
2.5
2.0
1.5
1.0
0.5
0.0
f1 (ppm)

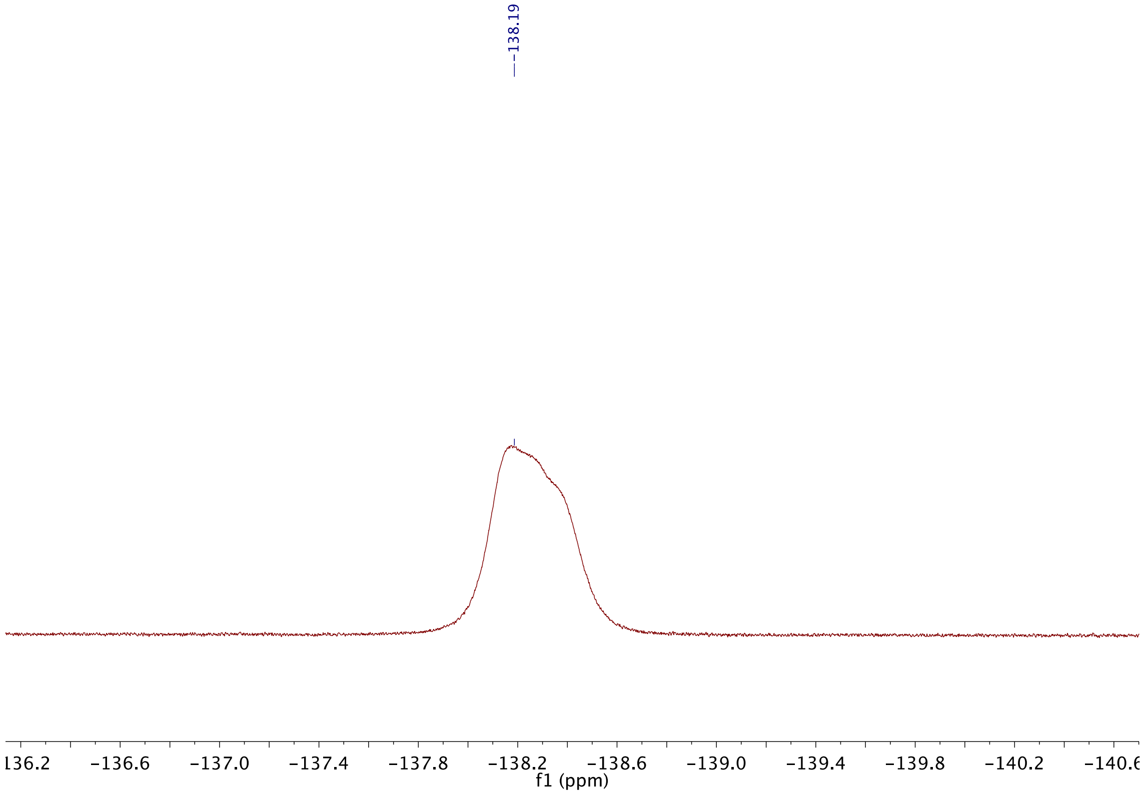

−138.19
−136.6
−137.0
−137.4
−137.8
−138.2
−138.6
−139.0
−139.4
−139.8
−140.2
f1 (ppm)

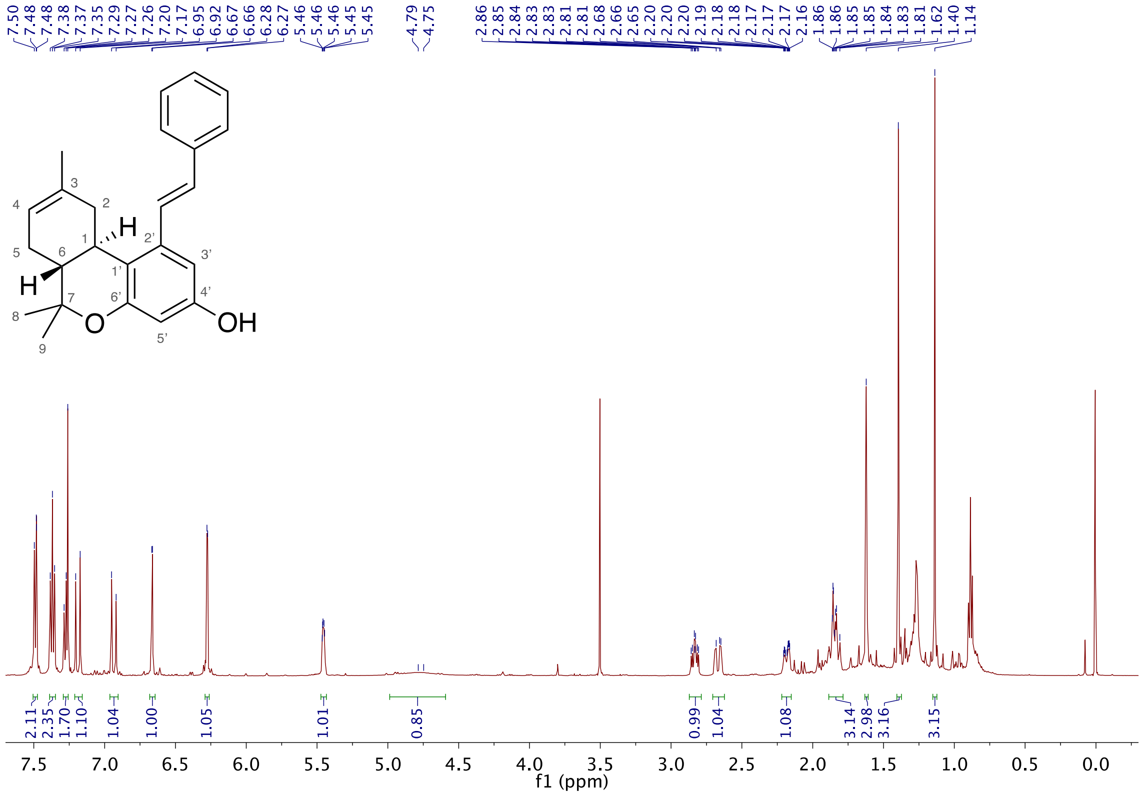
7.50 7.48 7.48 7.38 7.37 7.35 7.29 7.27 7.26 7.20 7.17 6.95 6.92 6.67 6.66 6.28 6.27 5.46 5.46 5.46 5.45 5.45 4.79 4.75 2.86 2.85 2.84 2.83 2.83 2.81 2.81 2.68 2.66 2.65 2.20 2.20 2.19 2.18 2.18 2.17 2.17 2.16 1.86 1.86 1.85 1.85 1.84 1.83 1.81 1.62 1.40 1.14
3
2
4
1
H
2'
5
6
3'
1'
H
7
6'
4'
8
O
OH
9
5'
2.11 2.35 1.70 1.10 1.04 1.00 1.05 1.01 0.85 0.99 1.04 1.08 3.14 2.98 3.16 3.15
7.5 7.0 6.5 6.0 5.5 5.0 4.5 4.0 3.5 3.0 2.5 2.0 1.5 1.0 0.5 0.0
f1 (ppm)

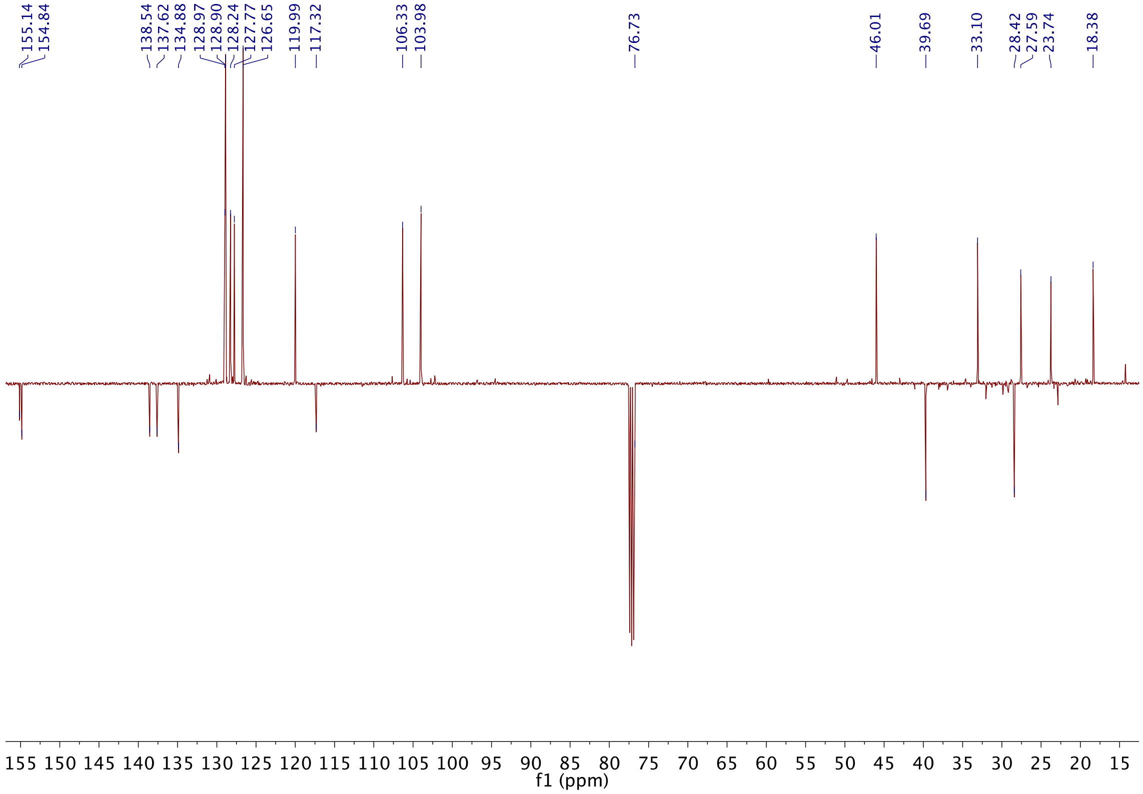
155.14 154.84 138.54 137.62 134.88 128.97 128.90 128.24 127.77 126.65 119.99 117.32 106.33 103.98 76.73 46.01 39.69 33.10 28.42 27.59 23.74 18.38
155 150 145 140 135 130 125 120 115 110 105 100 95 90 85 80 75 70 65 60 55 50 45 40 35 30 25 20 15
f1 (ppm)

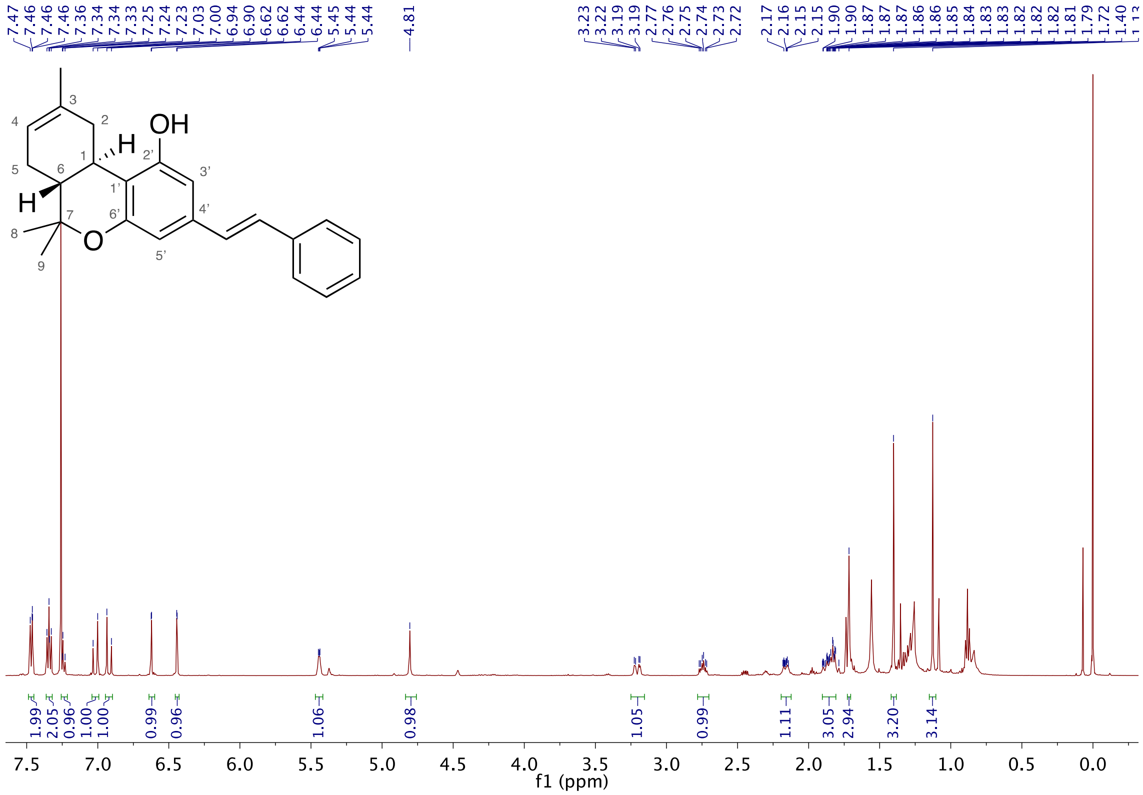
7.47
7.46
7.46
7.46
7.36
7.34
7.34
7.33
7.25
7.24
7.23
7.03
7.00
6.94
6.90
6.62
6.62
6.44
6.44
5.45
5.44
5.44
4.81
3.23
3.22
3.19
3.19
2.77
2.76
2.75
2.74
2.73
2.72
2.17
2.16
2.15
2.15
1.90
1.90
1.87
1.87
1.87
1.86
1.85
1.84
1.83
1.83
1.82
1.82
1.82
1.81
1.79
1.72
1.40
OH
H
H
O
1.99
2.05
0.96
1.00
1.00
0.99
0.96
1.06
0.98
1.05
0.99
1.11
3.05
2.94
3.20
3.14
7.5 7.0 6.5 6.0 5.5 5.0 4.5 4.0 3.5 3.0 2.5 2.0 1.5 1.0 0.5 0.0
f1 (ppm)

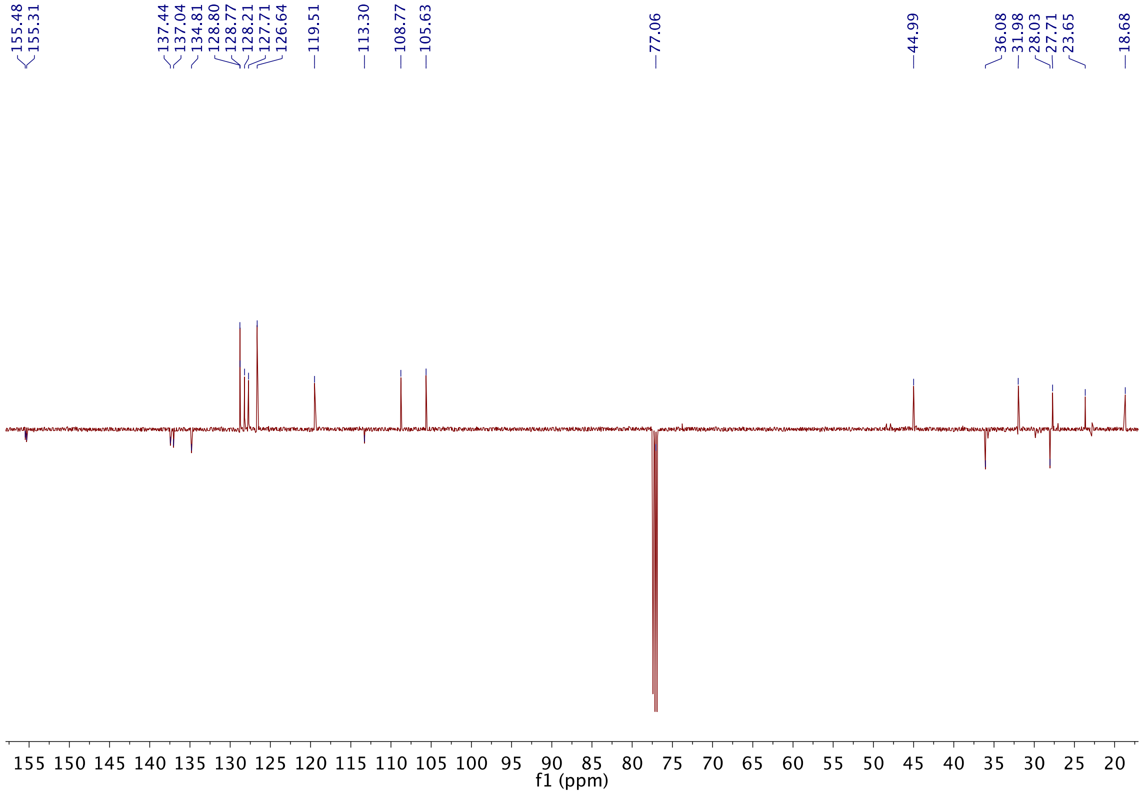
155.48
155.31
137.44
137.04
134.81
128.80
128.77
128.21
127.71
126.64
119.51
113.30
108.77
105.63
77.06
44.99
36.08
31.98
28.03
27.71
23.65
18.68
155 150 145 140 135 130 125 120 115 110 105 100 95 90 85 80 75 70 65 60 55 50 45 40 35 30 25 20
f1 (ppm)

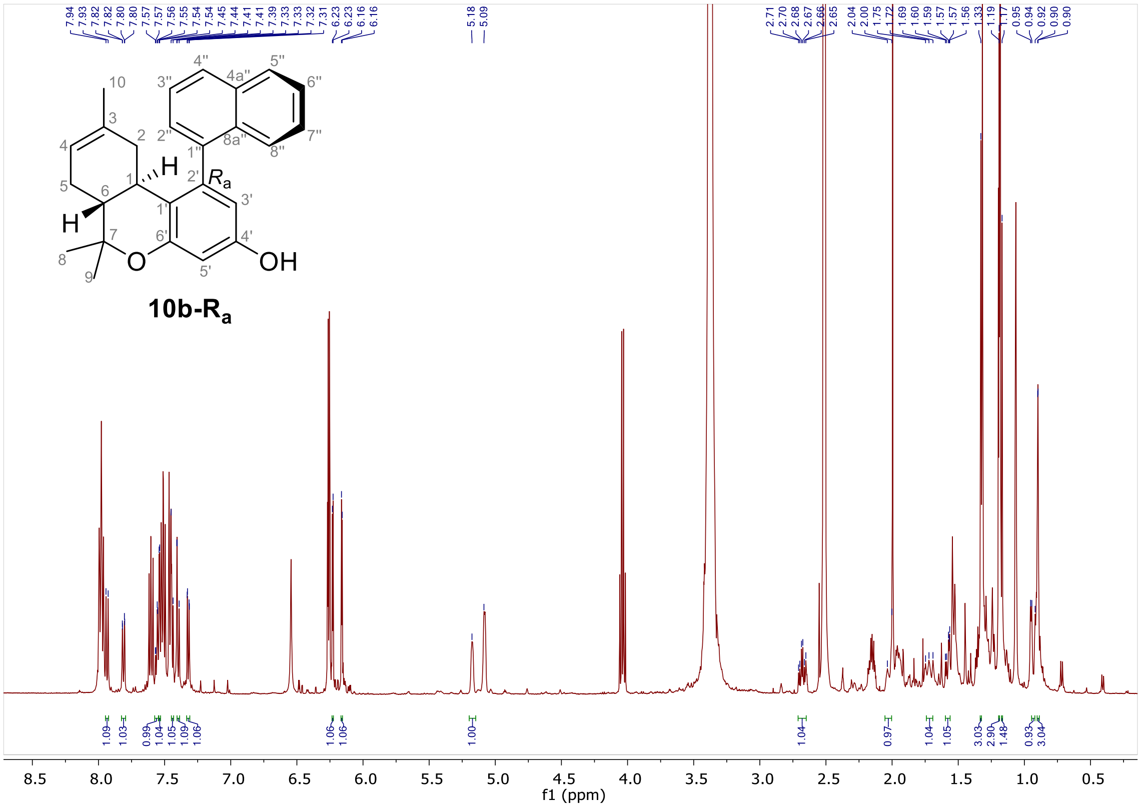

7.94 7.93 7.82 7.82 7.80 7.80 7.57 7.57 7.56 7.55 7.54 7.54 7.45 7.44 7.41 7.39 7.33 7.33 7.32 7.31 6.23 6.23 6.16 6.16
5.18 5.09
2.71 2.70 2.68 2.67 2.66 2.65 2.04 2.00 1.75 1.72 1.69 1.60 1.59 1.57 1.57 1.56 1.33 1.19 1.17 0.95 0.94 0.92 0.90 0.90
10b-Ra
1.09 1.03 0.99 1.04 1.05 1.09 1.06 1.06 1.06 1.00 1.04 0.97 1.04 1.05 3.03 2.90 1.48 0.93 3.04
f1 (ppm)

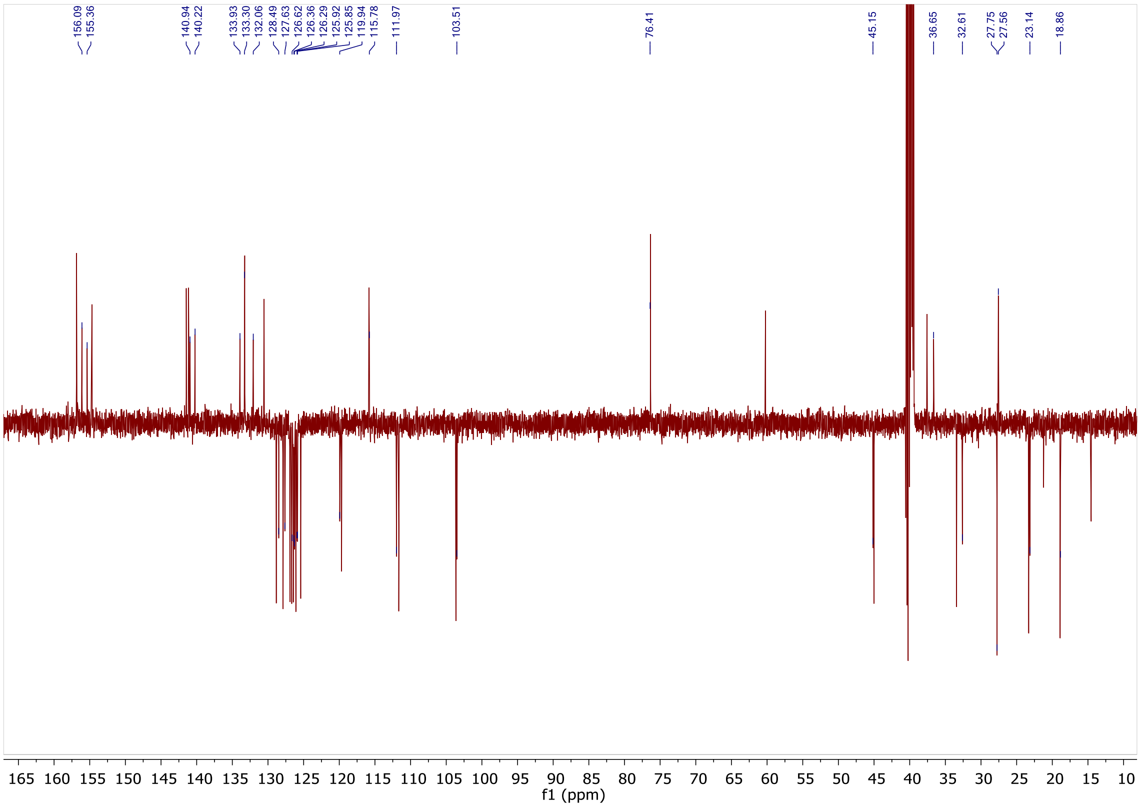

156.09 155.36 140.94 140.22 133.93 133.30 132.06 128.49 127.63 126.62 126.36 126.29 125.92 125.85 119.94 115.78 111.97 103.51 76.41 45.15 36.65 32.61 27.75 27.56 23.14 18.86
f1 (ppm)

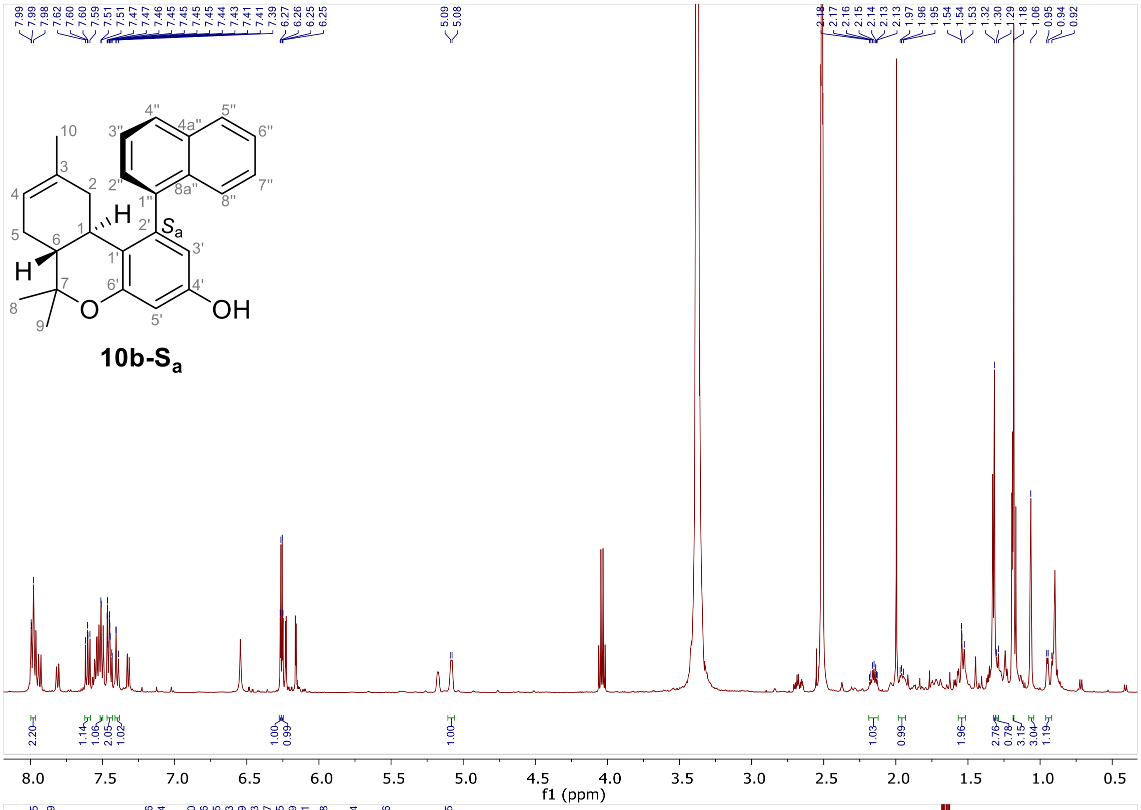
10b-S$_a$
f1 (ppm)

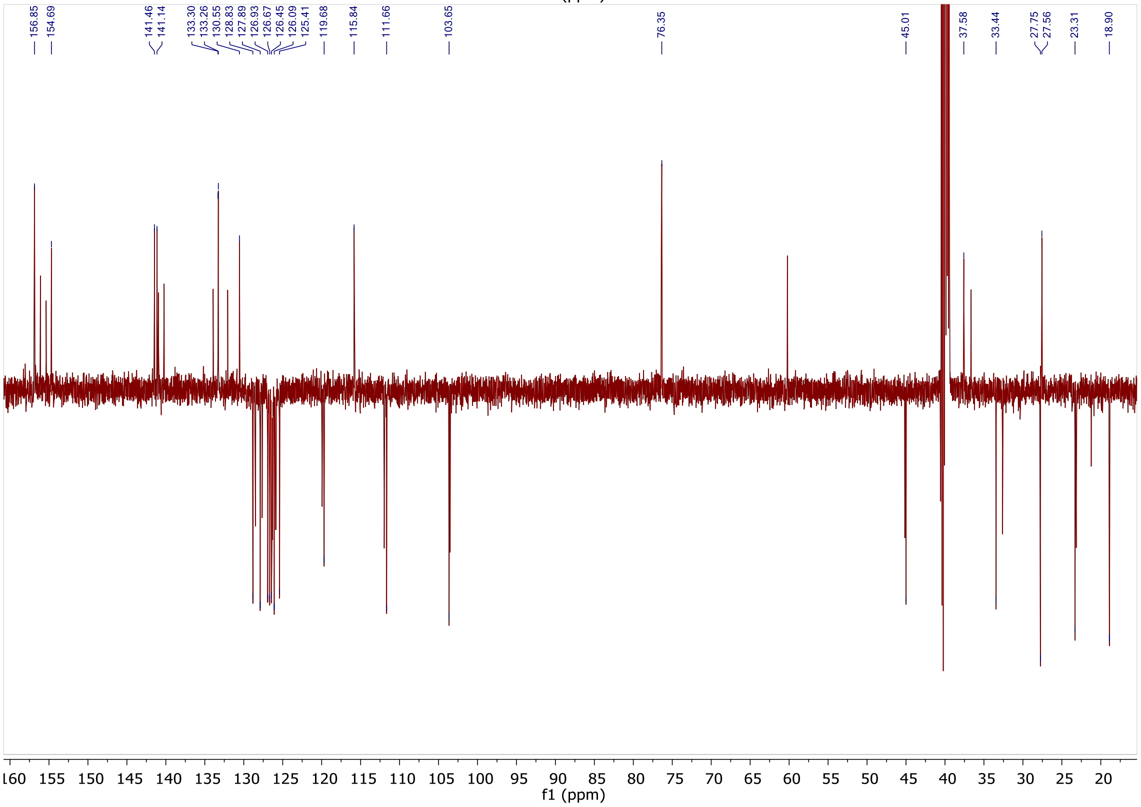
f1 (ppm)

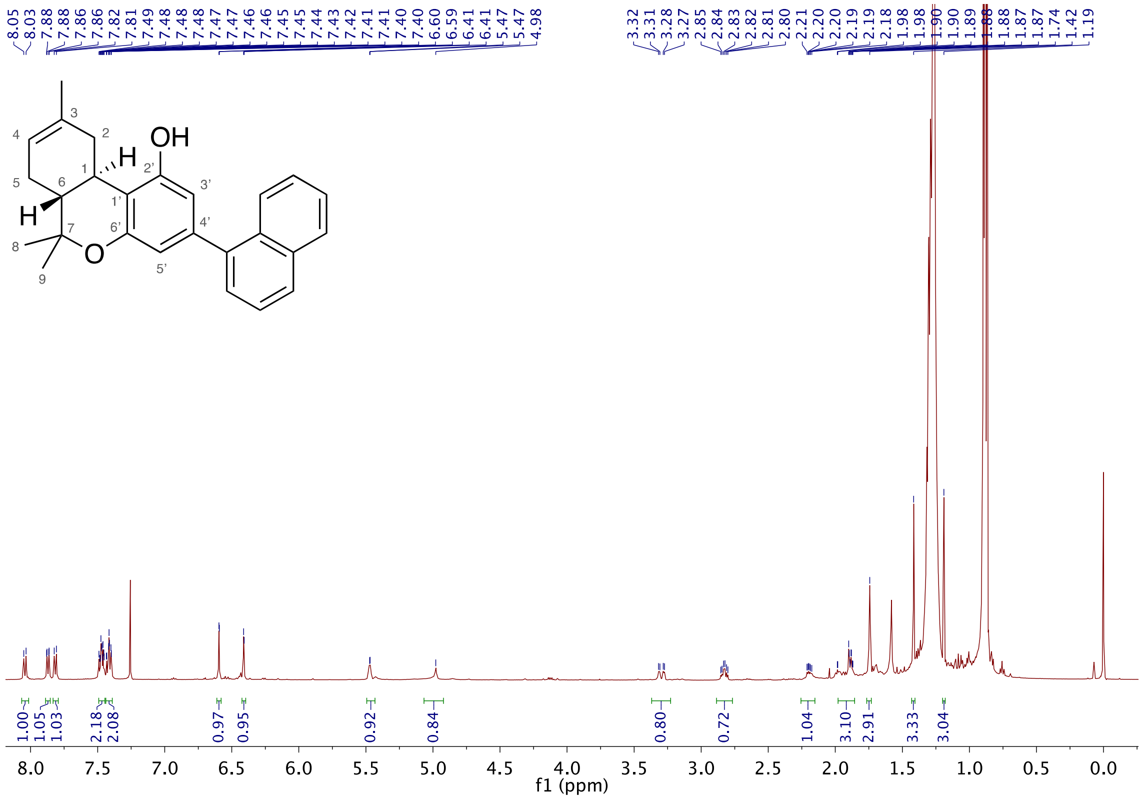
8.05 8.03 7.88 7.88 7.86 7.86 7.82 7.81 7.49 7.48 7.48 7.48 7.47 7.47 7.46 7.46 7.45 7.45 7.44 7.43 7.42 7.41 7.41 7.40 7.40 6.60 6.59 6.41 6.41 5.47 5.47 4.98
3.32 3.31 3.28 3.27 2.85 2.84 2.83 2.82 2.81 2.80 2.21 2.20 2.20 2.19 2.19 2.18 1.98 1.98 1.90 1.90 1.89 1.88 1.88 1.87 1.87 1.74 1.42 1.19
OH
H
H
O
1 2 3 4 5 6 7 8 9 1' 2' 3' 4' 5' 6'
1.00 1.05 1.03 2.18 2.08 0.97 0.95 0.92 0.84 0.80 0.72 1.04 3.10 2.91 3.33 3.04
8.0 7.5 7.0 6.5 6.0 5.5 5.0 4.5 4.0 3.5 3.0 2.5 2.0 1.5 1.0 0.5 0.0
f1 (ppm)

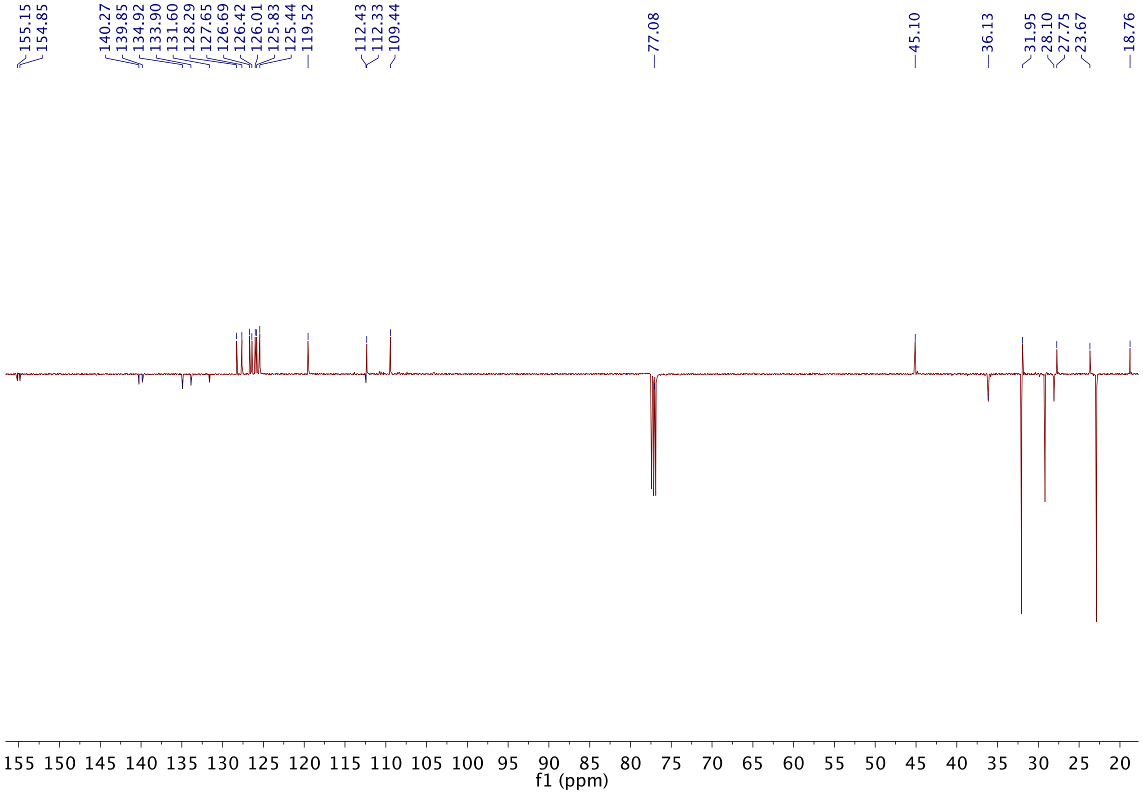
155.15 154.85 140.27 139.85 134.92 133.90 131.60 128.29 127.65 126.69 126.42 126.01 125.83 125.44 119.52 112.43 112.33 109.44 77.08 45.10 36.13 31.95 28.10 27.75 23.67 18.76
155 150 145 140 135 130 125 120 115 110 105 100 95 90 85 80 75 70 65 60 55 50 45 40 35 30 25 20
f1 (ppm)

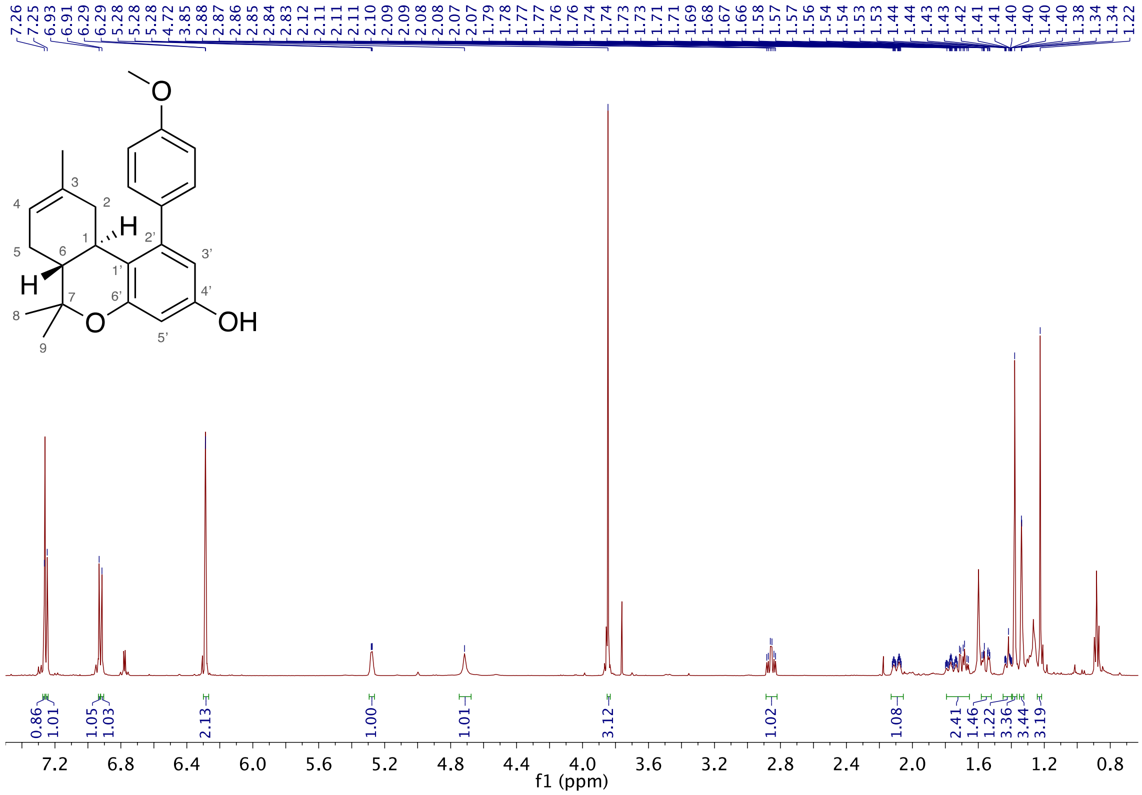
7.26
7.25
6.93
6.91
6.29
6.29
5.28
5.28
5.28
4.72
3.85
2.88
2.87
2.86
2.85
2.84
2.83
2.12
2.11
2.11
2.11
2.10
2.09
2.09
2.08
2.08
2.07
2.07
1.79
1.78
1.77
1.77
1.76
1.76
1.74
1.74
1.73
1.73
1.71
1.71
1.69
1.68
1.67
1.66
1.58
1.57
1.57
1.56
1.56
1.54
1.54
1.53
1.53
1.53
1.44
1.44
1.43
1.43
1.42
1.41
1.41
1.40
1.40
1.40
1.40
1.38
1.34
1.22
O
OH
H
H
1 2 3 4 5 6 7 8 9
1' 2' 3' 4' 5' 6'
0.86
1.01
1.05
1.03
2.13
1.00
1.01
3.12
1.02
1.08
2.41
1.46
1.22
3.36
3.44
3.19
7.2 6.8 6.4 6.0 5.6 5.2 4.8 4.4 4.0 3.6 3.2 2.8 2.4 2.0 1.6 1.2 0.8
f1 (ppm)

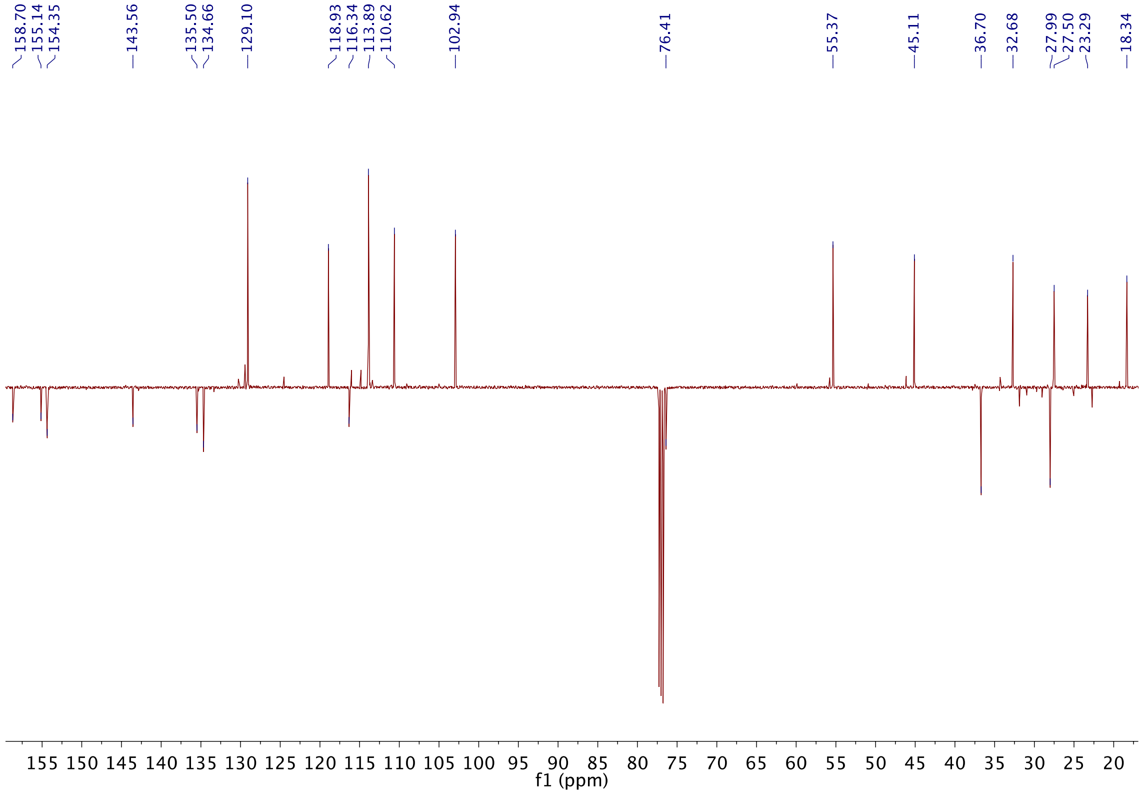
158.70
155.14
154.35
143.56
135.50
134.66
129.10
118.93
116.34
113.89
110.62
102.94
76.41
55.37
45.11
36.70
32.68
27.99
27.50
23.29
18.34
155 150 145 140 135 130 125 120 115 110 105 100 95 90 85 80 75 70 65 60 55 50 45 40 35 30 25 20
f1 (ppm)

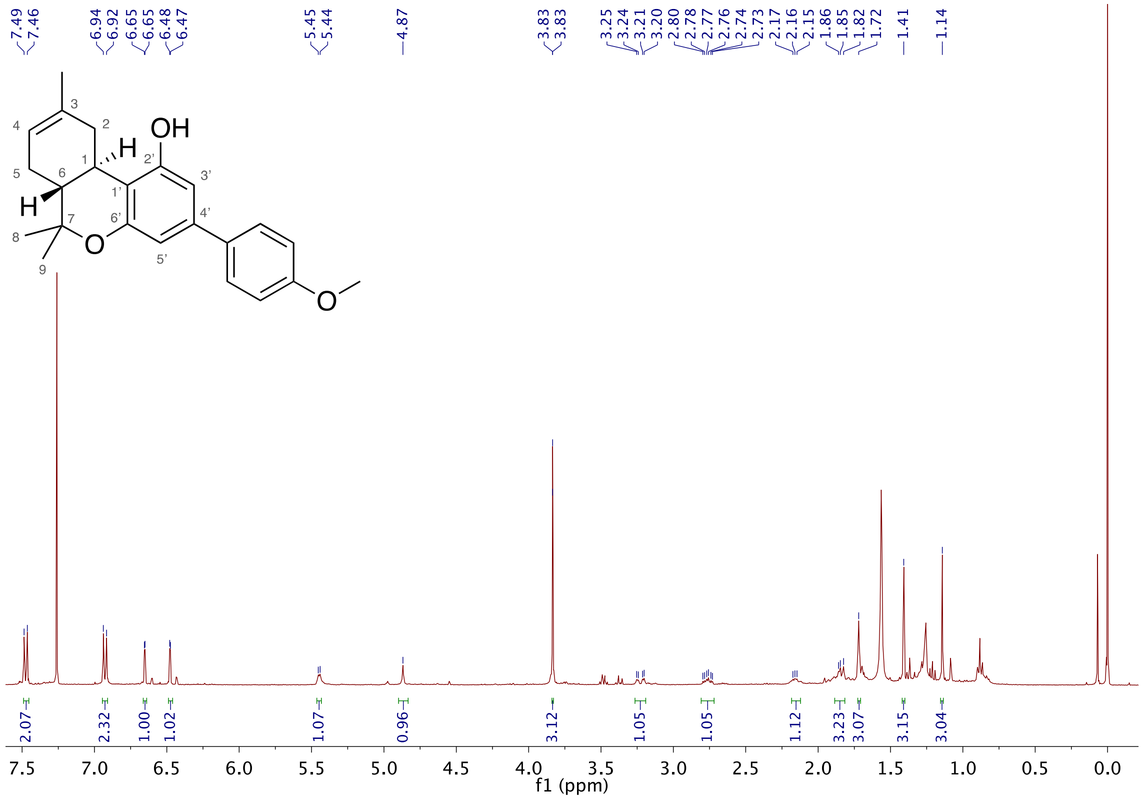
7.49
7.46
6.94
6.92
6.65
6.65
6.48
6.47
5.45
5.44
4.87
3.83
3.83
3.25
3.24
3.21
3.20
2.80
2.78
2.77
2.76
2.74
2.73
2.17
2.16
2.15
1.86
1.85
1.82
1.72
1.41
1.14
OH
H
H
O
O
2.07
2.32
1.00
1.02
1.07
0.96
3.12
1.05
1.05
1.12
3.23
3.07
3.15
3.04
f1 (ppm)

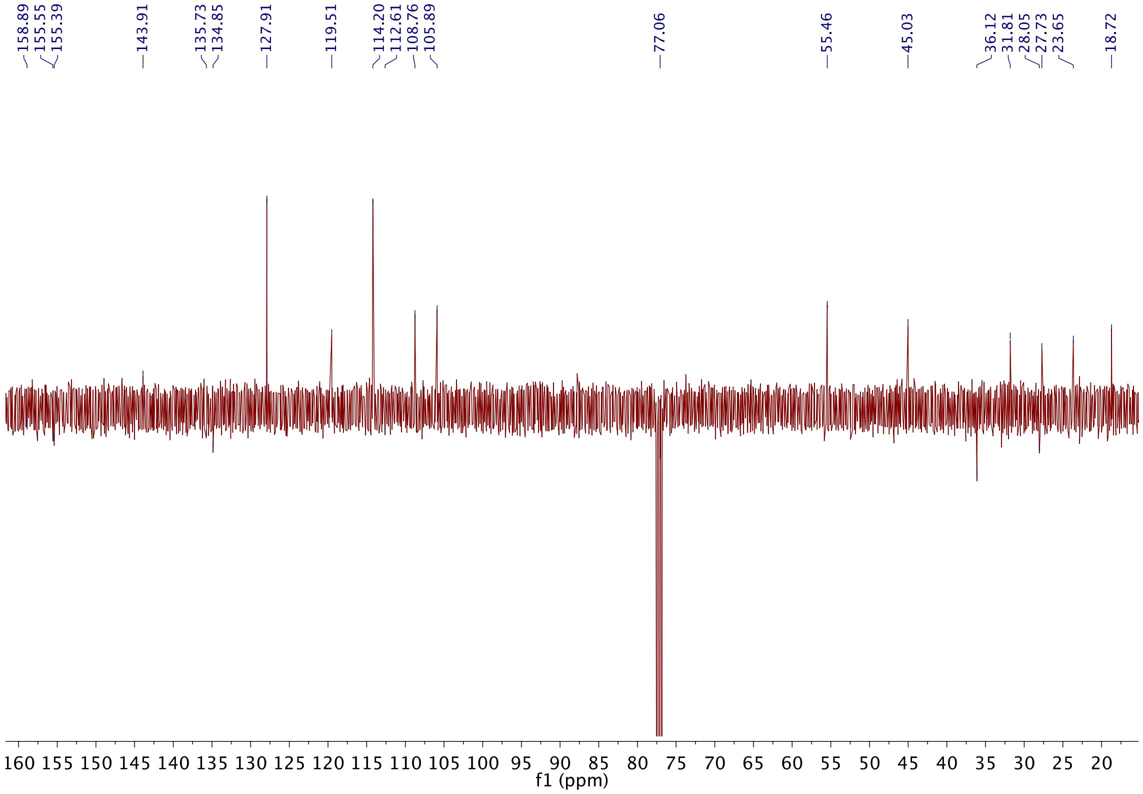
158.89
155.55
155.39
143.91
135.73
134.85
127.91
119.51
114.20
112.61
108.76
105.89
77.06
55.46
45.03
36.12
31.81
28.05
27.73
23.65
18.72
f1 (ppm)

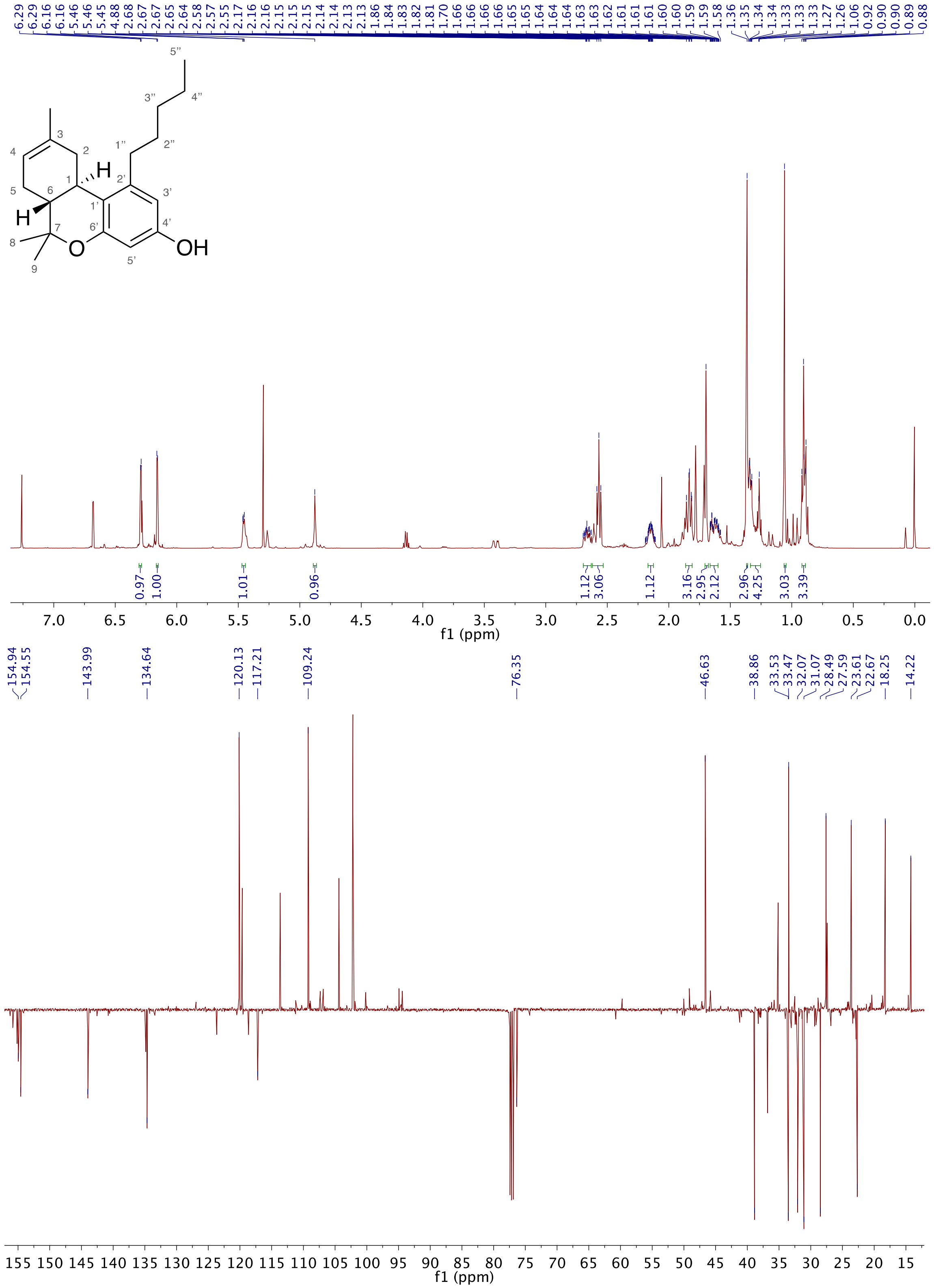

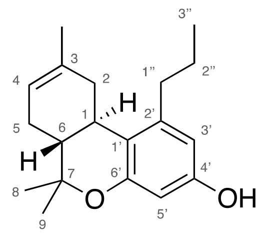

3''
2''
1''
2
3
4
5
6
1
2'
3'
1'
4'
6'
7
8
9
5'
H
H
O
OH

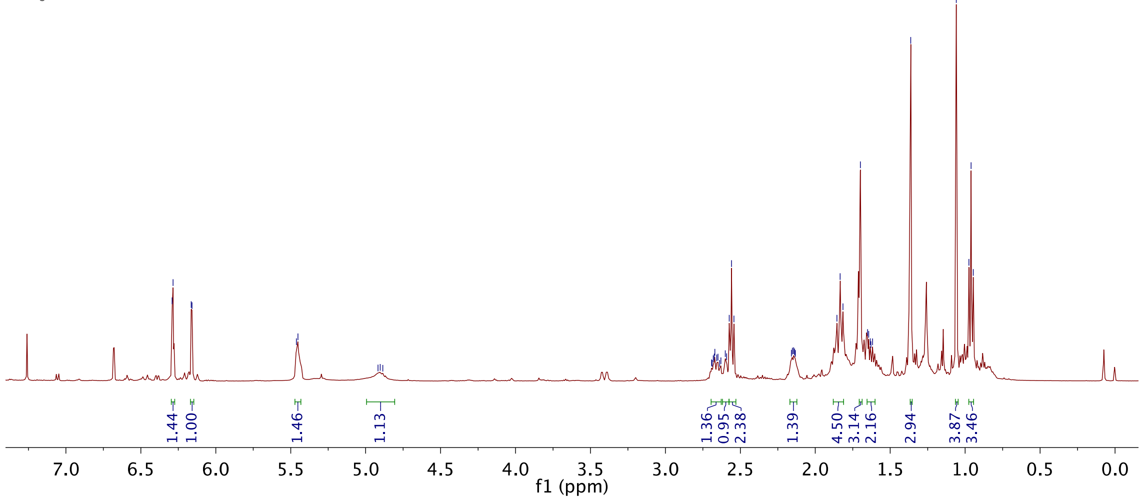

6.29
6.29
6.16
6.16
5.46
5.45
4.92
4.90
4.89
2.69
2.69
2.68
2.67
2.67
2.66
2.65
2.64
2.63
2.60
2.59
2.57
2.56
2.54
2.16
2.15
2.14
2.14
2.13
1.86
1.83
1.82
1.70
1.65
1.64
1.63
1.63
1.62
1.36
1.06
0.97
0.96
0.95
1.44
1.00
1.46
1.13
1.36
0.95
2.38
1.39
4.50
3.14
2.16
2.94
3.87
3.46
7.0 6.5 6.0 5.5 5.0 4.5 4.0 3.5 3.0 2.5 2.0 1.5 1.0 0.5 0.0
f1 (ppm)

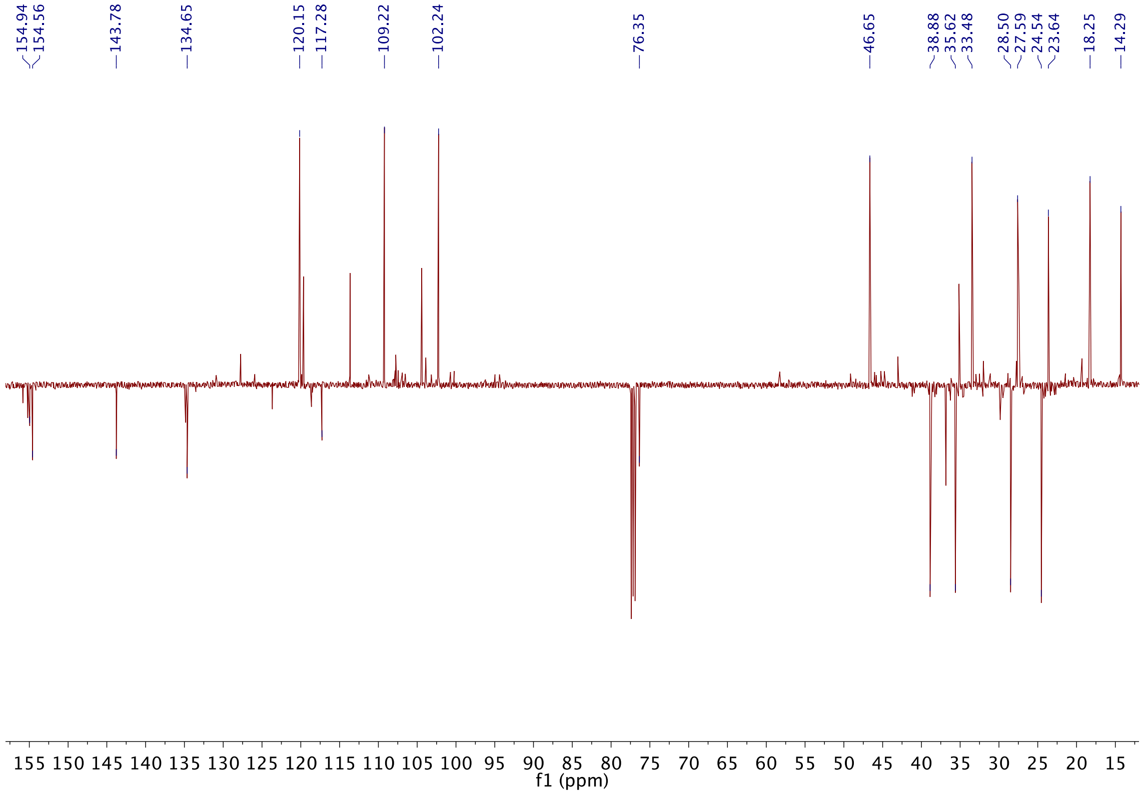

154.94
154.56
143.78
134.65
120.15
117.28
109.22
102.24
76.35
46.65
38.88
35.62
33.48
28.50
27.59
24.54
23.64
18.25
14.29
155 150 145 140 135 130 125 120 115 110 105 100 95 90 85 80 75 70 65 60 55 50 45 40 35 30 25 20 15
f1 (ppm)
